# Genome-wide analyses identify 25 infertility loci and relationships with reproductive traits across the allele frequency spectrum

**DOI:** 10.1038/s41588-025-02156-8

**Published:** 2025-04-14

**Authors:** Samvida S. Venkatesh, Laura B. L. Wittemans, Duncan S. Palmer, Nikolas A. Baya, Teresa Ferreira, Barney Hill, Frederik Heymann Lassen, Melody J. Parker, Saskia Reibe, Ahmed Elhakeem, Karina Banasik, Mie T. Bruun, Christian Erikstrup, Bitten Aagard Jensen, Anders Juul, Christina Mikkelsen, Henriette S. Nielsen, Sisse R. Ostrowski, Ole B. Pedersen, Palle Duun Rohde, Erik Sørensen, Henrik Ullum, David Westergaard, Asgeir Haraldsson, Hilma Holm, Ingileif Jonsdottir, Isleifur Olafsson, Thora Steingrimsdottir, Valgerdur Steinthorsdottir, Gudmar Thorleifsson, Jessica Figueredo, Minna K. Karjalainen, Anu Pasanen, Benjamin M. Jacobs, Georgios Kalantzis, Nikki Hubers, Genes & Health Research Team, David A. van Heel, Estonian Biobank Research Team, Reedik Mägi, Andres Metspalu, Lili Milani, Tõnu Esko, Mari Nelis, Georgi Hudjashov, Triin Laisk, Estonian Health Informatics Research Team, Raivo Kolde, Sven Laur, Sulev Reisberg, Jaak Vilo, DBDS Genomic Consortium, Karina Banasik, Mie T. Bruun, Christian Erikstrup, Christina Mikkelsen, Henriette S. Nielsen, Sisse R. Ostrowski, Ole B. Pedersen, Palle Duun Rohde, Erik Sørensen, Henrik Ullum, David Westergaard, Bitten Aagard Jensen, Mette Nyegaard, Kari Stefansson, FinnGen, Minna K. Karjalainen, Hannele Laivuori, Margaret Lippincott, Abigail Fraser, Deborah A. Lawlor, Nicholas J. Timpson, Mette Nyegaard, Kari Stefansson, Reedik Magi, Hannele Laivuori, David A. van Heel, Dorret I. Boomsma, Ravikumar Balasubramanian, Stephanie B. Seminara, Yee-Ming Chan, Triin Laisk, Cecilia M. Lindgren

**Affiliations:** 1 Big Data Institute, Li Ka Shing Centre for Health Information and Discovery, University of Oxford, Oxford, UK; 2 Wellcome Centre for Human Genetics, Nuffield Department of Medicine, University of Oxford, Oxford, UK; 3 Novo Nordisk Research Centre Oxford, Oxford, UK; 4 Nuffield Department of Women’s and Reproductive Health, Medical Sciences Division, University of Oxford, Oxford, UK; 5 Nuffield Department of Population Health, Medical Sciences Division, University of Oxford, Oxford, UK; 6 Nuffield Department of Clinical Medicine, University of Oxford, John Radcliffe Hospital, Oxford, UK; 7 MRC Integrative Epidemiology Unit at the University of Bristol, Bristol, UK; 8 Population Health Science, Bristol Medical School, University of Bristol, Bristol, UK; 9 Novo Nordisk Foundation Center for Protein Research, University of Copenhagen, Copenhagen, Denmark; 10 Department of Obstetrics and Gynecology, Copenhagen University Hospital, Hvidovre, Copenhagen, Denmark; 11 Department of Clinical Immunology, Odense University Hospital, Odense, Denmark; 12 Department of Clinical Immunology, Aarhus University Hospital, Aarhus, Denmark; 13 Department of Clinical Medicine, Health, Aarhus University, Aarhus, Denmark; 14 Department of Clinical Immunology, Aalborg University Hospital, Aalborg, Denmark; 15 Department of Clinical Medicine, Faculty of Health and Medical Sciences, University of Copenhagen, Copenhagen, Denmark; 16 Department of Growth and Reproduction, Copenhagen University Hospital–Rigshospitalet, Copenhagen, Denmark; 17 Department of Clinical Immunology, Copenhagen University Hospital–Rigshospitalet, Copenhagen, Denmark; 18 Novo Nordisk Foundation Center for Basic Metabolic Research, Faculty of Health and Medical Science, Copenhagen University, Copenhagen, Denmark; 19 Department of Obstetrics and Gynecology, The Fertility Clinic, Hvidovre University Hospital, Copenhagen, Denmark; 20 Department of Clinical Immunology, Zealand University Hospital−Køge, Køge, Denmark; 21 Genomic Medicine, Department of Health Science and Technology, Aalborg University, Aalborg, Denmark; 22 Statens Serum Institut, Copenhagen, Denmark; 23 Faculty of Medicine, University of Iceland, Reykjavik, Iceland; 24 Children’s Hospital Iceland, Landspitali University Hospital, Reykjavik, Iceland; 25 deCODE genetics/Amgen Inc., Reykjavik, Iceland; 26 Department of Clinical Biochemistry, Landspitali University Hospital, Reykjavik, Iceland; 27 Department of Obstetrics and Gynecology, Landspitali University Hospital, Reykjavik, Iceland; 28 Estonian Genome Centre, Institute of Genomics, University of Tartu, Tartu, Estonia; 29 Institute for Molecular Medicine Finland, Helsinki Institute of Life Science, University of Helsinki, Helsinki, Finland; 30 Research Unit of Population Health, Faculty of Medicine, University of Oulu, Oulu, Finland; 31 Northern Finland Birth Cohorts, Arctic Biobank, Infrastructure for Population Studies, Faculty of Medicine, University of Oulu, Oulu, Finland; 32 Research Unit of Clinical Medicine, Medical Research Center Oulu, University of Oulu, and Department of Children and Adolescents, Oulu University Hospital, Oulu, Finland; 33 Centre for Preventive Neurology, Wolfson Institute of Population Health, Queen Mary University London, London, UK; 34 Wellcome Sanger Institute, Wellcome Genome Campus, Hinxton, UK; 35 Department of Biological Psychology, Netherlands Twin Register, Vrije Universiteit, Amsterdam, The Netherlands; 36 Amsterdam Reproduction and Development Institute, Amsterdam, The Netherlands; 37 Department of Complex Trait Genetics, Center for Neurogenomics and Cognitive Research, Vrije Universiteit, Amsterdam, The Netherlands; 38 Reproductive Endocrine Unit, Massachusetts General Hospital, Boston, MA USA; 39 Harvard Medical School, Boston, MA USA; 40 Medical and Clinical Genetics, University of Helsinki and Helsinki University Hospital, Helsinki, Finland; 41 Department of Obstetrics and Gynecology, Tampere University Hospital, The Wellbeing Services County of Pirkanmaa, Tampere, Finland; 42 Center for Child, Adolescent, and Maternal Health Research, Faculty of Medicine and Health Technology, Tampere University, Tampere, Finland; 43 Blizard Institute, Queen Mary University London, London, UK; 44 Division of Endocrinology, Department of Pediatrics, Boston Children’s Hospital, Boston, MA USA; 45 Broad Institute of Harvard and MIT, Cambridge, MA USA; 46 Estonian Biobank, Institute of Genomics, University of Tartu, Tartu, Estonia; 47 Institute of Computer Science, University of Tartu, Tartu, Estonia; 48 STACC, Tartu, Estonia

**Keywords:** Genome-wide association studies, Population genetics, Genetics research, Infertility

## Abstract

Genome-wide association studies (GWASs) may help inform the etiology of infertility. Here, we perform GWAS meta-analyses across seven cohorts in up to 42,629 cases and 740,619 controls and identify 25 genetic risk loci for male and female infertility. We additionally identify up to 269 genetic loci associated with follicle-stimulating hormone, luteinizing hormone, estradiol and testosterone through sex-specific GWAS meta-analyses (*n* = 6,095–246,862). Exome sequencing analyses reveal that women carrying testosterone-lowering rare variants in some genes are at risk of infertility. However, we find no local or genome-wide genetic correlation between female infertility and reproductive hormones. While infertility is genetically correlated with endometriosis and polycystic ovary syndrome, we find limited genetic overlap between infertility and obesity. Finally, we show that the evolutionary persistence of infertility-risk alleles may be explained by directional selection. Taken together, we provide a comprehensive view of the genetic determinants of infertility across multiple diagnostic criteria.

## Abstract (web summary)

Genome-wide analyses identify variants associated with infertility and reproductive hormone levels but find limited polygenic overlap between reproductive hormone levels and infertility at the population level.

## Main

Infertility, defined as the inability to achieve pregnancy within 12 months of regular unprotected sexual intercourse, affects one in six couples across the globe^1^. A range of demographic, environmental and genetic factors may drive infertility, including the age-related decline of sperm and oocyte quality and quantity, infectious diseases and rare Mendelian disorders such as cystic fibrosis. However, the exact cause remains undetermined in up to 28% of couples and 40% of women with infertility^2^. Given that current treatments such as in vitro fertilization pose physical, emotional and financial burdens on couples and healthcare systems, a richer understanding of the biology and pathophysiology of infertility is urgently necessary.

Heritable women’s reproductive health diseases such as endometriosis^3^ and polycystic ovary syndrome (PCOS)^4^ are thought to be responsible for a considerable proportion of female infertility, with PCOS in particular accounting for up to 80% of cases of anovulatory infertility^4^. It is hypothesized that sex-hormone dysregulation^5,6^ and obesity^7^, which often accompany reproductive diseases, may be involved in the etiology of infertility. Yet little is known about the genetic basis of reproductive hormones and infertility, which are not well phenotyped in men or women in large studies^8,9^. Moreover, negative selection against infertility naturally limits the frequency of risk alleles in the population. Genome-wide association studies (GWASs) have thus typically queried proxy measures of fertility such as childlessness^10,11^, which may partly arise from socioeconomic and behavioral factors.

We aggregated data from a range of sources, including primary care and hospital electronic health records and self-report, across seven cohorts with over 1.5 million participants, to perform GWAS meta-analyses for male infertility and five categories of female infertility. In addition, we report results from the largest sex-specific GWASs so far for five reproductive hormones. By aggregating these data with complementary rare-variant genetic association testing, we catalog the common and rare genetic contributions to infertility and reproductive hormone levels, quantify the extent of shared genetic architecture between these traits and prioritize genes for further functional investigation of the hormonal and non-hormonal drivers of infertility.

## Results

### Genome-wide meta-analyses identify new loci for infertility

**Fig. 1:**
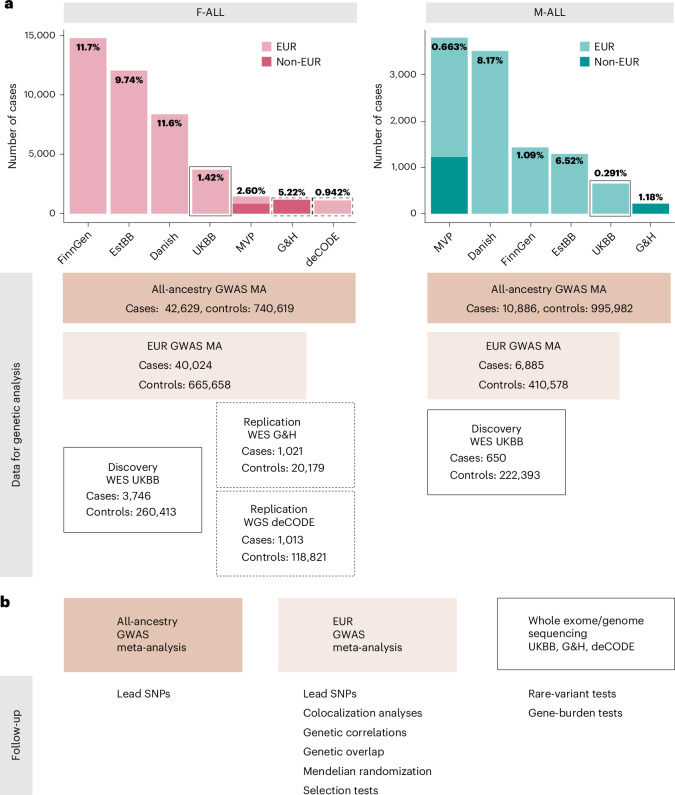
Overview of study cohorts and analyses for infertility genetic association studies. **a**, The case numbers in each cohort contributing to GWAS meta-analyses (MA) for female (left) and male (right) infertility. The prevalence of all-cause infertility in each cohort (%) is noted on the bar plots. Danish, Danish Blood Donor Study/Copenhagen Hospital Biobank. Total case and control counts for each type of genetic analysis: all-ancestry GWAS meta-analysis, EUR-only GWAS meta-analysis and WES analyses (discovery, UKBB and replication, G&H and deCODE) are displayed. Male infertility in deCODE, with <100 cases, was excluded from GWAS meta-analysis. Note the different *y*-axis scales in each subplot. **b**, Downstream analyses performed for each type of genetic analysis: lead variants were identified via distance-based pruning for all-ancestry and EUR-only GWAS meta-analyses; colocalization, genetic correlations (genome wide and local), genetic overlap and selection analyses were only performed for EUR meta-analyses due to the need for ancestry-matched LD information; rare-variant and gene-burden discovery tests were performed with WES data for the UKBB EUR-ancestry subset and replicated in individuals with WES data in G&H and whole-genome sequencing (WGS) data in deCODE.

We identified female infertility of all causes (F-ALL), anatomical causes (F-ANAT), anovulation (F-ANOV), unknown causes (that is, idiopathic infertility as defined by exclusion of known causes of infertility (anatomical or anovulatory causes, PCOS, endometriosis or uterine leiomyomas)) (F-EXCL) or idiopathic infertility defined by inclusion of diagnostic codes for idiopathic infertility (F-INCL), as well as male infertility of all causes (M-ALL) in seven cohorts, primarily of European ancestry (EUR) (Fig. 1 and Supplementary Tables 1 and 2). The case–control ratio of all-cause female infertility ranged from 0.9% in the deCODE Genetics dataset to 11.7% in FinnGen, whereas the case–control ratio of male infertility was between 0.3% (UK Biobank (UKBB)) and 8.2% (Danish Biobank) (Fig. 1 and Supplementary Table 2). Anatomical female infertility was the least common cause of infertility in three of six cohorts (prevalence in UKBB of 0.01%, FinnGen of 0.8% and Estonian Biobank (EstBB) of 2.0%). Owing to varying sample ascertainment, the case–control ratio does not necessarily reflect the population prevalence of infertility.

### Novel genetic loci for infertility

**Fig. 2:**
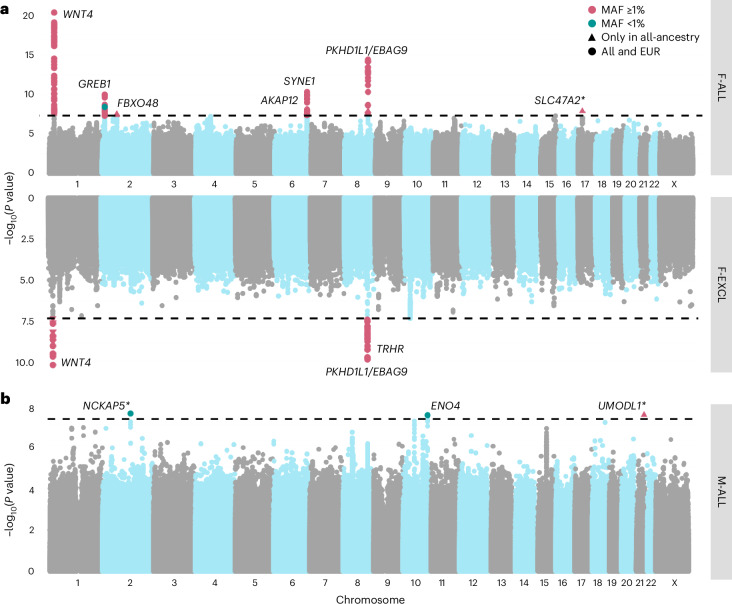
Miami and Manhattan plots for selected infertility meta-analyses. **a**, Genetic variants associated with F-ALL (top) and idiopathic infertility (unknown causes) defined by exclusion of known causes such as anatomical or anovulatory causes, PCOS, endometriosis and uterine leiomyomas (bottom). **b**, Genetic variants associated with M-ALL. Each point depicts a single SNP. The triangles represent SNPs that only reach genome-wide significance in all-ancestry GWAS meta-analyses. SNPs are annotated with the mapped gene. *The lead variant is reported in only one cohort. Summary statistics from whole-genome regression analyses were meta-analyzed using fixed-effect inverse-variance weighting in the METAL software to produce the displayed *P* values. The dashed line represents the multiple testing-corrected *P* value threshold of *P* < 5 × 10^−8^, accounting for ~1 million independent variants in the genome.

We performed GWAS meta-analyses, testing up to 33 million genetic variants for associations with each of the above categories of infertility, in up to 42,629 cases and 740,619 controls in women, and 10,886 cases and 995,982 controls in men (Fig. 1 and Supplementary Table 2). We identified 22 unique genome-wide significant (*P* < 5 × 10^−8^) loci associated with at least one category of female infertility and three loci for male infertility (minor allele frequency (MAF) range 0.06–46%) (Fig. 2, Table 1 and Supplementary Fig. 1). Fourteen loci (63.6%) for female infertility reached nominal significance (*P* < 2.27 × 10^−3^, Bonferroni correction for 22 independent loci tested) in at least one other infertility category (Supplementary Note and Supplementary Fig. 19). There was no evidence for heterogeneity in lead variant effects across cohorts (Supplementary Note and Supplementary Table 3).

**Table 1:** Lead variants associated with infertility on GWAS meta-analyses

rsID	chr:pos:A1:A2 (hg38)	Mapped gene	All ancestries			EUR only		
			Average MAF	OR (95% CI)	*P* value	Average MAF	OR (95% CI)	*P* value
**Female infertility of all causes (F-ALL)**								
rs61768001	chr1:22139327:C:T	*WNT4*	0.165	1.10 (1.08–1.12)	4.27 × 10^−21^	0.163	1.10 (1.08–1.12)	1.24 × 10^−19^
rs10929759	chr2:11581886:G:C	*GREB1*	0.456	0.952 (0.938–0.966)	1.03 × 10^−10^	0.456	0.951 (0.937–0.966)	8.03 × 10^−11^
rs75045132	chr2:68456386:C:G	*FBXO48*	0.0331	0.888 (0.851–0.926)	3.20 × 10^−8^	0.0328	0.889 (0.852–0.929)	1.20 × 10^−7^
rs6938404	chr6:151222906:T:C	*AKAP12*	0.452	0.958 (0.944–0.973)	4.01 × 10^−8^	0.453	0.958 (0.943–0.973)	3.88 × 10^−8^
rs17803970	chr6:152232583:T:A	*SYNE1*	0.0833	0.914 (0.889–0.939)	5.06 × 10^−11^	0.0836	0.909 (0.885–0.934)	7.50 × 10^−11^
rs9643050	chr8:109458129:C:T	*PKHD1L1*	0.0601	1.13 (1.10–1.16)	3.98 × 10^−15^	0.0597	1.13 (1.09–1.16)	7.43 × 10^−14^
rs111749498*	chr17:19714694:T:C	*SLC47A2*	0.0273	2.29 (1.72–3.04)	1.29 × 10^−8^	–	–	–
**Anatomical female infertility (F-ANAT)**								
rs340879	chr1:213983171:T:C	*PROX1*	0.418	0.906 (0.874–0.939)	4.95 × 10^−8^	0.418	0.902 (0.869–0.936)	5.06 × 10^−8^
**Anovulatory female infertility (F-ANOV)**								
rs72665317	chr1:22040580:G:T	*CDC42*	0.190	1.14 (1.10–1.19)	7.76 × 10^−10^	0.180	1.13 (1.08–1.18)	1.45 × 10^−7^
rs72827480	chr2:120388925:C:T	*INHBB*	0.401	1.10 (1.07–1.14)	4.20 × 10^−8^	0.401	1.10 (1.07–1.14)	4.20 × 10^−8^
rs1852684	chr2:145068818:T:G	*ZEB2*	0.367	1.12 (1.08–1.16)	9.25 × 10^−10^	0.350	1.12 (1.08–1.17)	3.44 × 10^−10^
rs552953683	chr8:102898586:C:T	*AZIN1*	0.0024	2.93 (2.01–4.27)	2.54 × 10^−8^	0.0024	2.93 (2.01–4.27)	2.54 × 10^−8^
rs9696009	chr9:123856954:A:G	*DENND1A*	0.0777	1.21 (1.14–1.29)	6.87 × 10^−10^	0.0695	1.24 (1.16–1.32)	2.40 × 10^−10^
rs9902027	chr17:7537667:C:T	*TNFSF12*	0.255	1.12 (1.07–1.16)	4.06 × 10^−8^	0.255	1.12 (1.07–1.16)	4.06 × 10^−8^
rs143459581	chr22:28068862:T:C	*TTC28*	0.0419	1.30 (1.19–1.43)	1.21 × 10^−8^	0.0419	1.30 (1.19–1.43)	1.21 × 10^−8^
rs17879961	chr22:28725099:G:A	*CHEK2*	0.0389	1.35 (1.23–1.48)	1.55 × 10^−10^	0.0389	1.35 (1.23–1.48)	1.55 × 10^−10^
**Idiopathic female infertility, exclusion definition (F-EXCL)**								
rs61768001	chr1:22139327:C:T	*WNT4*	0.165	1.08 (1.06–1.11)	7.49 × 10^−11^	0.162	1.08 (1.05–1.10)	2.48 × 10^−9^
rs111597692	chr8:109039973:T:C	*TRHR*	0.0323	1.16 (1.10–1.22)	1.51 × 10^−8^	0.0323	1.16 (1.1–1.22)	1.51 × 10^−8^
rs17378154	chr8:109568721:A:G	*PKHD1L1*	0.0590	1.13 (1.09–1.17)	1.64 × 10^−10^	0.0593	1.13 (1.09–1.17)	3.36 × 10^−10^
**Idiopathic female infertility, inclusion definition (F-INCL)**								
rs61768001	chr1:22139327:C:T	*WNT4*	0.170	1.15 (1.11–1.19)	6.87 × 10^−14^	0.165	1.15 (1.10–1.19)	8.96 × 10^−13^
rs11692588	chr2:11544358:A:G	*GREB1*	0.358	0.919 (0.892–0.947)	2.98 × 10^−8^	0.358	0.919 (0.892–0.947)	2.98 × 10^−8^
rs190290095	chr4:39786858:A:G	*UBE2K*	0.0022	0.227 (0.137–0.375)	7.60 × 10^−9^	0.0022	0.227 (0.137–0.375)	7.60 × 10^−9^
rs851982	chr6:151703850:C:T	*ESR1*	0.428	1.08 (1.06–1.12)	7.60 × 10^−9^	0.437	1.08 (1.05–1.12)	2.86 × 10^−8^
rs17378154	chr8:109568721:A:G	*PKHD1L1*	0.0565	1.18 (1.11–1.25)	2.47 × 10^−8^	0.0569	1.18 (1.11–1.25)	4.97 × 10^−8^
rs74156208	chr10:61509370:A:G	*TMEM26*	0.184	1.10 (1.06–1.14)	4.96 × 10^−8^	0.187	1.10 (1.07–1.15)	5.44 × 10^−8^
rs192462512*	chrX:39653668:C:T	*BCOR*	0.0015	0.227 (0.142–0.363)	5.26 × 10^−10^	0.0015	0.227 (0.142–0.363)	5.26 × 10^−10^
**Male infertility of all causes (M-ALL)**								
rs1228269928*	chr2:132923776:T:A	*NCKAP5*	0.0006	10.0 (4.45–22.7)	2.72 × 10^−8^	0.0006	10.0 (4.45–22.7)	2.72 × 10^−8^
rs139862664	chr10:116879589:G:C	*ENO4*	0.0072	2.58 (1.84–3.60)	3.29 × 10^−8^	0.0072	2.58 (1.84–3.60)	3.29 × 10^−8^
rs75957543*	chr21:42081234:G:C	*UMODL1*	0.0125	1.67 (1.39–2.01)	3.19 × 10^−8^	–	–	–

Summary statistics from whole-genome regression analyses were meta-analyzed using fixed-effect inverse-variance weighting in the METAL software. Lead variants were identified by distance-based pruning within windows of 1 Mb at all loci with at least one variant with *P* < 5 × 10^−8^ (multiple testing correction for ~1 million independent variants in the genome). A1 is the minor (effect) allele. Exonic or intronic variants in coding genes are mapped to their genes; intergenic variants are mapped to the nearest coding gene (by TSS). *Lead variant is reported in only one cohort. Blank cells indicate that the variant was not present in the EUR-only meta-analysis.

Among the variants associated with multiple subtypes of female infertility is rs9643050 (MAF of 6.01%), an intronic variant in *PKHD1L1* (F-ALL, odds ratio (OR) (95% confidence interval (CI)) 1.13 (1.09–1.16); F-EXCL, OR 1.13 (1.09–1.17); F-INCL, OR 1.18 (1.11–1.25)). This variant is 76 kb upstream of *EBAG9*, an estrogen-responsive gene previously reported to have a recessive association with female infertility^12,13^ and thought to suppress maternal immune response during pregnancy^14,15^. We also identified an intronic variant in *WNT4*, rs61768001 (MAF of 16.5%), associated with three categories of female infertility (F-ALL, OR 1.10 (1.08–1.12); F-EXCL, OR 1.08 (1.06–1.11); F-INCL, OR 1.15 (1.11–1.19)). *WNT4* is highly pleiotropic for female reproductive traits, as it is reported to associate with gestational length^16^, uterine fibroids^17,18^, endometriosis^19,20^, female genital prolapse^21^ and bilateral oophorectomy^21^. Such pleiotropy reflects the role of *WNT4* as a key regulator of female reproductive organ development during embryogenesis^22^.

The nearest gene to the idiopathic infertility-associated variant rs111597692 (MAF of 3.23%; F-EXCL, OR 1.16 (1.10–1.22)) is *TRHR*, which encodes the thyrotropin-releasing hormone receptor. Mice with *Trhr* knockout display a phenotype similar to primary ovarian insufficiency^23^. The F-ANOV-associated variant rs72827480 (MAF of 40.1%, OR 1.10 (1.07–1.14)) colocalizes with a testis expression quantitative trait locus (eQTL) for *INHBB* in the GTEx Project (posterior probability (PP) of shared causal variant of 91.6%; Supplementary Table 4). *INHBB* encodes the beta subunit of inhibin B, which regulates hypothalamic, pituitary and gonadal hormone secretion^24^, and ovarian follicle and oocyte development^25^. rs111749498 (MAF of 2.73%, associated with F-ALL, OR 2.29 (1.72–3.04)) is near *SLC47A2*, which encodes a multidrug efflux pump that mediates excretion of the drug metformin, commonly used to manage infertility in women with PCOS^26^. Variants associated with all-cause female infertility are in genes enriched for expression in ovarian stromal cells (partitioned heritability *P* = 2.52 × 10^−3^; Supplementary Note).

The male infertility-associated variant rs75957543 (MAF of 1.25%, OR 1.67 (1.39–2.01)) is near *UMODL1*, which encodes the olfactorin protein, expressed along the migratory route of gonadotropin-releasing hormone neurons. Impairment of gonadotropin-releasing hormone migration is a feature of Kallmann’s syndrome, the most common genetic cause of hypogonadotropic infertility^27^. While mutations in *UMODL1* have been shown to impact ovarian follicle development, granulosa cell apoptosis and female fertility in model organisms^28,29^, its role in male infertility remains unclear. Finally, an intronic variant in *ENO4*, which is expressed in the testis and may play a role in sperm motility^30^, is associated with male infertility (rs139862664, MAF of 0.72%, OR 2.58 (1.84–3.60)). Male mice with *Eno4* knockout display infertility, abnormal sperm morphology and physiology and decreased testis weight, among other altered male reproductive tract phenotypes^31^.

### Relationships with other female reproductive conditions

**Fig. 3:**
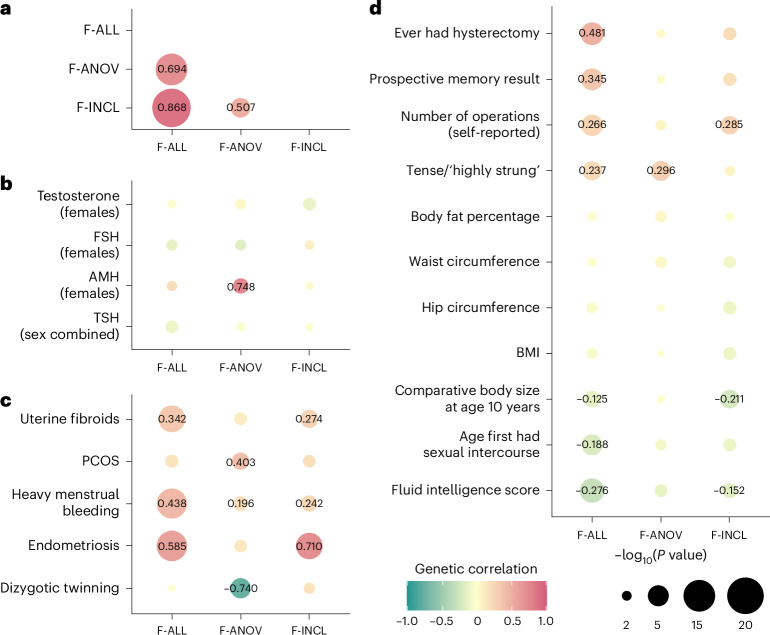
Genetic correlations between female infertility and other phenotypes. SNP-based genetic correlations (*r*_g_) between significantly heritable phenotypes (*Z* > 4) were estimated using LD-score regression, performed using the LDSC software on a subset of 1 million HapMap3 SNPs. The points are colored by *r*_g_ estimate, scaled by significance (−log_10_(*P*)), and labeled with the associated *r*_g_ estimate if nominally significant without correction for multiple testing (*P* < 0.05). **a**, Genetic correlations among three definitions of female infertility (F-ALL, F-ANOV and F-INCL). **b**, Genetic correlations between female infertility traits and reproductive hormones testosterone, FSH and AMH (publicly available summary statistics) in female-specific analyses and TSH (publicly available summary statistics) from sex-combined analysis. **c**, Genetic correlations between female infertility traits and female reproductive conditions, with summary statistics generated from the largest available EUR-ancestry studies for each trait (Methods). **d**, Genetic correlations between female infertility traits and selected heritable phenotypes (*Z* > 4) in the UKBB, as generated by the Neale laboratory. Correlations with all heritable phenotypes can be found in Supplementary Table 12.

**Fig. 4:**
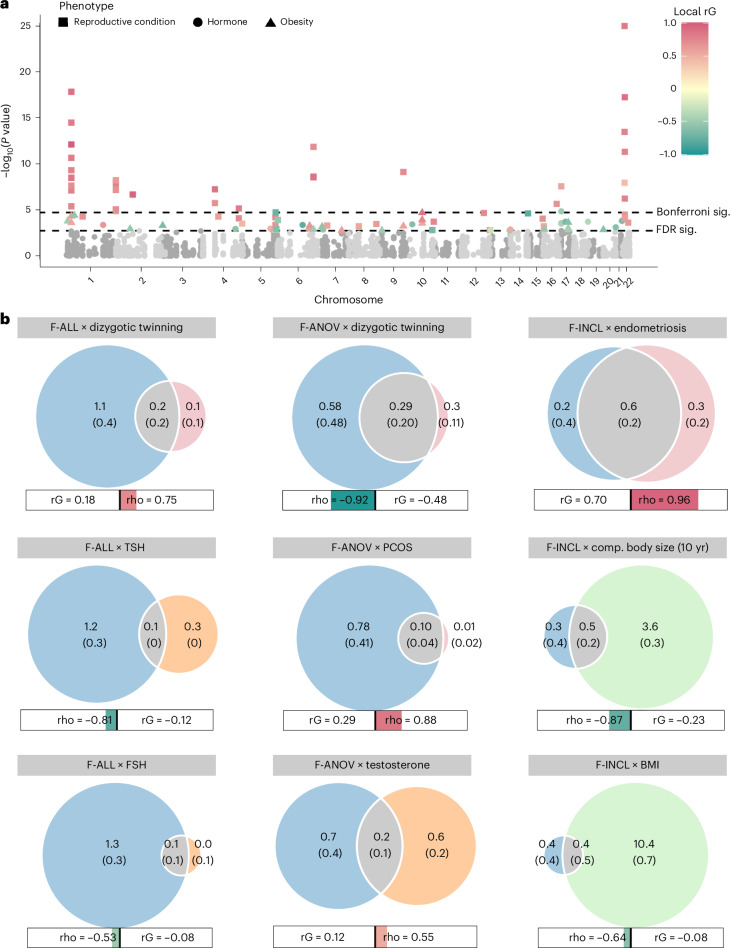
Local genetic correlations and polygenic overlap between female infertility and other phenotypes. **a**, Local genetic correlations, estimated using LAVA, at 2,495 blocks across the genome. Each point represents a local bivariate genetic correlation between an infertility trait (F-ALL, F-ANOV or F-INCL) and reproductive hormone, reproductive condition or obesity-related trait. The dashed lines indicate significance (sig.) thresholds. The dashed line represents FDR-adjusted or Bonferroni-adjusted *P* values of 0.05. **b**, MiXeR estimates of polygenic overlap. The Venn diagrams indicate the estimated number (s.e.m.) of causal variants (in thousands) that explain 90% SNP heritability per component. The circle size reflects the degree of polygenicity. The bars outline the genome-wide genetic correlation (rG) and correlation in the shared polygenic component (rho). The colored portion of the bar is sized by the proportion of causal variants in the shared polygenic component as compared with all causal variants involved and colored by rho. Comp., comparative.

Genome wide, we observed positive genetic correlations (Fig. 3a) between endometriosis and F-ALL (*r*_g_ (s.e.m.) = 0.585 (0.0785), *P* = 8.98 × 10^−14^) and F-INCL (*r*_g_ = 0.710 (0.115), *P* = 5.94 × 10^−10^). We also observed positive correlation between F-ANOV and PCOS, the most common cause of anovulatory infertility (*r*_g_ = 0.403 (0.131), *P* = 2.20 × 10^−3^). We tested for local bivariate genetic correlations between infertility and PCOS, endometriosis, heavy menstrual bleeding and uterine fibroids at 2,495 blocks across the genome, chosen to be approximately 1 Mb in length each, while minimizing linkage disequilibrium (LD) between blocks. Consistent with the genome-wide *r*_g_, we found positive local *r*_g_ between female infertility and reproductive disorders at 11 regions (*P* < 1.91 × 10^−5^, Bonferroni adjustment for 2,618 local bivariate tests performed at regions with significant heritability of both traits in each pair tested; Fig. 4a and Supplementary Table 22). At 5/11 blocks, infertility was correlated with more than one reproductive condition, none of which had individual effects after conditioning upon the other associated reproductive disorders in the region (all *P* > 0.05; Supplementary Table 22).

Furthermore, we used MiXeR^32^ to assess bivariate polygenic overlap, regardless of genome-wide genetic correlation, between infertility and reproductive conditions. We found that approximately 50% of causal single-nucleotide polymorphisms (SNPs) involved in endometriosis, and about 25% of causal SNPs involved in uterine fibroids were shared with the assessed infertility phenotypes, with varying degrees of genetic correlation in the shared component (Fig. 4b, Supplementary Table 24 and Supplementary Note). We noted that while there was substantial correlation in the shared component of F-ANOV and PCOS (rho (s.e.m.) of 0.878 (0.242)), only 97 (10.9%) of the 888 causal variants involved were shared; the majority (88.2%) of variants were unique to F-ANOV and only 8 variants (<1%) were unique to PCOS, suggesting that a small proportion of causal variants drive the genetic correlation between these traits (Fig. 4b and Supplementary Table 24).

We observed genome-wide negative correlation between F-ANOV and spontaneous dizygotic twinning, a heritable metric of female fecundity that captures the propensity for multiple ovulation^33^ (*r*_g_ = −0.740 (0.182), *P* = 4.93 × 10^−5^). We also found substantial negative correlation in the shared polygenic component of these traits (rho (s.e.m.) = −0.920 (0.129)), with 32% (295) shared SNPs of the 912 total causal SNPs involved (Fig. 4b, Supplementary Table 24 and Supplementary Note).

Two loci associated with both endometriosis and female infertility (*WNT4* and *ESR1*) may share the same putative causal variant (PP >93.6%; Supplementary Table 5). Variants in both these genes have previously been associated with endometriosis-related infertility^34,35^. *GREB1* and *SYNE1* also contain overlapping signals for infertility and endometriosis, but there is strong evidence against shared causal variants (PP >75%; Supplementary Table 5). Finally, three of eight loci for anovulatory infertility (*INHBB*, *TTC28* and *CHEK2*) may share a causal variant with PCOS (PP >89.2%; Supplementary Table 5).

### Evolutionary persistence of infertility-associated variants

The genome-wide SNP heritability estimates (on the liability scale, accounting for disease prevalence) for all categories of infertility were <10% (lowest for M-ALL at 1.12% (s.e.m. 0.93) and highest for F-ANOV at 9.54% (s.e.m. 2.16); Supplementary Table 6). This is lower than heritability estimates of two-thirds of all heritable binary phenotypes in the UKBB, with population prevalence similar to that of infertility (64 phenotypes with *Z* > 4 and prevalence <5%)^36^. We hypothesized that infertility risk-increasing alleles are subject to negative selection^37^, so we tested whether there was evidence for (1) variants associated with infertility in loci under historical or recent directional selection^38^ or (2) recent directional selection (over the past 2,000–3,000 years) measured by singleton density scores (SDSs)^39^ and balancing selection measured by standardized BetaScan2 scores^40^ at infertility loci (Supplementary Note).

**Fig. 5:**
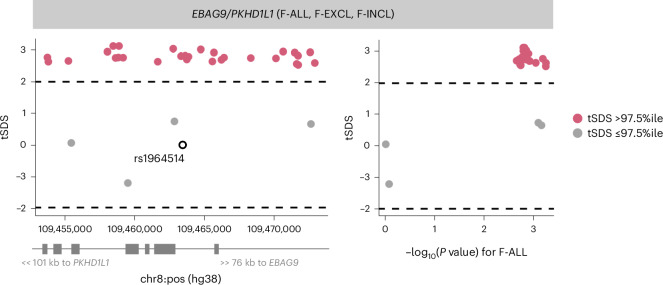
Directional selection scores at infertility-associated *EBAG9* locus. Recent directional selection, as measured by trait-aligned SDSs (tSDSs) at the *EBAG9* locus. The window of ±10 kb around the lead variant associated with F-ALL is displayed, along with the location of nearest gene TSSs. The tSDSs are aligned to the infertility risk-increasing allele, wherein a positive tSDS indicates positive selection for infertility risk-increasing allele at the locus. The dashed lines indicate 2.5th percentile (%ile) and 97.5th %ile of SDSs. Left: a locus plot depicting genomic position on the *x* axis and tSDS on the *y* axis. The lead variant rs1964514 (open circle) is not present in the tSDS dataset and thus is assigned a score of 0. Right: a scatter plot depicting relationship between −log_10_ of the GWAS *P* value for the variant association with F-ALL on the *x* axis and tSDS on the *y* axis.

While we found no genome-wide signature of directional selection against infertility (Supplementary Note), we observed extreme SDSs (in the highest 99.75th percentile of SNPs within 10 kb of a GWAS catalog variant) at the *EBAG9* locus associated with female infertility, indicating recent positive selection (Fig. 5 and Supplementary Table 7).

### Genetic determinants of reproductive hormone levels

#### Identification of novel reproductive hormone loci

As hormone dysregulation is central to many infertility diagnoses^5,6^, we conducted sex-specific GWAS meta-analyses of five reproductive hormones—follicle-stimulating hormone (FSH) (*n*_female_ = 57,890, *n*_male_ = 6,095), luteinizing hormone (LH) (*n*_female_ = 47,986, *n*_male_ = 6,769), estradiol (*n*_female_ = 97,887, *n*_male_ = 39,165), progesterone (*n*_female_ = 18,368) and total testosterone (*n*_female_ = 246,862, *n*_male_ = 243,951)—collected at assessment center visits or identified through electronic health records, in six cohorts and publicly available summary statistics (Supplementary Table 9). We identified genome-wide significant loci associated with FSH (9 novel/2 previously known in females and 0/1 in males), LH (4/2 in females and 1/0 in males), estradiol (1/1 in females and 3/4 in males) and testosterone (39/118 in females and 67/206 in males), but found no genetic variants associated with progesterone (Supplementary Figs. 3, 4 and 20). Several of the reported signals we replicated are near genes encoding the hormone-specific subunits themselves, such as *FSHB* for FSH and *LHB* for LH, or enzymes for steroid-hormone metabolism, such as *CYP3A7* for estradiol and *HSD17B13* for testosterone (Supplementary Note).

Among the novel variants for testosterone in men were those near *SPOCK1* (rs1073917: MAF of 30.7%, *β* (s.e.m.) = 0.0160 (0.0029), *P* = 4.69 × 10^−8^), which is a target for the androgen receptor^41^, and *NR4A3* (rs10988865: MAF of 27.4%, *β* = 0.0161 (0.0029), *P* = 4.33 × 10^−8^), which coordinates the cellular response to corticotropin hormone- and thyrotropin hormone-releasing stimuli^42^ (Supplementary Table 10). Novel reproductive hormone variants associated with testosterone in women include those near *LAMTOR4* (rs17250196: MAF of 5.13%, *β* = −0.131 (0.0067), *P* = 4.02 × 10^−86^), associated with hyperthyroidism^23^ and age at menarche and menopause^43^, and obesity-associated *CCDC146* (rs138240474: MAF of 0.63%, β = −0.116 (0.0207), *P* = 2.03 × 10^−8^)^44^, which is expressed in the fallopian tubes and endometrium^45^.

Clinical measurements of FSH and LH may be used to diagnose premature menopause^46^, but our hormone GWASs based on these measurements were robust to this potential ascertainment bias (Supplementary Note). They were also robust to the inclusion of summary statistics from publicly available datasets and there was no evidence for heterogeneity in variant effects across cohorts (Supplementary Note).

### Relationships with other heritable phenotypes

We observed no genome-wide genetic correlations between any category of female infertility and (1) any reproductive hormone in this study, (2) thyroid stimulating hormone (TSH) or (3) anti-Mullerian hormone (AMH), the latter two based on publicly available summary statistics^47,48^ (all *P* > 0.05, except the correlation between AMH and F-ANOV, *r*_g_ (s.e.m.) = 0.748 (0.301), *P* = 0.0131; Fig. 3b). Consistent with the genome-wide results, we also found no evidence for local genetic correlations between any category of infertility and the above hormones (all *P* > 1.91 × 10^−5^; Fig. 4a, Supplementary Table 22 and Supplementary Note). The limited genetic correlation between infertility and reproductive hormones was mirrored in polygenic overlap analyses. The highest proportion of shared SNPs between these traits was 14.5% between F-ANOV and testosterone (209/1,444 variants shared, rho (s.e.m.) of 0.549 (0.252) in the shared polygenic component), followed by 14.0% between F-ANOV and AMH (123/881, rho (s.e.m.) of 0.993 (0.0301); Fig. 4b and Supplementary Table 24).

Mendelian randomization (MR) analyses indicated a genetically causal protective effect of FSH on risk of F-ALL (OR (95% CI) 0.776 (0.678–0.888), *P* = 2.15 × 10^−4^) and F-EXCL (0.716 (0.604–0.850), *P* = 1.26 × 10^−4^) (Supplementary Table 11). We found evidence for shared variants between hormones and infertility at the *FSHB* locus associated with FSH, LH and testosterone (PP >84.8% for colocalization with F-ANOV), and the *ARL14EP* locus associated with LH (PP 89.3% for colocalization with F-ANOV) (Supplementary Table 12). There was no evidence for colocalization at any of the >300 other genome-wide significant loci associated with infertility or reproductive hormones in our study (Supplementary Table 12).

Across 702 heritable phenotypes in the UKBB, we found 15 traits to be genetically correlated with female infertility, which we broadly group into: female reproductive conditions (such as having had a hysterectomy, *r*_g_ (s.e.m.) = 0.481 (0.0963)), general illness (such as number of operations, *r*_g_ = 0.266 (0.0588)), and cognitive test results (overall prospective memory test *r*_g_ = 0.345 (0.0736) and overall fluid intelligence *r*_g_ = −0.276 (0.0502)) (Fig. 3d and Supplementary Table 13). We found that 24 obesity-related traits, including body mass index (BMI), waist-to-hip ratio (WHR) and body fat percentage, were correlated with testosterone and FSH, but not with any category of female infertility (all *P* > 0.05; Fig. 3d, Supplementary Fig. 7 and Supplementary Table 13).

We found no evidence for local genetic correlations between any category of infertility and five obesity-related traits at 2,495 regions across the genome at a Bonferroni-adjusted significance threshold (all *P* > 1.91 × 10^−5^; Fig. 4a, Supplementary Table 22 and Supplementary Note). Polygenic analyses also revealed only limited overlap between infertility and obesity: fewer than 10% of causal SNPs involved were shared between infertility and any of the five obesity-related traits assessed (Fig. 4b, Supplementary Table 24 and Supplementary Note). The low overlap may reflect the polygenicity of obesity (between 4,050 and 11,000 causal variants), of which the majority (between 73.2% and 93.0%) are not involved in infertility (Supplementary Tables 23 and 24). Despite limited overlap, there was substantial negative correlation in the shared genetic components between F-INCL and comparative body size at age 10 years (451 shared SNPs of 4,385 involved, rho (s.e.m.) of −0.874 (0.143)) and adult BMI (393/11,185, rho (s.e.m.) of −0.640 (0.262)) (Fig. 4b and Supplementary Table 24).

Finally, MR analyses using genetic instruments for BMI, WHR and WHR adjusted for BMI (WHRadjBMI) indicated evidence for bidirectional causal relationships between infertility and abdominal obesity, independent of overall obesity. While genetically predicted WHRadjBMI was a risk factor for F-ALL (OR (95% CI) 1.10 (1.05–1.16), *P* = 1.71 × 10^−4^) and F-ANOV (OR 1.29 (1.16–1.45), *P* = 4.66 × 10^−6^), the latter was itself inferred to be causal for increased WHRadjBMI (*β* (s.e.m.) = 0.0547 (0.0133), *P* = 3.74 × 10^−5^) (Supplementary Table 11).

### Rare-variant contribution to infertility and hormone levels

We analyzed the 450k UKBB exome-sequencing dataset to characterize the association between rare coding variation (MAF <1%) and binary traits with >100 cases (F-ALL (3,746 cases, 260,413 controls), F-EXCL (3,012 cases, 261,147 controls) and M-ALL (650 cases, 222,393 controls)), and quantitative traits with >10,000 participants (FSH-F (*n* = 20,800), LH-F (*n* = 16,391), estradiol-F (*n* = 54,609) and testosterone (*n*_female_ = 197,038, *n*_male_ = 197,340)) (Fig. 1). Gene-burden analyses implicate the *PLEKHG4* gene, which is highly expressed in the testis and ovary, for F-EXCL (burden test OR (95% CI) 1.04 (1.02–1.06) when aggregated across all variant annotations with MAF <1%, Cauchy *P* = 2.47 × 10^−7^) (Supplementary Table 14). This association did not replicate in the deCODE or Genes & Health (G&H) datasets (*P* > 0.05; Supplementary Tables 14, 20 and 21).

#### Novel genes implicated by gene-burden analyses

Gene-based analyses identify 18 genes associated with testosterone-F and 20 genes with testosterone-M (Cauchy combination *P* < 5 × 10^−6^), of which ten have not previously been implicated through GWASs (Supplementary Note). Across 43 gene–trait pairs with Cauchy *P* < 5 × 10^−6^ in UKBB, 13 (30.2%) replicate at nominal significance (*P* < 0.05) and two (4.65%) at Bonferroni-adjusted significance (*P* < 6.85 × 10^−4^) in either the deCODE or G&H datasets with consistent directions of effect (Supplementary Tables 14, 20 and 21).

We replicated the testosterone-F lowering associations of rare damaging variation in the hydroxysteroid dehydrogenase enzymes *AKR1D1* (UKBB *P* = 1.76 × 10^−8^, deCODE *P* = 1.08 × 10^−7^, G&H *P* = 0.862) and *AKR1C3* (UKBB *P* = 2.21 × 10^−9^, deCODE *P* = 1.12 × 10^−6^, G&H *P* = 8.75 × 10^−8^) in external cohorts (*P* < 6.85 × 10^−4^, Bonferroni adjustment for 43 independent gene–trait pairs) (Supplementary Tables 14, 20 and 21). We report the first known association of *HSD11B1* with testosterone-F (burden test *P* = 1.93 × 10^−6^ when aggregated across missense variants with MAF <0.01%), with nominal replication in deCODE (*P* = 0.028); pathogenic variants in this gene are reported to cause hyperandrogenism due to cortisone reductase deficiency^49^ (Supplementary Fig. 11). We also report the association of testosterone-M with *HSD17B2* (burden test *P* = 1.33 × 10^−11^ when aggregated across predicted loss-of-function (pLoF) variants with MAF <0.1%), which encodes the enzyme hydroxysteroid 17β-dehydrogenase 2 that regulates the biological potency of steroid hormones^50^ (Supplementary Fig. 11 and Supplementary Table 14). The association of rare damaging variation in *HSD17B2* with lower testosterone nominally replicated in deCODE (*P* = 2.22 × 10^−3^) and G&H (*P* = 0.0481).

#### Infertility risk linked to rare hormone-associated variants

Two genes associated with testosterone in female UKBB participants were also associated with infertility risk (*P* < 1.00 × 10^−3^, Bonferroni adjustment for 50 unique genes): *TRIM4* (F-ALL, burden test OR 1.03 (1.01–1.05), *P* = 4.05 × 10^−4^ across all variants with MAF <0.1%) and *CYP3A43* (F-EXCL, burden test OR 1.02 (1.01–1.03), *P* = 4.84 × 10^−4^ across all variants with MAF <1%). Neither gene has previously been implicated in infertility.

**Fig. 6:**
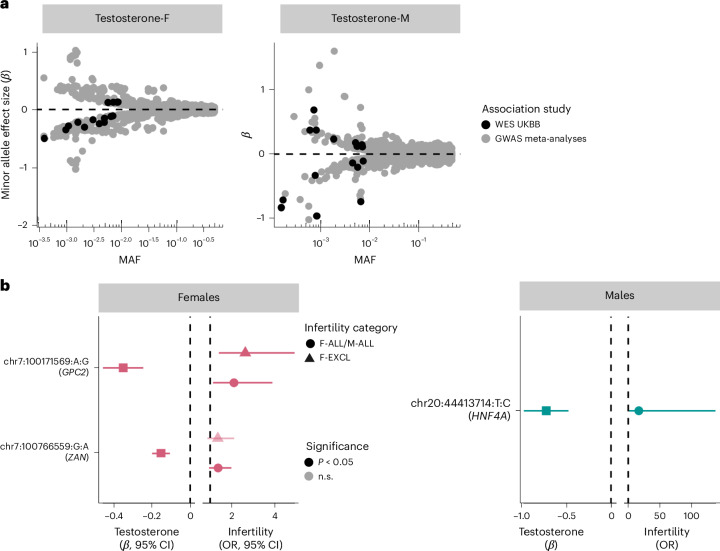
Rare variants associated with testosterone and infertility in UKBB WES analyses. **a**, The mean effect size versus allele frequency of genetic variants associated with total testosterone estimated using regression analyses. Variants discovered at genome-wide significance (*P* < 5 × 10^−8^) in GWAS meta-analyses (*n*_female_ = 235,579, *n*_male_ = 235,096) and exome-wide significance (*P* < 1 × 10^−7^) in the UKBB WES analyses (*n*_female_ = 197,038, *n*_male_ = 197,340) are plotted. The effect sizes are aligned to the minor allele, plotted against MAF on the log *x* axis. **b**, The effects of testosterone-associated rare variants (chr:pos:minor allele:major allele) on infertility in females (left: *n* cases/controls for F-ALL = 3,746/260,413; *n* cases/controls for F-EXCL = 3,012/261,147) and males (right: *n* cases/controls for M-ALL = 650/222,393) estimated using regression analyses. The effect sizes are aligned to the minor allele. Per gene, the variant with the lowest *P* value of all variants that reach exome-wide significance in UKBB WES analyses for testosterone is displayed, for all variants with nominally significant effects on infertility. Effect sizes (*β* and 95% CIs) for the variant effect on testosterone are to the left of each plot and effect sizes (ORs and 95% CIs) for the variant effect on infertility are to the right of each plot.

Finally, we identified 29 unique genes carrying rare variants (MAF <1%) associated with testosterone in male or female participants in the UKBB, a majority of which had the same direction of effect in our EUR-ancestry GWAS meta-analyses excluding UKBB participants (Supplementary Table 15 and Supplementary Note). Nineteen of the 29 genes also contained nearby (±500 kb) common testosterone-associated variants from GWASs (MAF >1%), but at the majority (74%) of these loci, the effect of the rare variant was larger and remained upon conditioning on common variants (*P* < 1 × 10^−7^ after conditioning; Fig. 6a, Supplementary Table 15 and Supplementary Note).

The 11 novel testosterone associations included a female testosterone-lowering missense variant in *STAG3* (chr7:100204708:C:T, *β* = −0.284, *P* = 2.31 × 10^−8^). *STAG3* is also associated with primary ovarian insufficiency in women^51^, and lack of *Stag3* results in female infertility through the absence of oocytes in knockout mouse models^23^. We did not find a significant association between the *STAG3* variant and female infertility in UKBB (*P* > 0.05). However, we observed increased risk of idiopathic infertility in women carrying a novel testosterone-lowering variant in *GPC2* (chr7:100171569:G:A, F-EXCL OR 2.63 (1.40–4.92), *P* = 1.25 × 10^−3^) (Fig. 6b). *GPC2* is highly expressed in the testis, and *Gpc2-*knockout mouse models display reduced adrenal gland size^23^. The gene has not previously been reported to be associated with testosterone or infertility. Taken together, our results indicate a potential role for infertility driven by rare hormone-disrupting variants.

## Discussion

Our large-scale genetic investigation of infertility and related reproductive phenotypes in over 1.5 million individuals identified 22 genetic loci associated with female infertility, three with male infertility and novel variants associated with levels of the reproductive hormones FSH, LH, estradiol and total testosterone in men and women. Through rare-variant and gene-based analyses in the UKBB, we additionally identified 50 genes associated with testosterone levels, including the first reported hormone-associated variants in some members of the hydroxysteroid dehydrogenase enzyme family. Although there was evidence for distinct genetic architectures of infertility and reproductive hormones, we showed that individual genes containing rare protein-coding variants associated with testosterone (*GPC2*, *CYP3A43* and *TRIM4*) were also associated with higher risk of infertility in the UKBB.

Previous efforts to catalog the genome-wide architecture of infertility have relied on proxy measures such as childlessness and number of children ever born^10,11^, which may be confounded by behavioral, socioeconomic and lifestyle factors. While we found modest genetic correlation between female infertility and age at first sexual intercourse (−18.8%), indicating that the latter captures some shared biology with fertility, our meta-analyses prioritize novel genes with putative roles in male and female gonads, such as *TRHR* for ovarian insufficiency and *ENO4* for sperm motility, those responsible for development of the endocrine system, such as *UMODL1*, and pharmacogenetic interactions, such as the association of *SLC47A2* with female infertility, potentially mediated by response to metformin.

The strong genetic correlation of 71% between idiopathic infertility and endometriosis may indicate that some proportion of idiopathic cases are due to underdiagnosis of endometriosis, whose early treatment may prevent future infertility^52^. Our subtype-specific analyses highlight the value in dissecting heterogeneous causes of infertility. For example, PCOS is a heritable cause of up to 80% of anovulatory infertility cases that may be treated through induced ovulation^53^. However, only three of eight loci for anovulatory infertility colocalize with known PCOS signals, the genetic correlation between these traits is only 40% and the majority (88%) of causal variants for anovulatory infertility are not shared with PCOS. These results suggest that other hypothalamic–pituitary–ovarian disorders, endocrinopathies (hypothyroidism, hyperprolactinemia and so on) and ovarian insufficiency may also contribute significantly to the genetic etiology of anovulatory infertility, and require treatments different from those for PCOS-associated infertility^54^. Weight loss for overweight patients is often recommended as beneficial for fertility^55,56^, but we did not find substantial genetic correlation between obesity and infertility. Our findings add genetic support to evidence from randomized controlled trials demonstrating limited fertility benefits from short-term weight loss in overweight and obese women^57^. Instead, we observed bidirectional causal relationships between abdominal obesity and anovulatory infertility, suggesting physiological feedback mechanisms whose complex interplay requires deeper study.

Our results indicate that balancing selection and recent positive selection at pleiotropic loci may explain the persistence of genetic factors for infertility. For example, the *EBAG9* locus associated with female infertility is under directional selection, perhaps because *EBAG9* plays a role in the adaptive immune memory response to infection^58^. A complementary role for *EBAG9* may be in the placenta during early pregnancy, where reduction of *EBAG9* levels is associated with inappropriate activation of the maternal immune system and results in fetal rejection^15^.

We employed a broad search strategy to maximize sample sizes for cases of infertility and reproductive hormone levels in our meta-analyses, which has its limitations. Diagnostic criteria for infertility vary by country and have changed over time^1^, which may explain the wide spread in the prevalence of infertility across cohorts. Reproductive hormone values in this study were assayed using different methodologies and at different ages and stages of the menstrual cycle in women. A majority of samples in our study were derived from the UKBB and measured during and postmenopause (ages 40–69 years), whereas infertility occurs premenopause, so we urge caution in interpreting the lack of correlation between these traits. Although we were able to adjust for covariates such as age, which can account for some of the effect of menopause on hormone levels, we did not have the data granularity to account for hormonal fluctuations during the menstrual cycle and pregnancy.

In this comprehensive large-scale investigation of the genetic determinants of infertility and reproductive hormones across men and women, we identified several genes associated with infertility and analyzed their effects on reproductive disease and selection pressures. We did not find evidence that reproductive hormone dysregulation and obesity are strongly correlated with infertility at the population level, but instead nominate individual hormone-associated genes with effects on fertility. Other genetic and non-genetic avenues must be explored to treat complex and heterogeneous fertility disorders that impact the physical, emotional and financial well-being of millions of individuals across the globe.

## Methods

This research complies with all relevant ethical regulations. Each contributing study was approved by its respective board/committee as detailed in the Supplementary Note.

### Study populations and phenotype identification

#### Binary traits (infertility)

Cases were identified in UKBB, Copenhagen Hospital Biobank and Danish Blood Donor Study, deCODE, EstBB, FinnGen and G&H (Supplementary Methods). We defined five categories of female infertility: F-ALL, F-ANOV, F-ANAT (including tubal, uterine and cervical origins), F-EXCL and F-INCL, and male infertility of all causes (M-ALL). Cases were identified through self-report (F-ALL, F-EXCL and M-ALL) and through primary- and secondary-care codes (Supplementary Table 1). Within each subtype, sex-matched controls were defined as individuals not identified as cases for that subtype. We additionally included publicly available multi-ancestry summary statistics from the Million Veteran Program (MVP) in meta-analyses of F-ALL (PheCode 626.8) and M-ALL (PheCode 609), downloaded from dbGaP^59^.

#### Quantitative traits (reproductive hormones)

Hormones were included from UKBB, Avon Longitudinal Study of Parents and Children (ALSPAC), deCODE, EstBB and G&H (Supplementary Note). We extracted measurements of FSH, LH, estradiol, progesterone and testosterone from biobank assessment centers or primary- and secondary-care records (Supplementary Table 16). If repeated measurements were available for an individual, we retained the recorded hormone value closest to the individual’s median hormone value over time. Each hormone was regressed on age, age^2^ and cohort-specific covariates specified below; the residuals from this regression were rank-based inverse normally transformed before GWAS.

### Meta-analysis of GWAS summary statistics

#### Genome-wide association testing

Association analyses were performed separately within each ancestry and sex stratum for all strata with at least 100 cases (binary traits) or 1,000 individuals (quantitative traits). For binary traits, each variant passing quality control (QC) was tested for association under an additive model using REGENIE^60^ or Scalable and Accurate Implementation of GEneralized mixed model (SAIGE)^61^, with adjustments for age, age^2^ and cohort-specific covariates, with the Firth correction applied to control for inflation at rare variants and traits with low case–control ratios^60,61^. For quantitative traits, the rank-based inverse normally transformed hormone value was tested for association under an additive model using REGENIE^60^ or SAIGE^61^, with adjustments for cohort-specific genetic covariates. Any deviations from this GWAS protocol are noted in the Supplementary Note.

#### Meta-analysis

Before meta-analysis, summary statistics from all studies underwent thorough QC to retain variants that met the following criteria: (1) on the autosomes or X chromosome; (2) with imputation information score >0.8 (where available); (3) bi-allelic variants with A, C, G, T alleles; (4) with s.e.m. <10 and *P* values in (0, 1); and (5) without duplicate entries. Fixed-effects inverse-variance-weighted meta-analysis was performed using METAL^62^. We report results from EUR-ancestry and all-ancestry meta-analyses for each trait. Genome-wide significance was established at *P* < 5 × 10^−8^.

#### Identification of lead variants

Distance-based pruning was used to identify lead variants as the SNP with the lowest *P* value within each 1 Mb window at all loci with at least one genome-wide significant variant with *P* < 5 × 10^−8^. Lead hormone-associated variants were classified as novel or previously reported (Supplementary Note).

### SNP-based heritability

The following analyses, which rely on population-specific LD patterns, were restricted to EUR-ancestry summary statistics with precomputed LD scores based on EUR-ancestry individuals in the 1000 Genomes dataset^63^, restricted to HapMap3 SNPs^64^. We estimated the SNP-based heritability (*h*_G_^2^) of a trait from GWAS summary statistics using LD-score regression as implemented in the LDSC software^65^. For infertility traits, the observed-scale heritability (*h*_obs_^2^) was converted to liability-scale heritability (*h*_liab_^2^), which accounts for the disease prevalence in the sample (*k*) and population (*K*), under the assumption that sample prevalence equals the population prevalence^66^.

### Genetic correlations

#### Genome-wide genetic correlations

LDSC was used to estimate genetic correlations between infertility traits, hormone levels and a collection of other phenotypes in the UKBB in EUR-ancestry individuals. To simplify computation of *r*_g_ across a large number of traits, we used an extension of the LDSC software that allows for simultaneous estimation of multiple genetic correlations^67^ (Supplementary Note).

#### Local genetic correlations

We applied LAVA v1.8.0 to assess bivariate correlations and perform multivariate regressions at local genomic regions^68^, using 1000 Genomes reference genotype data. Genomic loci that were created by partitioning the genome into 2,495 blocks of approximately 1 Mb each, while minimizing LD between blocks, were downloaded from ref. ^69^. As we quantified sample overlap between traits using cross-trait LDSC, the phenotypes assessed were restricted to those with significant genome-wide heritability (*Z* > 4): female infertility (F-ALL, F-ANOV and F-INCL), five reproductive conditions (endometriosis, PCOS, heavy menstrual bleeding, uterine fibroids and dizygotic twinning), four hormones (FSH-F, testosterone-F, TSH and AMH) and five obesity-related traits (BMI, body fat percentage, waist circumference, hip circumference and comparative body size at age 10 years). The target phenotype (female infertility: F-ALL, F-ANOV or F-INCL) was tested against all other traits.

At each genomic block, we filtered to phenotypes with sufficient local heritability (*P* < 2.00 × 10^–5^, family-wise error rate (FWER) controlled at 5% across 2,495 regions using the Bonferroni method, as recommended by the developers of LAVA^68^), resulting in 2,618 pairwise bivariate *r*_g_ tests. At any regions where female infertility was correlated with multiple traits, we performed multiple regression to assess the independent predictive power of each trait. If traits were collinear in the region (local *r*_g_ > 0.9), the trait with the greatest local *r*_g_ with infertility was retained.

#### Polygenic overlap

We estimated polygenic overlap, irrespective of genetic correlation, using the causal mixture model MiXeR v1.3 (ref. ^32^) applied to GWAS summary statistics for female infertility (F-ALL, F-ANOV and F-INCL), five reproductive conditions (endometriosis, PCOS, heavy menstrual bleeding, uterine fibroids and dizygotic twinning), four reproductive hormones (FSH-F, testosterone-F, TSH and AMH) and five obesity-related traits (BMI, body fat percentage, waist circumference, hip circumference and comparative body size at age 10 years). For bivariate tests, the target phenotype (female infertility: F-ALL, F-ANOV or F-INCL) was tested against all other traits. GWAS summary statistics were formatted as for LDSC and LD structure was estimated using the 1000 Genomes project, as described previously^32^. Both univariate and bivariate models were fitted in two steps to ensure robust convergence, where the first ‘fast model’ estimates a set of parameters that are then constrained in the second ‘full model’. We only report estimates and s.e.m. for parameters from the ‘full model’, with the exception of Akaike information criterion (AIC) and Bayesian information criterion (BIC) from the first model, which are used to compare the univariate MiXeR model with LDSC regression, as recommended by the developers of MiXeR^32^. The number of causal variants is estimated from the fraction of total causal variants that cumulatively account for 90% of heritability in each component. Genome-wide genetic correlation estimates from MiXeR and LDSC were comparable as we used the same set of SNPs for both analyses; we calculated the *P* value for heterogeneity between these estimates using the *z* score for difference.

### MR

Analyses were all performed with summary statistics from EUR-ancestry GWASs, using the TwoSampleMR v0.5.7 package^70^. Details of instrument construction, sensitivity analyses and MR methods are in the Supplementary Note.

### Colocalization

The following analyses were all performed with summary statistics from EUR-ancestry GWASs, using the Bayesian framework implemented in the coloc v5.1.0 package^71^ under a single causal variant assumption^72^. Parameters for colocalization are provided in the Supplementary Note. We tested for colocalization between each female infertility category and each female-specific hormone trait (FSH, LH, estradiol and testosterone) at all genetic loci associated with at least one of the pair of traits tested. The single male infertility locus with common variants (MAF >1%) in the EUR-ancestry analysis did not contain enough significant associations (only 12 common variants with *P* < 1 × 10^−6^) for colocalization analyses. As we noticed that some lead variants for female infertility had previously been reported as associated with endometriosis and PCOS, we estimated the PP of colocalization of genetic signals between each category of female infertility and each of these two reproductive disorders. EUR-ancestry summary statistics for endometriosis and PCOS were obtained as described in ‘Genetic correlations’ section above. We assessed colocalization of genetic signals for female infertility with eQTLs for all proximal genes with transcription start sites (TSSs) within 1 Mb of an infertility lead variant. Publicly available eQTL data was downloaded from the GTEx project^73^.

### Tissue and cell-type prioritization

We estimated the polygenic contributions of genes with tissue-specific expression profiles to the heritability of infertility and hormones using stratified LD-score regression (partitioned heritability analyses)^65^. We restricted these analyses to traits with highly significant heritability in EUR-ancestry analyses (*Z* > 7) (F-ALL, testosterone-F and testosterone-M), as recommended by the developers^74^.

Gene sets and LD scores for 205 tissues and cell types from the GTEx project database^73^ and the Franke laboratory single-cell database^75^ were downloaded from ref. ^76^. We established tissue-wide significance at −log_10_(*P*) >2.75, which corresponds to a false discovery rate (FDR) <5%.

As the ovary, a reproductive tissue of interest, is not well characterized in the GTEx project, we constructed annotation-specific LD scores for ovarian cell types from two publicly available single-cell RNA sequencing datasets (Supplementary Note).

### Overlaps with genetic regions under selection

To avoid confounding by population stratification, selection look-ups were restricted to GWAS summary statistics from EUR-ancestry individuals. We assessed selection in three forms: (1) loci under directional selection, following guidelines described by Mathieson et al.^11^, (2) recent trait-wide directional selection using SDSs, following the protocol outlined by Field et al.^39^ and (3) balancing selection using standardized BetaScan2 scores^40^ (Supplementary Note).

### WES analyses

#### Exome sequencing QC

##### QC outline

We first considered an initial set of ‘high-quality’ variants to evaluate the mean call rate and depth of coverage for each sample. We then ran a sample and variant level prefiltering step and calculated sample-level QC metrics. Using these metrics, we removed sample outliers based on median absolute deviation thresholds and excluded sites that did not pass variant QC according to Karzcewski et al.^77^. We then applied a genotype-level filter using genotype quality, depth and heterozygote allele balance. The resultant high-quality EUR call set consisted of 402,375 samples and 25,229,669 variants. For details, see Supplementary Note.

#### Variant annotation

We annotated variants using Variant Effect Predictor (VEP) v105 (corresponding to gencode v39)^78^ with the LOFTEE v1.04_GRCh38 (ref. ^79^) and dbNSFP^80^ plugins, annotating variants with CADD v1.6 (ref. ^81^) and REVEL using dbNSFP4.3 (ref. ^82^) and loss of function confidence using LOFTEE. Complete instructions and code for this step are provided in our VEP_105_LOFTEE repository^83^, which contains a docker/singularity container to ensure reproducibility of annotations. We then ran SpliceAI v1.3 (ref. ^84^) using the gencode v39 gene annotation file to ensure alignment between VEP and SpliceAI transcript annotations. We defined ‘canonical’ transcripts to be used for variant-specific annotations as follows: set MANE Select^85^ as the canonical, where available, and if a MANE Select transcript is not present, set canonical and restrict to protein-coding genes. For VEP 105, this is equivalent to selecting the ‘canonical’ transcript in protein-coding genes. Then, using the collection of missense, pLoF, splice metrics and annotations of variant consequence on the ‘canonical’ transcript, we determined a set of variant categories for gene-based testing (Supplementary Note).

Variant counts and average allele counts for each annotation, split by population label and binned by MAF are displayed in Supplementary Figs. 13 and 14, respectively.

#### Genetic association testing

We carried out rare-variant genetic association testing in the EUR-ancestry subset of the UKBB using SAIGE^61^, a mixed model framework that accounts for sample relatedness and case–control imbalance through a saddle-point approximation in binary traits. All rare-variant analysis was carried out on the UKBB Research Analysis Platform using SAIGE version wzhou88/saige:1.1.9 (ref. ^61^). In the sex-combined analyses, we account for age, sex, age^2^, age × sex, age^2^ × sex and the first 10 genetic principal components as fixed effects; and age, age^2^ and the first 10 principal components in sex-specific analyses. All continuous traits were inverse rank normalized before association testing.

For SAIGE step 0, we constructed a genetic relatedness matrix (GRM) using the UKBB genotyping array data. We LD pruned the genotyped data using PLINK (–indep-pairwise 50 5 0.05)^86^ and created a sparse GRM using 5,000 randomly selected markers, with relatedness cutoff of 0.05, using the createSparseGRM.R function within SAIGE. To generate a variance ratio file for subsequent steps in SAIGE, we extracted 1,000 variants each with minor allele count (MAC) <20 and MAC >20, and combined these markers to define a PLINK file for the variance ratio determination.

In SAIGE step 1 for each trait, the curated phenotype data and sparse GRM were used to fit a null model with no genetic contribution. All parameters were set at the defaults in SAIGE, except --relatednessCutoff 0.05, --useSparseGRMtoFitNULL TRUE and --isCateVarianceRatio TRUE. Tolerance for fitting the null generalized linear mixed model was set to 0.00001.

##### Rare-variant and gene-based testing

Following null model fitting, we carried out variant and gene-based testing in SAIGE step 2 using the variant categories described above, with the --is_single_in_groupTest TRUE flag. All other parameters were set to default, except --maxMAF_in_groupTest=0.0001,0.001,0.01, --is_Firth_beta TRUE, --pCutoffforFirth=0.1 and --is_fastTest TRUE. We included the following collection of group tests, using the annotations defined above (see ‘Variant annotation’ section).

- High confidence pLoF
- Damaging missense/protein altering
- Other missense/protein altering
- Synonymous
- High confidence pLoF or damaging missense/protein altering
- High confidence pLoF or damaging missense/protein altering or other missense/protein altering or synonymous

We then carried out Cauchy combination tests^87^ across these annotations for each gene.

#### Conditioning on nearby common variants

We conditioned rare variants that reached significance in exome-wide analyses (*P* < 1 × 10^–7^, Bonferroni adjustment for ~500,000 independent variants) on nearby common lead variants (±500 kb with MAF >1%), identified from the GWAS meta-analyses reported in this manuscript. The identified lead SNPs were included as covariates in SAIGE variant-based tests step 2.

#### Replication of exome-sequencing analyses

QC, variant annotation and genetic association testing at the gene level in the G&H whole-exome sequencing (WES) data were performed identically to UKBB, with any deviations noted below. The total sample size available was 44,028 (24,444 females and 19,584 males) with phenotype-specific sample sizes provided in Supplementary Tables 2 and 9. Sample sizes for WES analyses in G&H differ from sample sizes for GWASs because (1) further samples were added during the period between analyses and (2) not all G&H samples with genotyping have been exome sequenced as yet. A total of 4,723,926 variants passed QC. Genetic association analysis covariates were age, sex, age^2^ and the first 20 genetic principal components as fixed effects; and age, age^2^ and the first 20 principal components in sex-specific analyses.

Variant annotation, gene-burden models and association analyses performed in deCODE are described in the Supplementary Note.

### Reporting summary

Further information on research design is available in the Nature Portfolio Reporting Summary linked to this article.

## Online content

Any methods, additional references, Nature Portfolio reporting summaries, source data, extended data, supplementary information, acknowledgements, peer review information; details of author contributions and competing interests; and statements of data and code availability are available at 10.1038/s41588-025-02156-8.

## Supplementary information

Supplementary Information Supplementary Figs. 1–20 and Note

Reporting Summary

Peer Review File

Supplementary Table 1 Supplementary Tables 1–25.

## Data Availability

Cohorts may be contacted individually for access to raw data. Meta-analysis summary statistics for all phenotypes are available through the GWAS Catalog study accession nos. GCST90483463 to GCST90483502.

## Code availability

All code used in this study is available via GitHub at https://github.com/lindgrengroup/infertility_hormones and via Zenodo at 10.5281/zenodo.14528258 (ref. ^88^).
