# Supplementary material for: Genome-wide analyses identify 25 infertility loci and relationships with reproductive traits across the allele frequency spectrum
Source: Nat Genet. 2025 Apr 14;57(5):1107–18. doi: 10.1038/s41588-025-02156-8 (PMC12081293; doi:10.1038/s41588-025-02156-8)
Supplement: Supplementary file 1 — Supplementary Figs. 1–20 and Note [file 41588_2025_2156_MOESM1_ESM.pdf]

nature genetics

Supplementary information

https://doi.org/10.1038/s41588-025-02156-8

Genome-wide analyses identify 25 infertility loci and relationships with reproductive traits across the allele frequency spectrum

In the format provided by the authors and unedited

Supplementary Figures

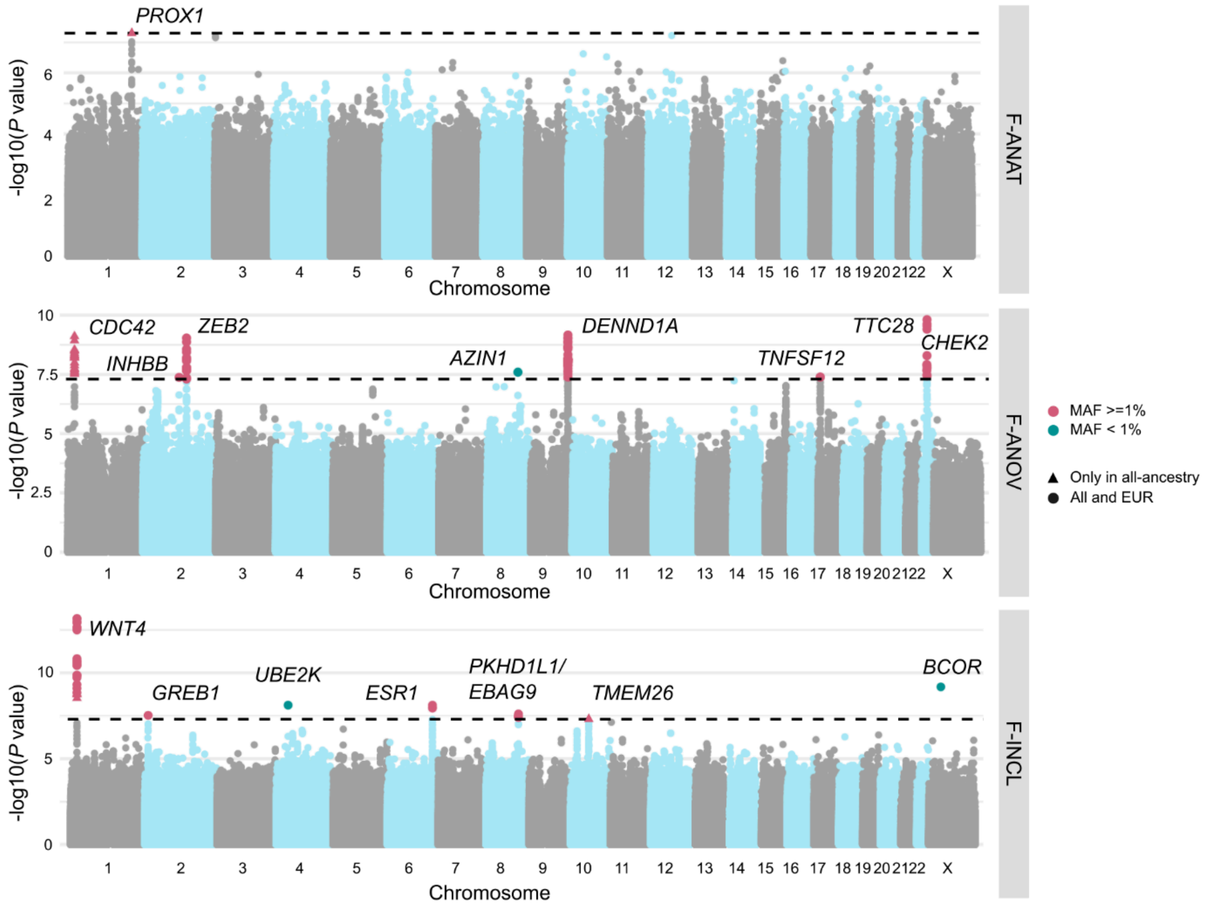

Supp. Figure 1. Manhattan plots for female infertility GWAS meta-analyses not presented in the main text. Genetic variants associated with anatomical female infertility (F-ANAT) (top), anovulatory female infertility (F-ANOV) (middle), and idiopathic infertility (unknown causes) defined by inclusion of a code for idiopathic infertility (F-INCL) (bottom). Each point depicts a single SNP, with genome-wide significant (GWS) SNPs (P < 5E-08, dashed line representing the multiple-testing corrected P-value threshold of P<5E-08, accounting for approx. 1 million independent variants in the genome) coloured in pink for common variants with minor allele frequency (MAF)>=1% and green for those with MAF<1%. Summary statistics from whole-genome regression analyses were meta-analysed using fixed-effect inverse-variance weighting in the METAL software to produce the displayed P-values. Triangles represent SNPs that only reach GWS in all-ancestry GWAS meta-analyses. SNPs are annotated with the mapped gene.

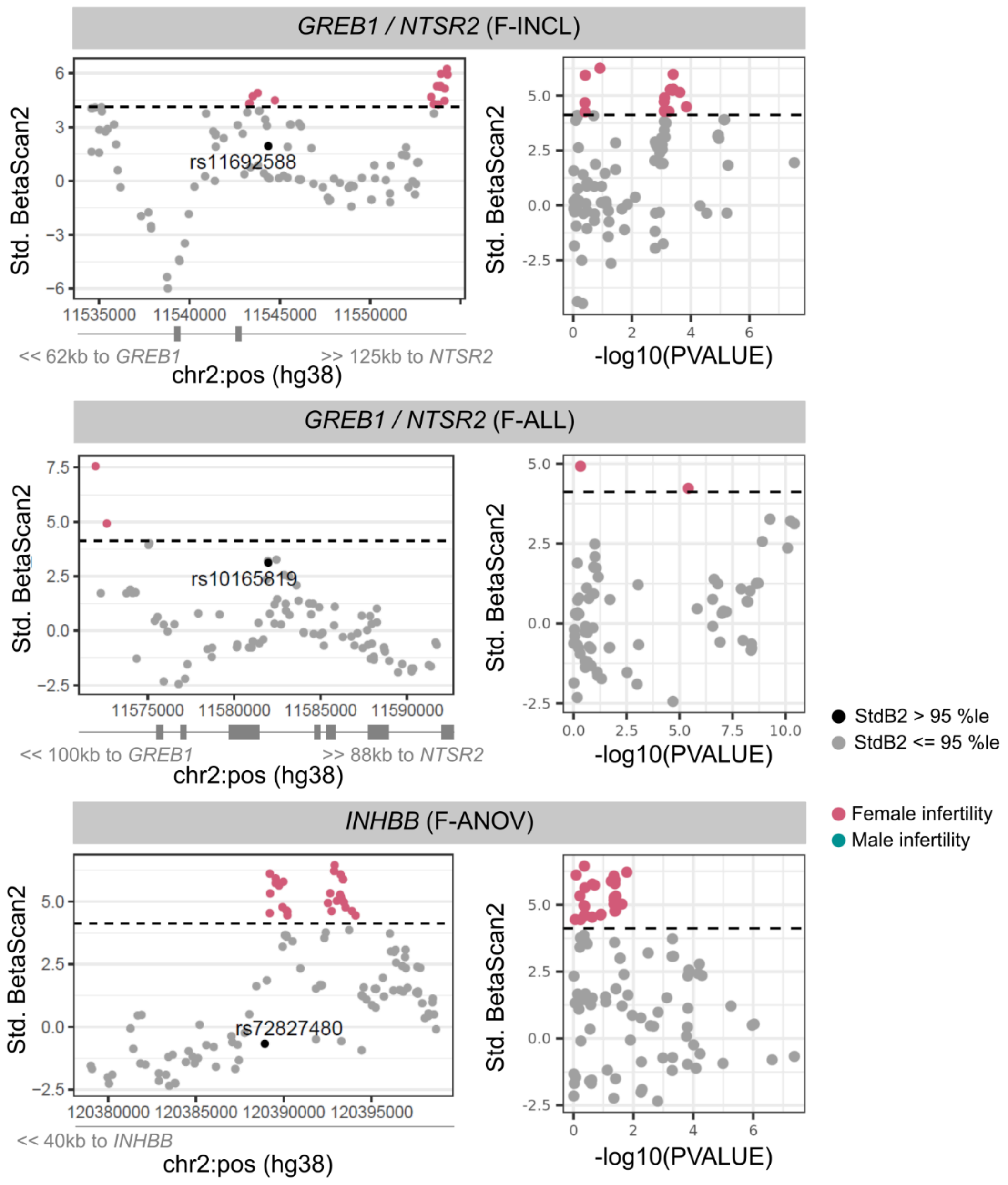

Supp. Figure 2. Balancing selection, as measured by standardised BetaScan2 (StdB2) scores, at infertility-associated loci. Each panel displays windows of +/- 10 kb around a lead infertility-associated variant, annotated with nearest gene and location: rs10165819 (F-ALL), rs72827480 (anovulatory infertility, F-ANOV), and rs11692588 (female idiopathic infertility by inclusion, F-INCL). Dashed lines indicate 95th %ile of StdB2, and variants crossing this threshold are coloured in pink (for female infertility loci) or green (for the male infertility locus). Left: Locus plots depicting genomic position on the x-axis and StdB2 on the y-axis. Right: Scatter plots depicting relationship between -log10 of the GWAS p-value for the variant association with infertility on the x-axis and StdB2 on the y-axis.

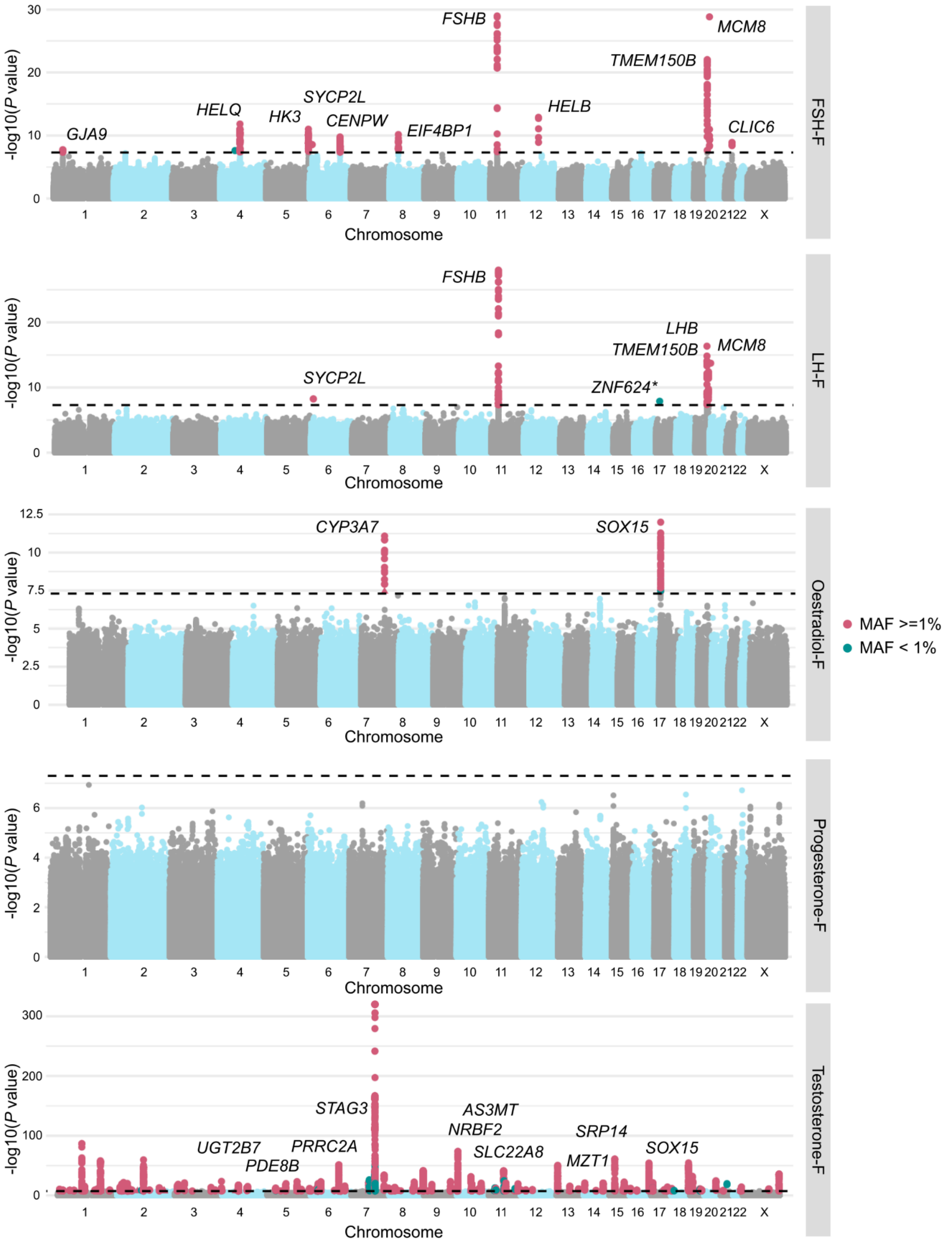

Supp. Figure 3. Manhattan plots for female reproductive hormone GWAS meta-analyses. Each panel displays genetic variants associated with a different reproductive hormone, from top to bottom: follicle stimulating hormone (FSH), luteinising hormone (LH), oestradiol, progesterone, and total testosterone. Each point depicts a single SNP, with genome-wide significant (GWS) SNPs (P < 5E-08, dashed line representing the multiple-testing corrected P-value threshold of P<5E-08, accounting for approx. 1 million independent variants in the genome) coloured in pink for common variants with minor allele frequency (MAF)>=1% and green for those with MAF<1%. Summary statistics from whole-genome regression analyses were meta-analysed using fixed-effect inverse-variance weighting in the METAL software to produce the displayed P-values. SNPs are annotated with the mapped gene; for testosterone, only novel SNPs (ten lowest P-values) are annotated. * indicates that lead variant is present in only one study. For all lead variants, refer to Supp. Table 10.

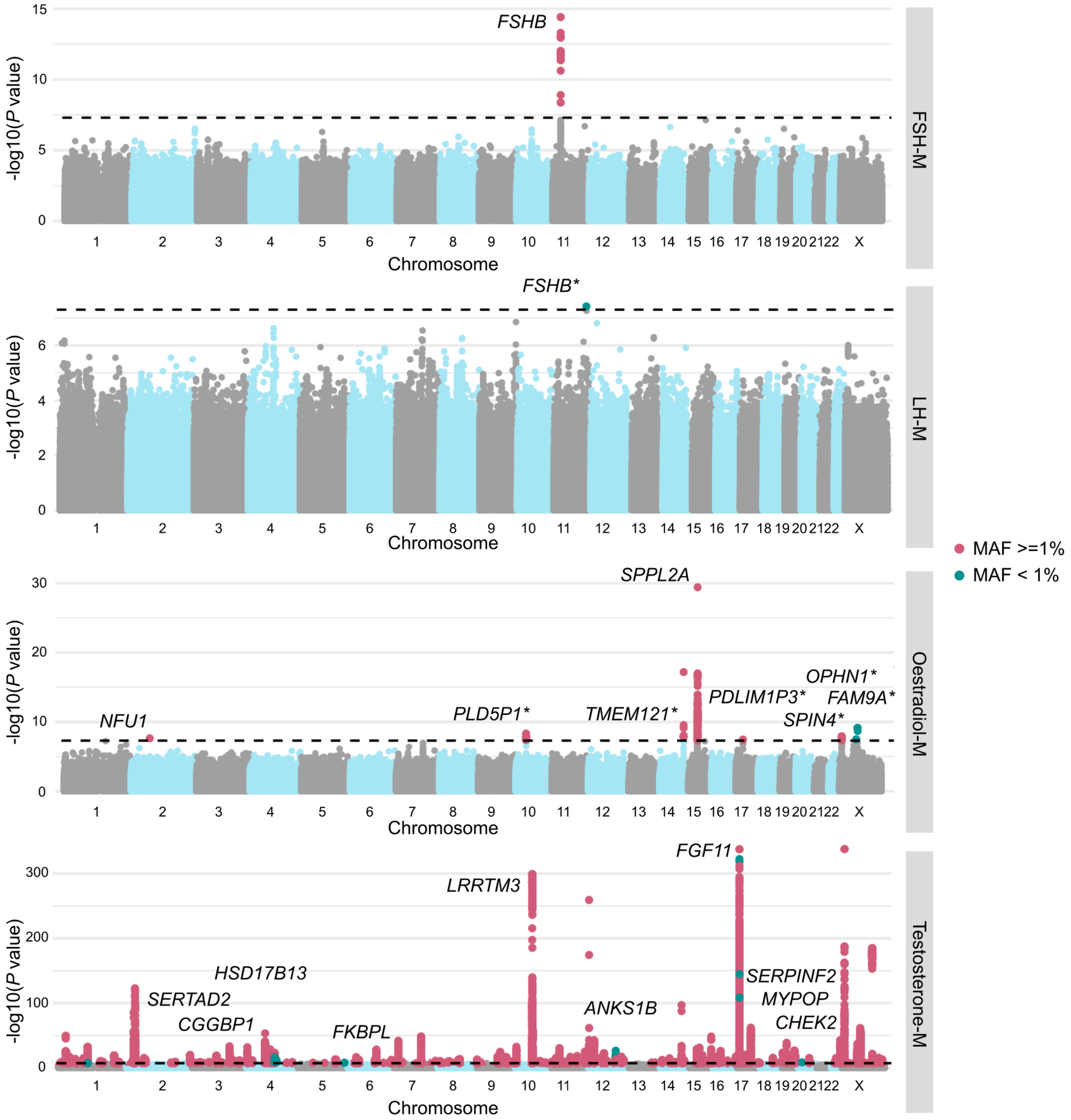

Supp. Figure 4. Manhattan plots for male reproductive hormone GWAS meta-analyses. Each panel displays genetic variants associated with a different reproductive hormone, from top to bottom: follicle stimulating hormone (FSH), luteinising hormone (LH), oestradiol, and total testosterone. Each point depicts a single SNP, with genome-wide significant (GWS) SNPs (P < 5E-08, dashed line representing the multiple-testing corrected P-value threshold of P<5E-08, accounting for approx. 1 million independent variants in the genome) coloured in pink for common variants with minor allele frequency (MAF)>=1% and green for those with MAF<1%. Summary statistics from whole-genome regression analyses were meta-analysed using fixed-effect inverse-variance weighting in the METAL software to produce the displayed P-values. SNPs are annotated with the mapped gene; for testosterone, only novel SNPs (ten lowest P-values) are annotated. * indicates that lead variant is present in only one study. For all lead variants, refer to Supp. Table 10.

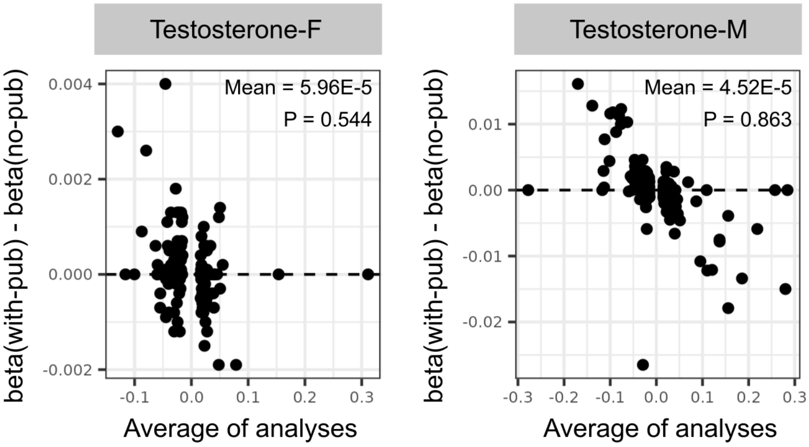

Supp. Figure 5. Comparison of effect sizes of SNPs associated with testosterone in main meta-analyses and sensitivity analyses without publicly available summary statistics. Variants that are genome-wide significant (GWS *P* < 5E-08) in the main GWAS with public data are plotted. The Bland-Altman plot displays the difference between effect sizes estimated in the with-public data (with-pub) and no-public data (no-pub) GWASs for each variant, plotted against the mean estimate from the two sets of analyses. The mean difference and one-sample, two-sided t-test P-value (with null hypothesis of mean difference = 0) are displayed for each of the strata. Left: female-specific analyses, Right: male-specific analyses, from all-ancestry samples.

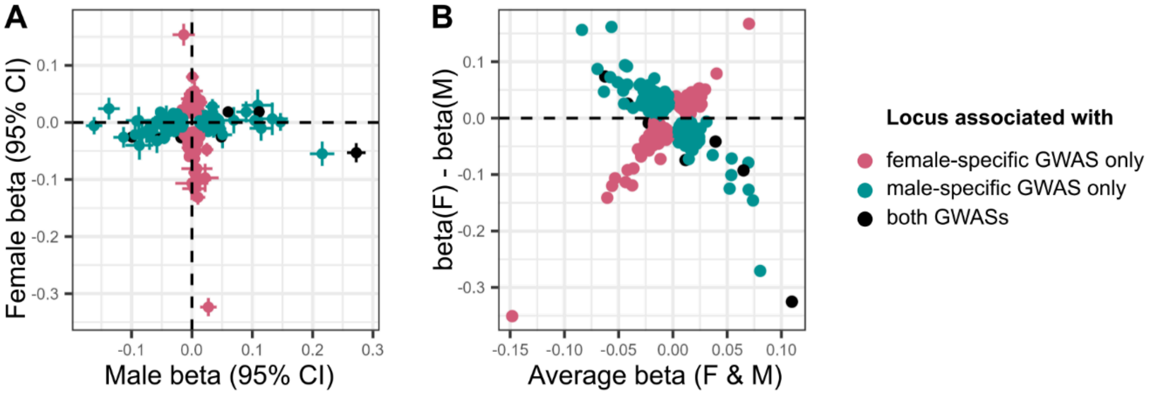

Supp. Figure 6. Sex heterogeneity in the effects of lead variants associated with testosterone. Lead variants in either female-specific testosterone GWAS (coloured in pink), male-specific testosterone GWAS (coloured in green), or both (coloured in black) are displayed. Results are from all-ancestry meta-analyses. (A) Scatter plot comparing effect sizes and 95% confidence intervals (CIs), plotted as error bars, of GWS variants. (B) Bland-Altman plot displaying the difference between effect sizes estimated in the female-specific and male-specific GWASs for each variant, plotted against the mean estimate from both GWASs.

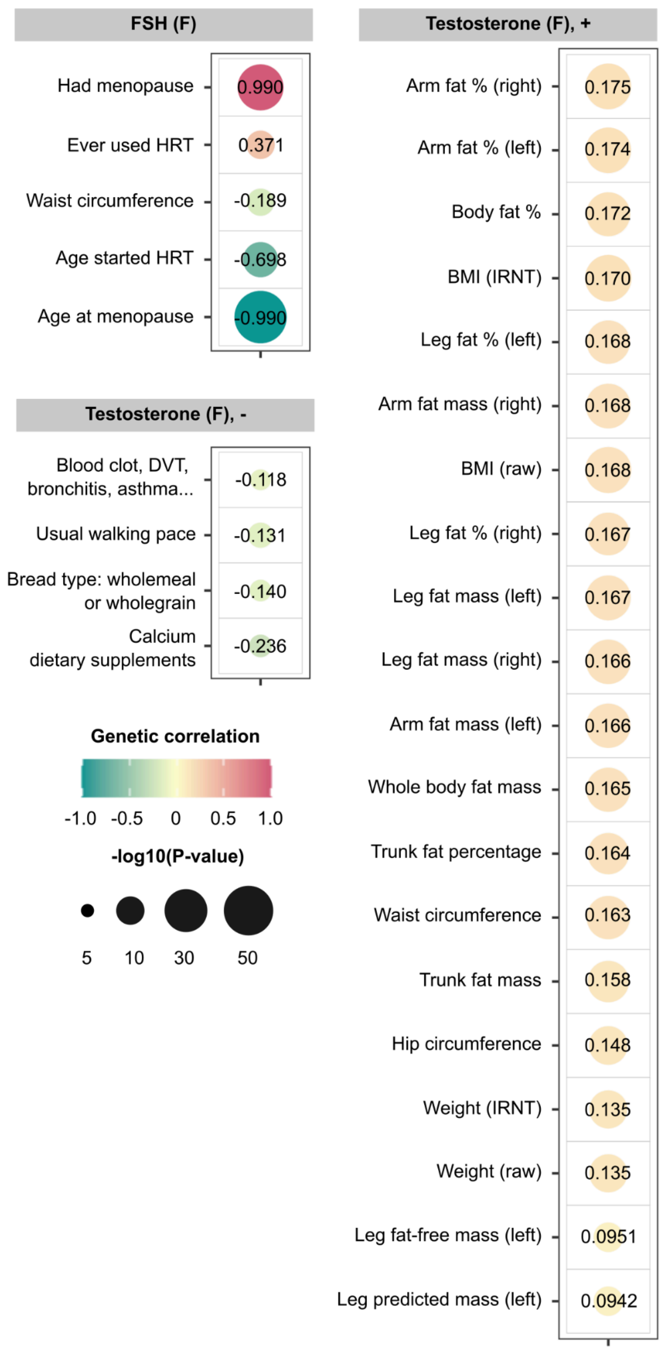

Supp. Figure 7. Genetic correlations (r_g) between follicle stimulating hormone in females (FSH-F) or testosterone-F and traits across the phenome. Trait summary statistics were generated by the Neale lab[1] and SNP-based genetic correlation calculations were performed using the LDSC software on a subset of 1 million HapMap3 SNPs[2]. Points are coloured by r_g estimate and sized by -log10(P). Only phenotypes associated at P < 2.45E-05 (FWER controlled at 5% across 2,040 tests using the Bonferroni method, accounting for 340 effectively independent UKBB phenotypes and 6 hormone or infertility strata are displayed, up to a maximum of 20 phenotypes. Phenotypes in the panel labelled “Testosterone (F), -” are negatively correlated with testosterone-F, and those in the panel labelled “Testosterone (F), +” are positively correlated with testosterone-F. HRT = hormone replacement therapy, BMI = body mass index, IRNT = inverse-rank normally transformed, “Blood clot, DVT, bronchitis, asthma…” = Blood clot, deep vein thrombosis (DVT), bronchitis, emphysema, asthma, rhinitis, eczema, Hayfever, allergic rhinitis or eczema allergy diagnosed by doctor.

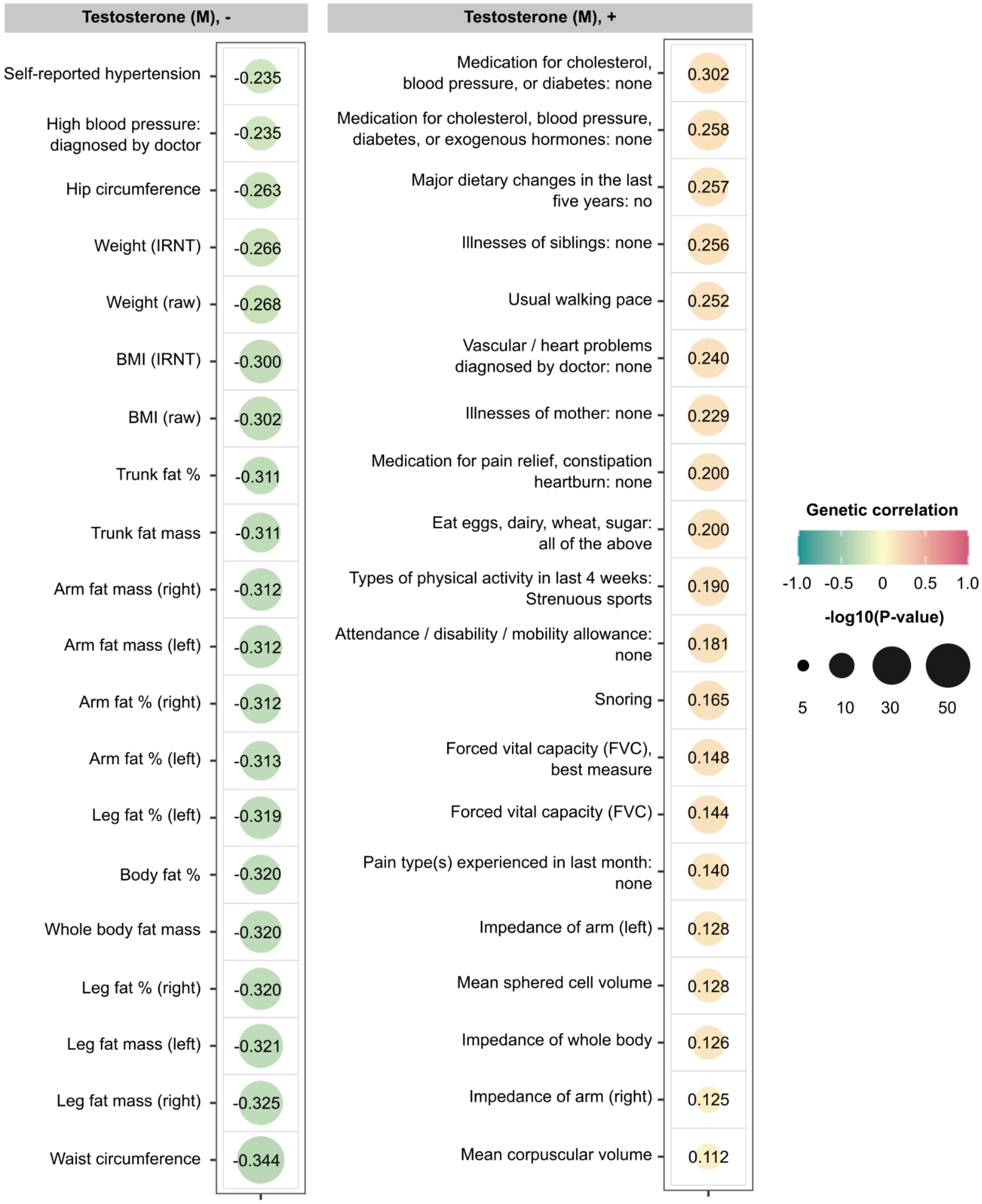

Supp. Figure 8. Genetic correlations (r_g) between testosterone in males and traits across the phenome. Trait summary statistics were generated by the Neale lab[1] and SNP-based genetic correlation calculations were performed using the LDSC software[3] on a subset of 1 million HapMap3 SNPs[2]. Points are coloured by r_g estimate and sized by -log10(P). Only phenotypes associated at $P <$ 2.45E-05 (FWER controlled at 5% across 2,040 tests using the Bonferroni method, accounting for 340 effectively independent UKBB phenotypes and 6 hormone or infertility strata are displayed, up to a maximum of 20 phenotypes. Phenotypes in the panel labelled “Testosterone (M), -” are negatively correlated with testosterone-M, and those in the panel labelled “Testosterone (M), +” are positively correlated with testosterone-M. BMI = body mass index, IRNT = inverse-rank normally transformed.

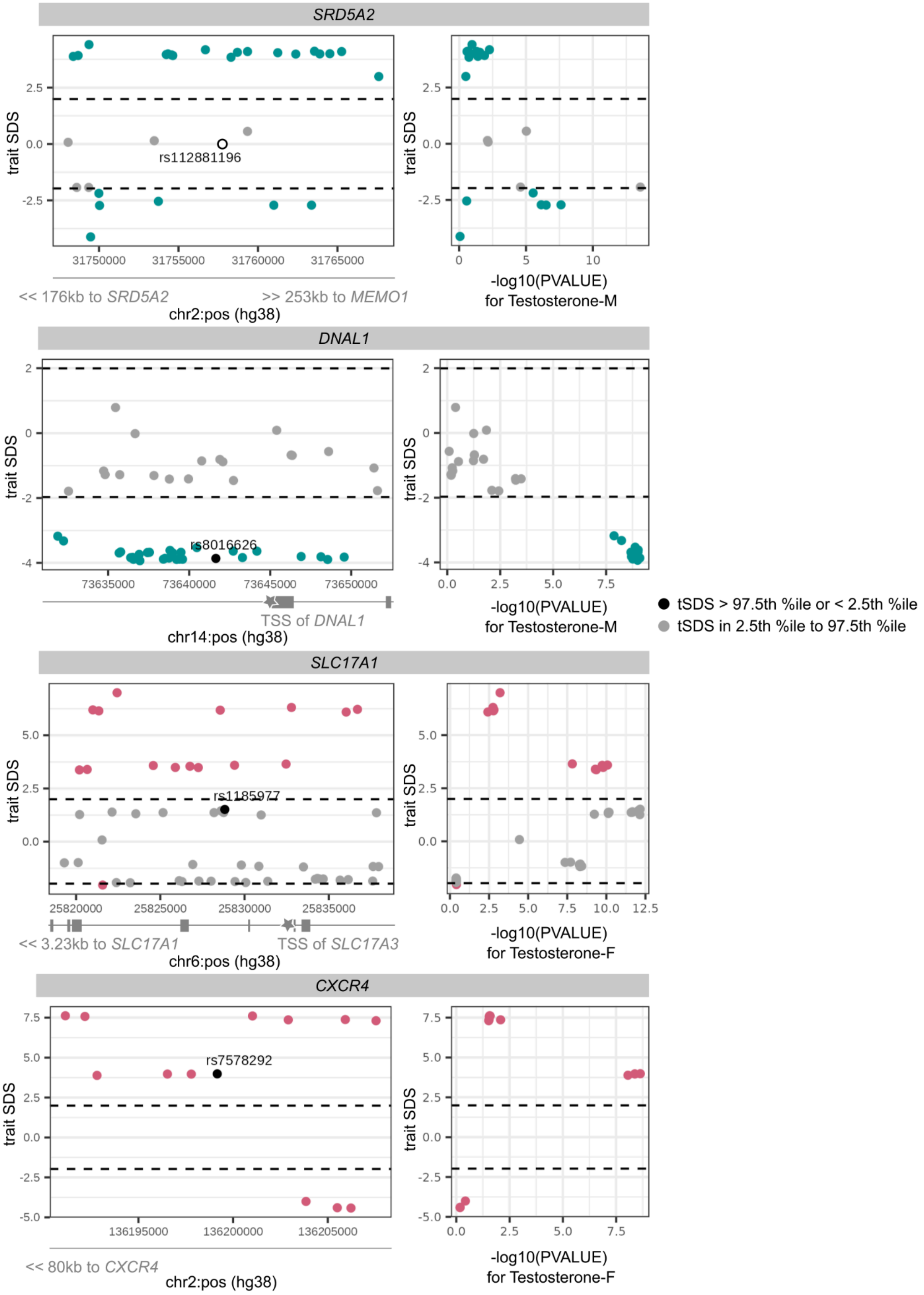

Supp. Figure 9. Trait-aligned Singleton Density Scores (SDSs), measuring recent directional selection, at testosterone-associated loci. Each panel displays windows of +/- 10 kb around a lead testosterone-associated variant, annotated with the location of nearest gene transcription start sites (TSSs) for all variants with extreme tSDSs: rs112881196 and rs8016626 (male-specific), and rs1185977 and rs7578292 (female-specific). The tSDSs are aligned to the testosterone-increasing allele, wherein a positive tSDS indicates positive selection for testosterone-increasing allele at the locus. Dashed lines indicate 2.5th percentile (%ile) and 97.5th %ile of SDSs, and variants below or above this threshold respectively are coloured in green (for male-specific loci) and pink (for female-specific loci). Left: Locus plots depicting genomic position on the x-axis and trait-SDS on the y-axis. The lead variant rs112881196 (open circle) is not present in the tSDS dataset and thus assigned a score of 0. Right: Scatter plots depicting relationship between -log10 of the GWAS p-value for the variant association with testosterone on the x-axis and tSDS on the y-axis.

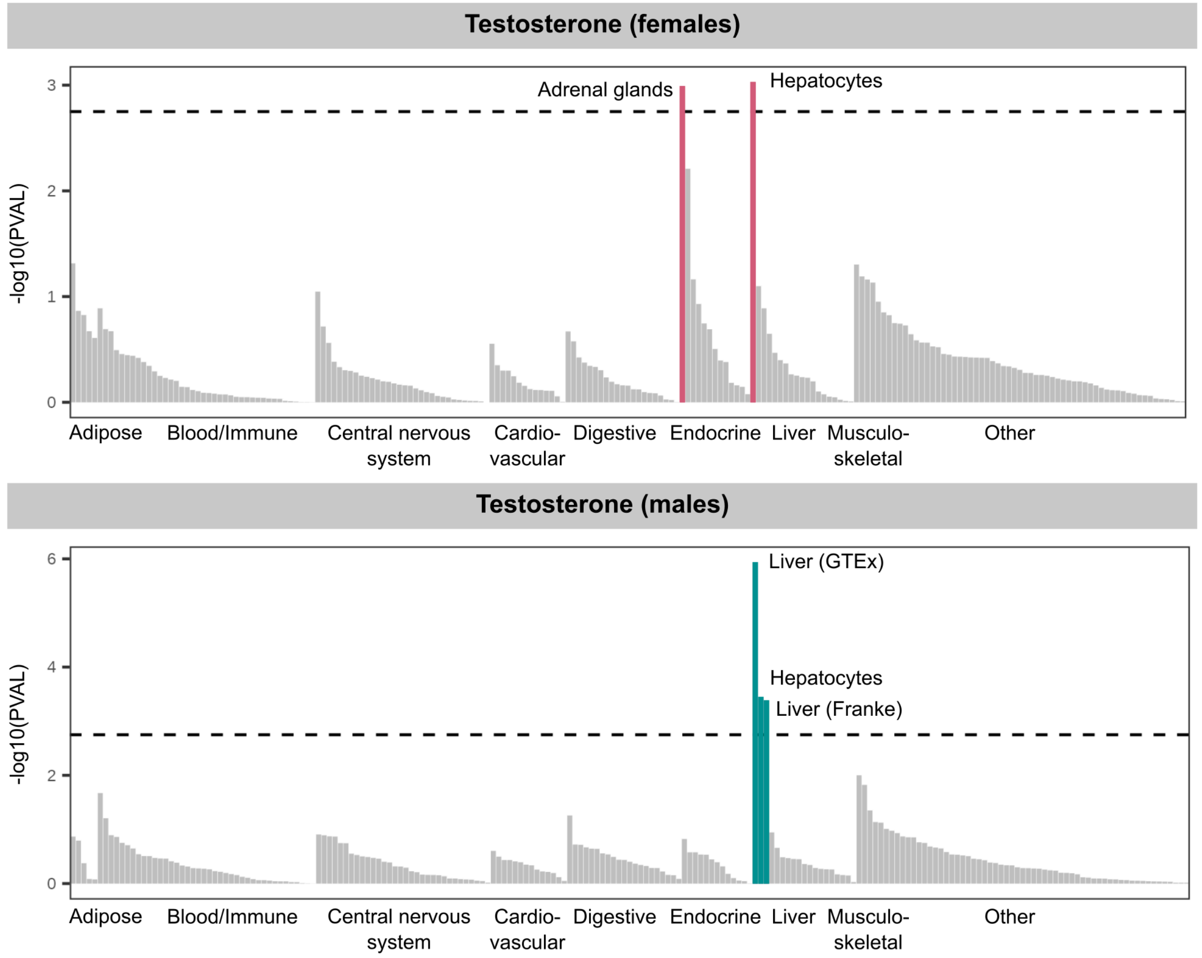

Supp. Figure 10. Enrichment of testosterone heritability across tissues and cell types. Partitioned heritability across 205 tissues and cell-types from the Genotype Tissue Expression (GTEx) Project database[4] and the Franke lab single-cell database[5] was assessed using partitioned LD-score regression[3]. Tissues and cell types are broadly grouped by organ system, and those that reach significance (FDR < 5%, dashed line), are annotated and coloured in: pink for testosterone in females (top) and green for testosterone in males (bottom).

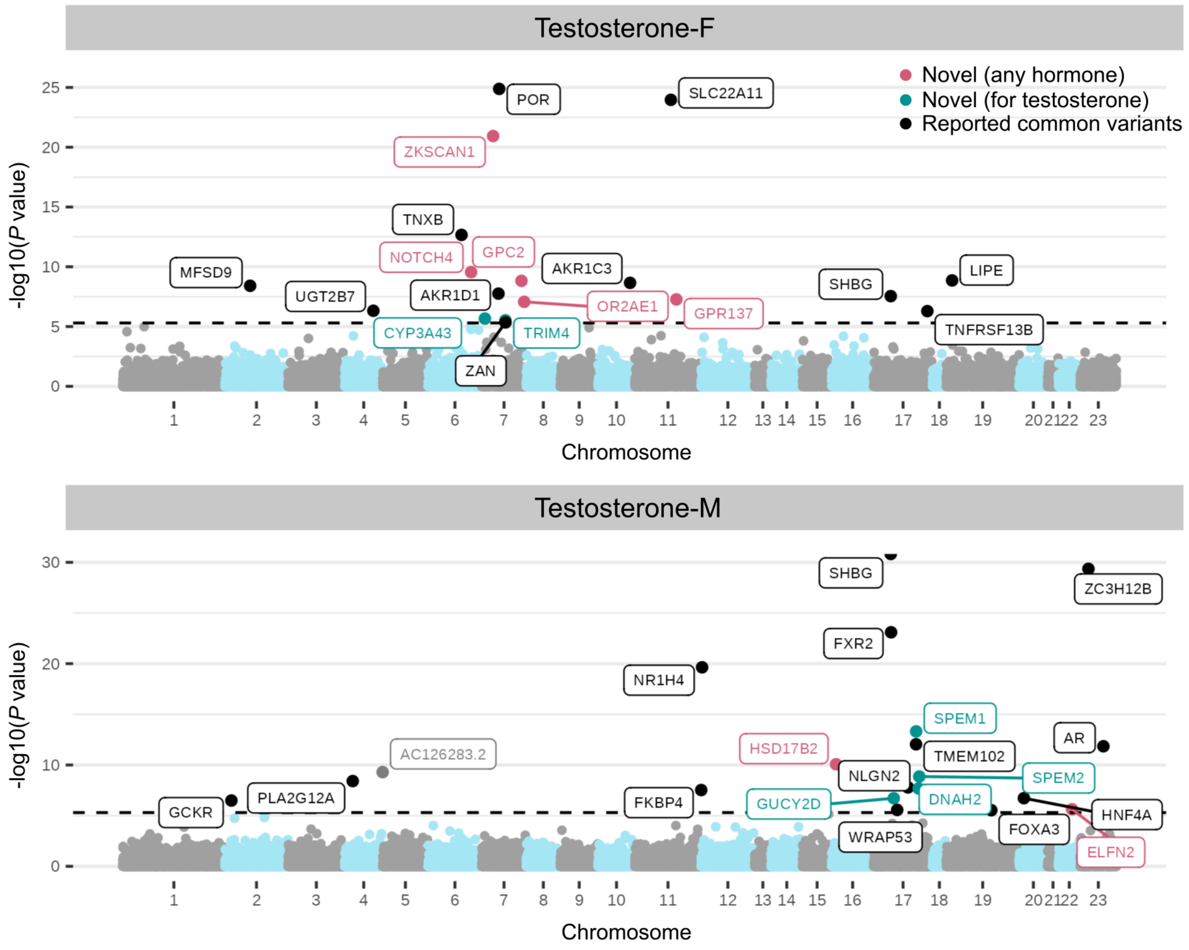

Supp. Figure 11. Gene-based Manhattan plots for burden of rare variants associated with testosterone in females and males in UK Biobank. Significance levels estimated using the Cauchy combined P-value are displayed, with significant genes (exome-wide significant at *P* < 5E-06 (FWER controlled at 5% using the Bonferroni method, across 10,000 effectively independent genes) coloured in: pink if no previous common variant associations with the gene have been reported for any of 28 reproductive hormones in the GWAS Catalog[6], green if no previous common variant associations with the gene have been reported for testosterone, and black otherwise.

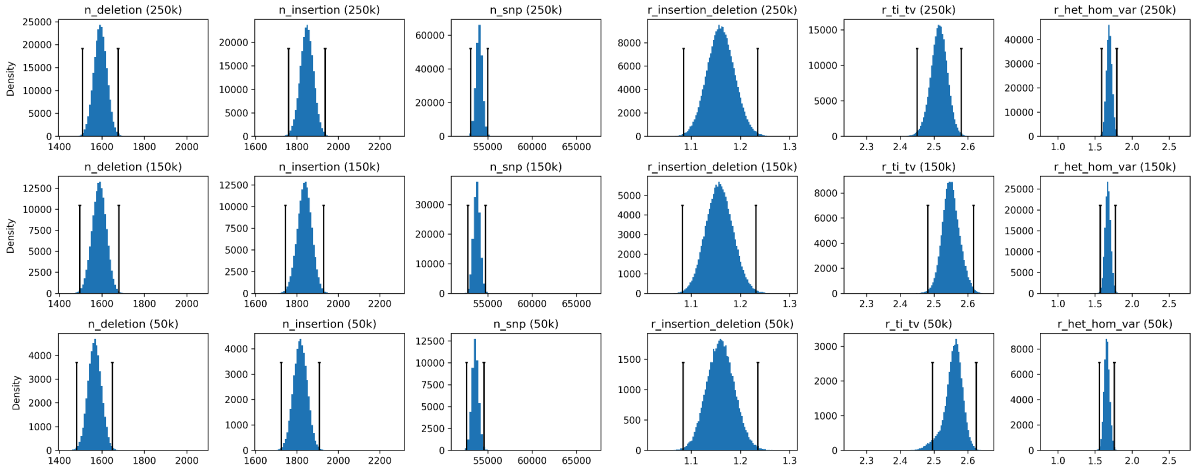

Supp. Figure 12. MAD thresholds for QC of exome sequencing samples. Samples with any of n_deletion, n_insertion, n_snp, r_insertion_deletion, r_ti_tv, and r_het_hom_var exceeding four MADs from the median are removed. MAD thresholds are displayed as vertical lines, conditional on tranche size (50k, 200k, 450k), from shortest to tallest.

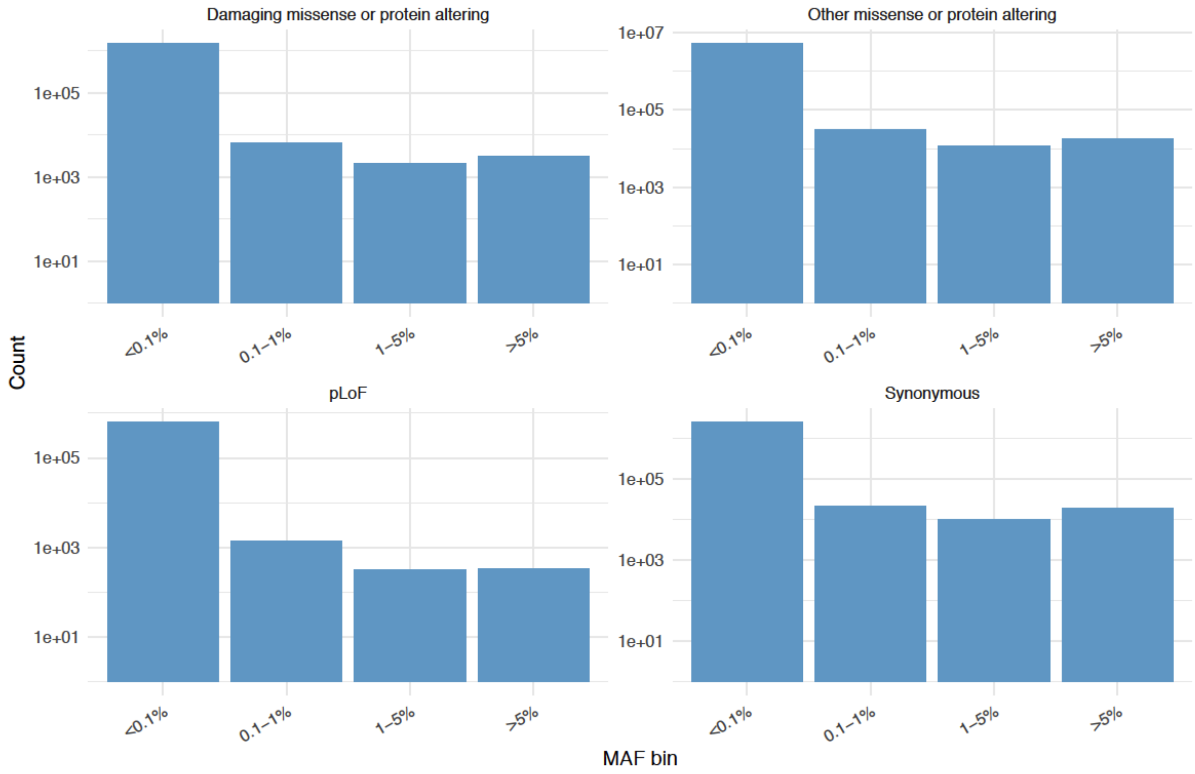

Supp. Figure 13. Average number of variants per individual, binned by MAF, according to the x-axes. Counts (y-axes) are on a log scale.

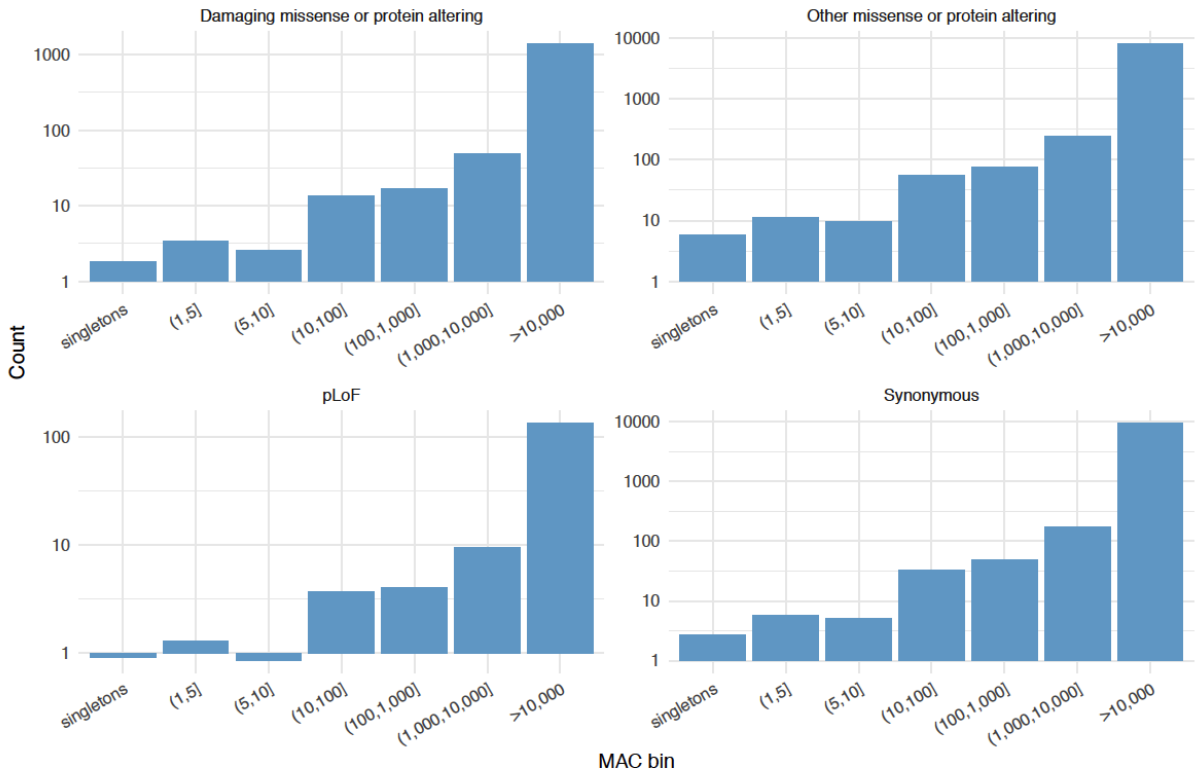

Supp. Figure 14. Average number of variants binned by minor allele count (MAC), per individual according to the *x*-axes. Count (y-axes) are on a log scale.

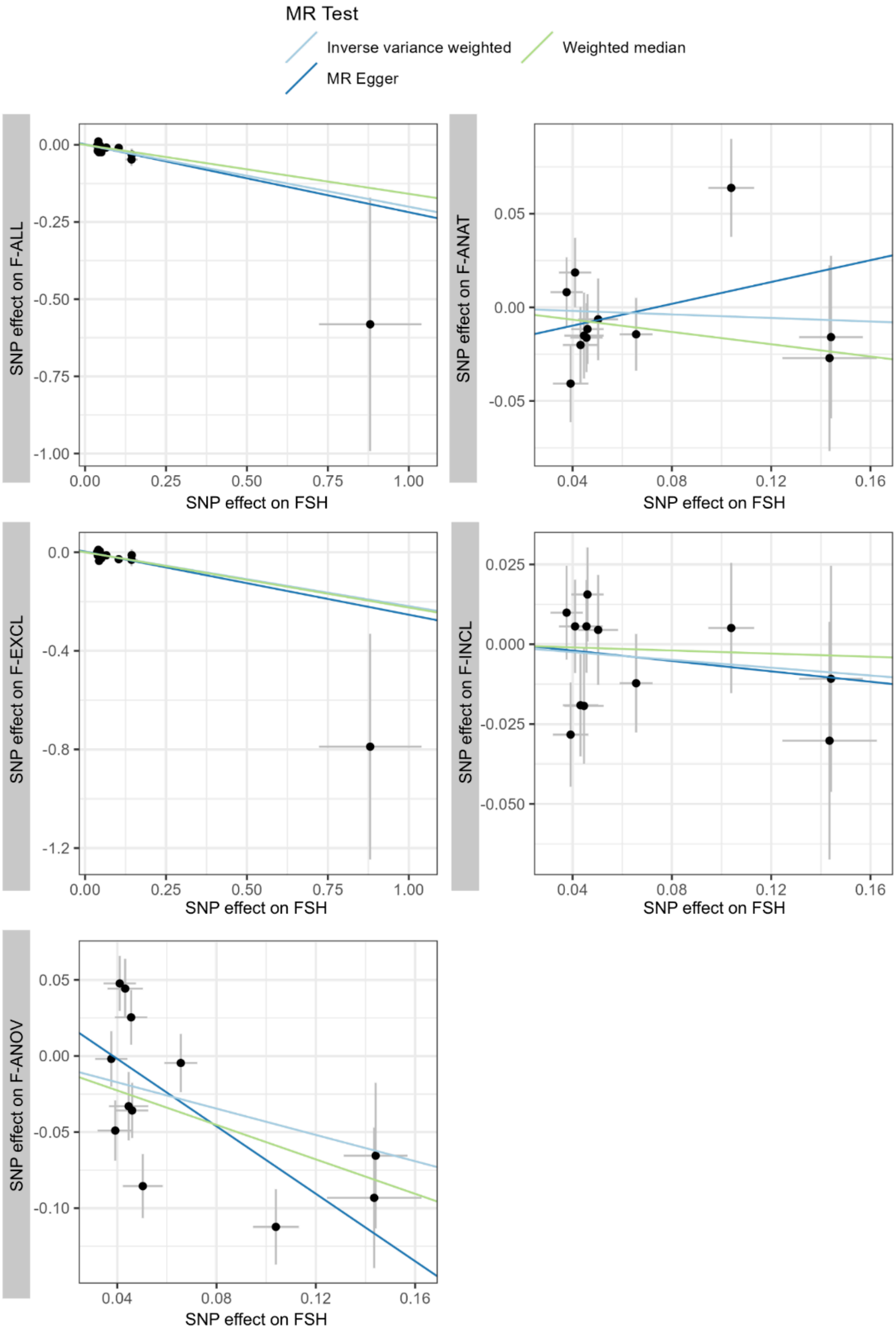

Supp. Figure 15. Scatter plots displaying genetic variants included in the female follicle stimulating hormone (FSH) instrument for Mendelian Randomisation (MR) analyses. Each panel represents a different outcome: all-cause female infertility (F-ALL), anatomical infertility (F-ANAT), anovulatory infertility (F-ANOV), idiopathic infertility defined by exclusion of known causes (F-EXCL), and idiopathic female infertility defined by inclusion of a diagnostic code for idiopathic infertility (F-INCL). Each point represents a single genetic variant in the FSH instrument, with effect sizes represented as black points and 95% confidence intervals for the exposure (x-axis) and outcome (y-axis) displayed with grey lines. MR estimates from three methods: inverse-variance weighted (light blue), MR-Egger (dark blue), and weighted median (green) are plotted.

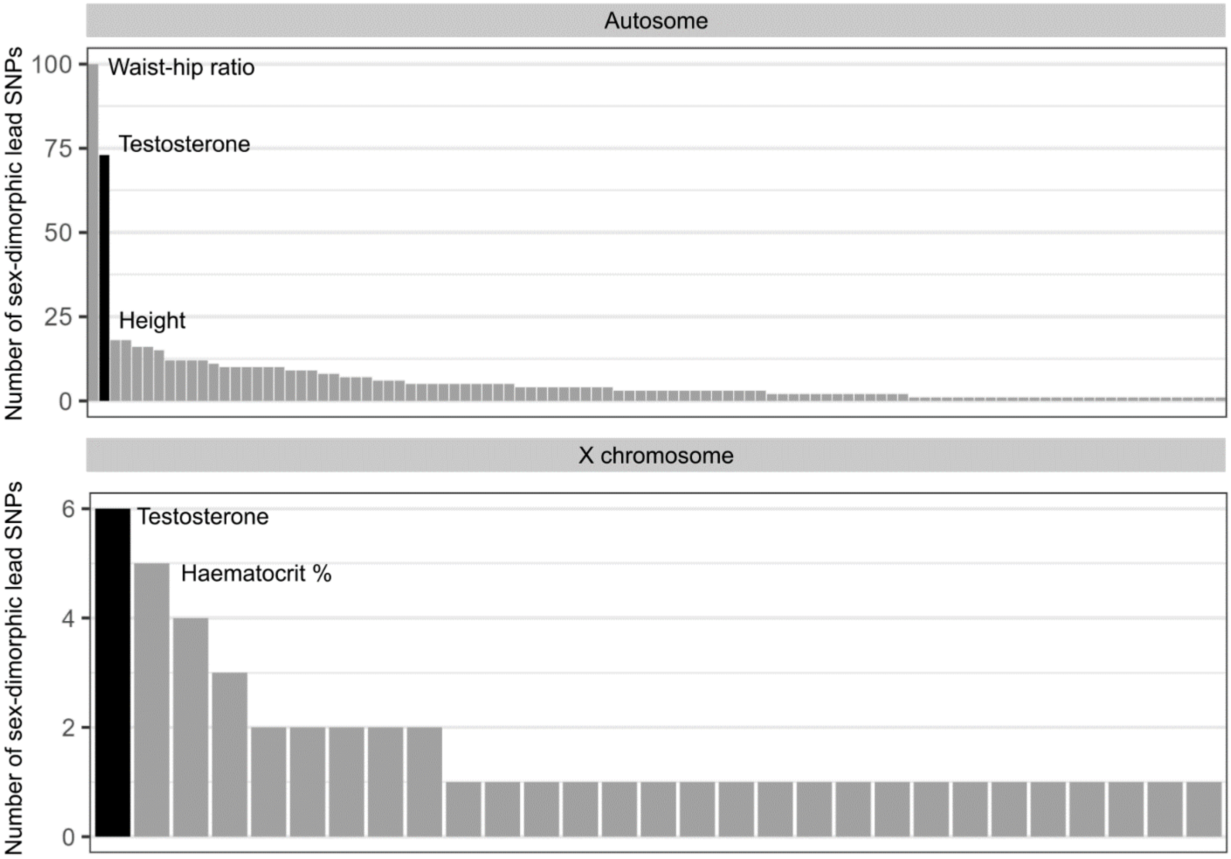

Supp. Figure 16. Number of sex-dimorphic lead SNPs (sdSNPs) for testosterone as compared to other traits in UK Biobank. Top: autosomal variants, bottom: X-chromosome variants. A lead SNP (that reached genome-wide significance *P*<5E-08 in either male-specific or female-specific analysis) was classified as sex-dimorphic if its effect size was significantly different (*P*-heterogeneity<1E-08 as described by Bernabeu *et al.* (2021)) between the sexes. The number of sdSNPs for 530 traits in UK Biobank was reported by Bernabeu *et al.* (2021), coloured in grey; number of sdSNPs for testosterone (black) was calculated in this study. Only traits with at least one sdSNP are plotted.

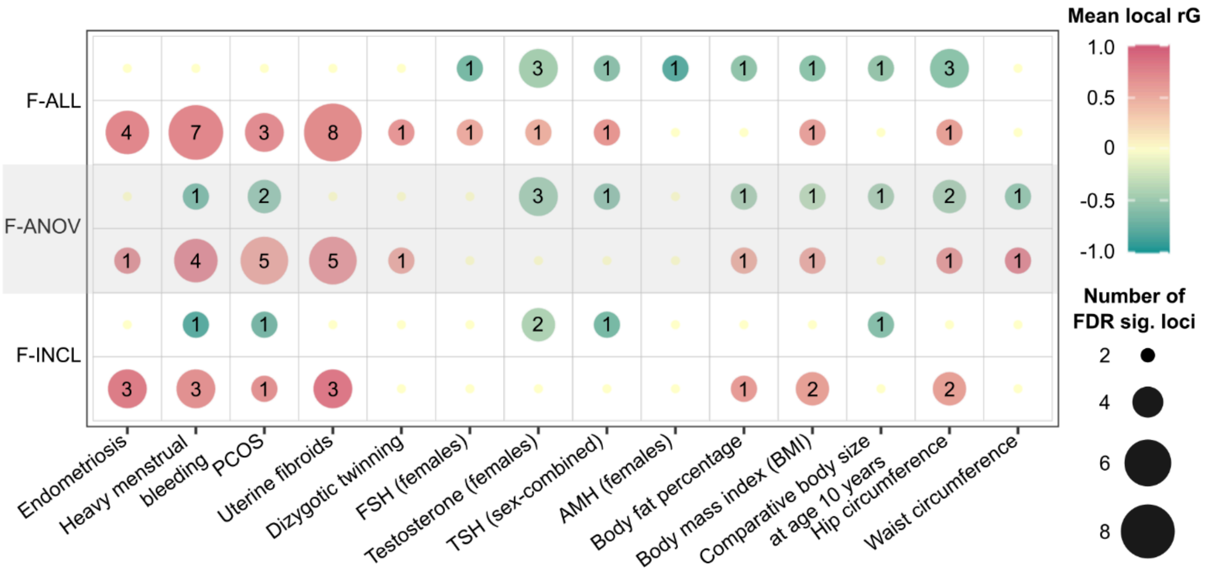

Supp. Figure 17. Number of blocks with FDR-significant bivariate local genetic correlations between female infertility (y-axis) and reproductive diseases, reproductive hormones, or obesity (x-axis). Local genetic correlations, estimated using LAVA, at 2,495 blocks across the genome with significant local heritability (P<0.05/2495) of both pairs of traits tested. The genome was partitioned into blocks of approximately 1Mb each while minimising LD between blocks. Bivariate local genetic correlations were calculated between: female infertility (all cause=F-ALL, anovulatory=F-ANOV, and idiopathic infertility defined by inclusion=F-INCL) and one of 5 reproductive conditions, 4 reproductive hormones, or 5 obesity-related traits. Associations that reach multiple-testing adjusted FDR *P*<0.05 (sig. = significant) are coloured by mean local r_g estimate (red = positive, green = negative) and points are sized and labelled by the number of loci that are FDR significant. Local genetic correlations can be found in Supp. Table 22. PCOS = polycystic ovary syndrome, FSH = follicle stimulating hormone, TSH = thyroid stimulating hormone, AMH = anti-Mullerian hormone.

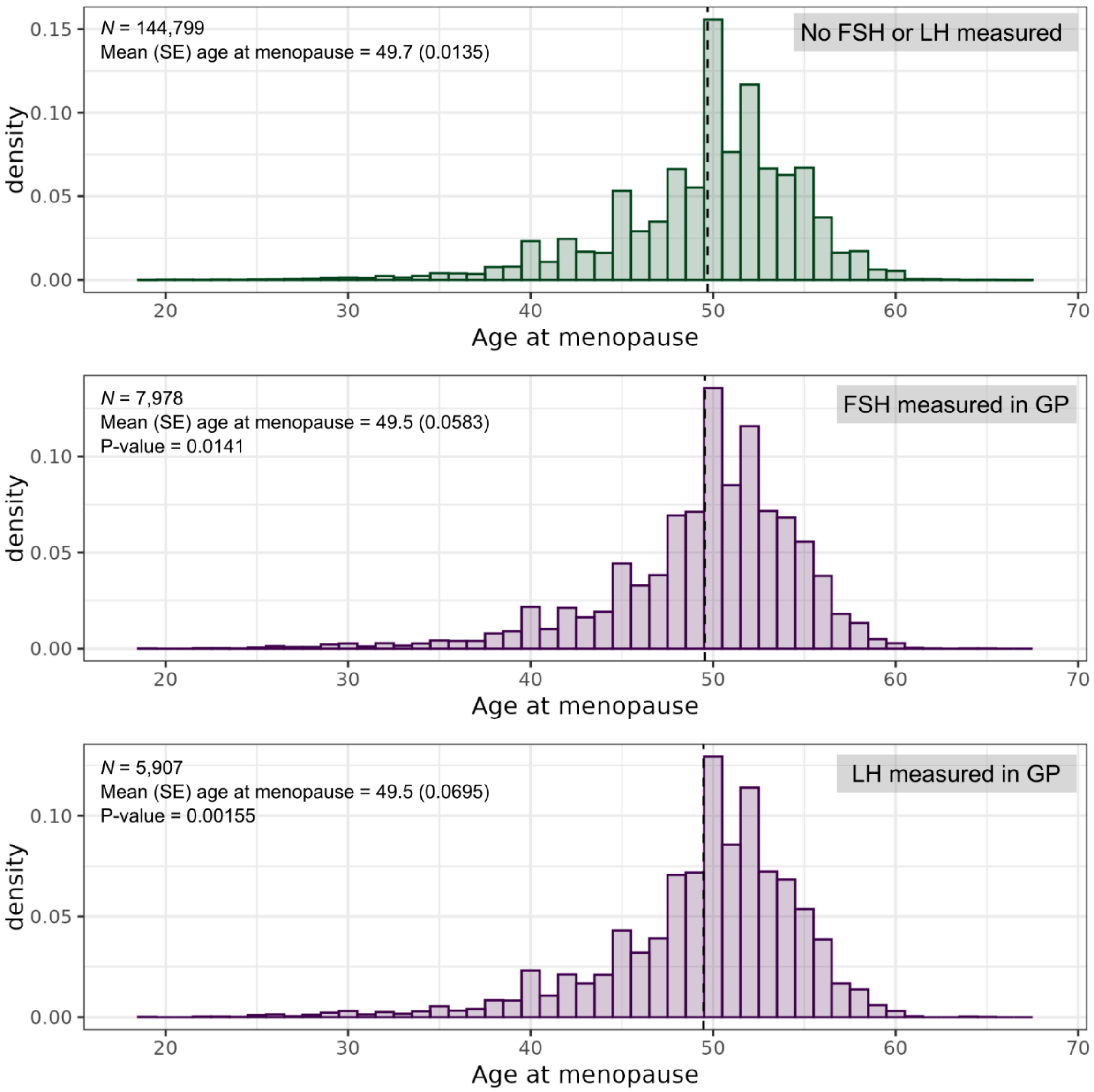

Supp. Figure 18. Distribution of self-reported age at menopause in female participants in UK Biobank with or without measurements of follicle stimulating hormone (FSH) and luteinising hormone (LH) in the linked general practice (GP) data. Histogram bins capture 1-year intervals of self-reported age at menopause. Vertical dashed lines mark the mean age at menopause in the sample. P-values represent the significance of the difference in mean age at menopause between individuals with no measurement of FSH or LH (top panel) and those with FSH (middle panel) or LH (bottom panel) measurements, estimated from two-tailed t-tests.

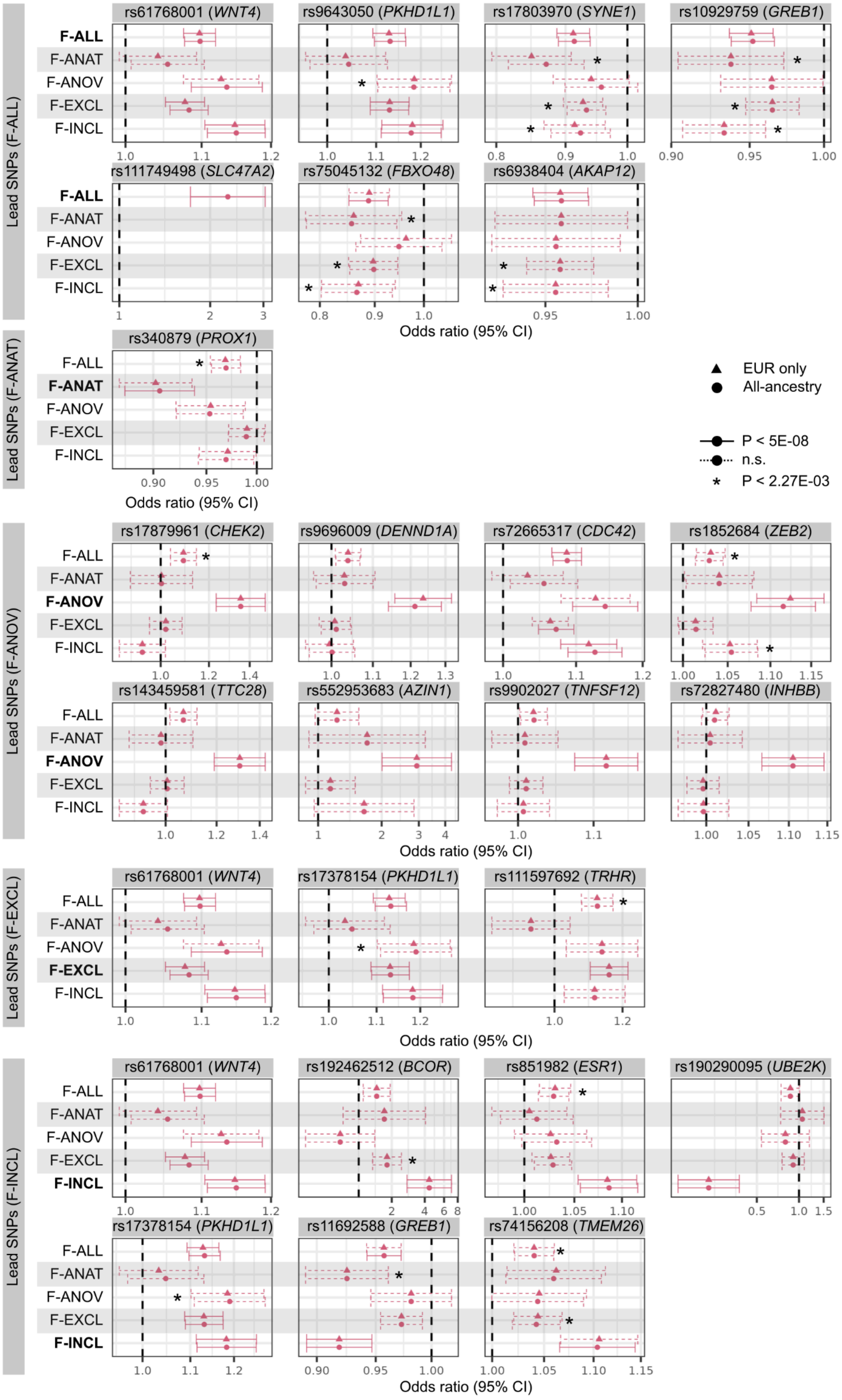

Lead SNPs (F-ALL)
rs61768001 (WNT4)
rs9643050 (PKHD1L1)
rs17803970 (SYNE1)
rs10929759 (GREB1)
rs111749498 (SLC47A2)
rs75045132 (FBXO48)
rs6938404 (AKAP12)
Lead SNPs (F-ANAT)
rs340879 (PROX1)
Lead SNPs (F-ANOV)
rs17879961 (CHEK2)
rs9696009 (DENND1A)
rs72665317 (CDC42)
rs1852684 (ZEB2)
rs143459581 (TTC28)
rs552953683 (AZIN1)
rs9902027 (TNFSF12)
rs72827480 (INHBB)
Lead SNPs (F-EXCL)
rs61768001 (WNT4)
rs17378154 (PKHD1L1)
rs111597692 (TRHR)
Lead SNPs (F-INCL)
rs61768001 (WNT4)
rs192462512 (BCOR)
rs851982 (ESR1)
rs190290095 (UBE2K)
rs17378154 (PKHD1L1)
rs11692588 (GREB1)
rs74156208 (TMEM26)
F-ALL
F-ANAT
F-ANOV
F-EXCL
F-INCL
Odds ratio (95% CI)
EUR only
All-ancestry
P < 5E-08
n.s.
* P < 2.27E-03

Supp. Figure 19. Forest plots displaying the effect of lead SNPs associated with female infertility phenotypes across all categories of female infertility. Each panel represents a lead SNP, with odds ratio (OR) and 95% confidence interval (CI) for the effect size of that SNP on the x-axis and female infertility category on the y-axis. Summary statistics from whole-genome regression analyses were meta-analysed using fixed-effect inverse-variance weighting in the METAL software to produce the displayed statistics.Triangles represent effect sizes in the EUR-ancestry meta-analysis, and circles for the all-ancestry meta-analysis. Solid lines represent genome-wide significant associations (P<5E-08, accounting for approx. 1 million independent variants in the genome), while dashed lines represent effects that do not reach this threshold. Marked with an asterisk (*) are sets of associations that are not genome-wide significant but reach P<2.27E-03 (Bonferroni correction for 22 independent loci tested) in either the EUR or all-ancestry meta-analyses. The grey bar on the left names the infertility category for which the lead SNP was identified.

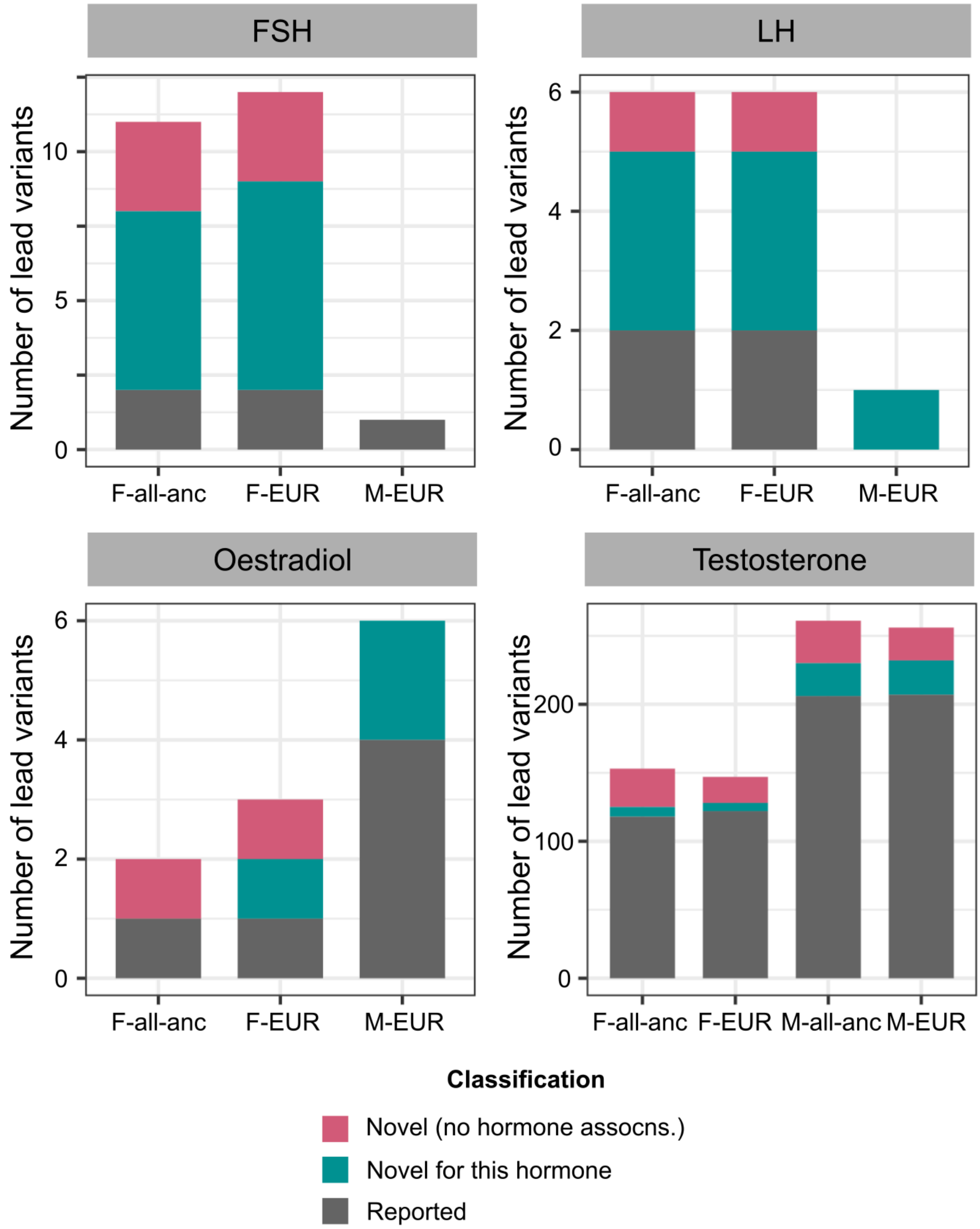

Supp. Figure 20. Number of novel and reported reproductive hormone associations. Each panel displays a different hormone (FSH=follicle-stimulating hormone, LH=luteinising hormone). Lead variants in each analysis stratum (F=female-specific, M=male-specific, all-anc=all ancestry meta-analysis, EUR=European-only meta-analysis) are classified as: (1) novel (no hormone associations) if they are not in LD (r^2<0.1) with, and conditionally independent of (conditional *P*-value P_{cond}<0.05), any variants within a 1Mb window of the lead variant that are associated with 28 reproductive hormones in the GWAS Catalog[6], plotted in pink, (2) novel for this hormone if they are not in LD (r^2<0.1) with, and conditionally independent of (P_{cond}<0.05), the respective hormone-associated variants within a 1Mb window of the lead variant, plotted in green, and (3) reported otherwise, plotted in grey. Note the different Y-axis scales in each subplot. assocns.=associations.

Supplementary Note

Supplementary Results

No evidence for heterogeneity in variant effects on infertility or reproductive hormones across cohorts

We assessed the consistency of lead SNP effects between the two definitions of idiopathic infertility, i.e. by excluding known causes such as anovulation, anatomical, PCOS, endometriosis, and uterine fibroids (F-EXCL) and by inclusion of a diagnostic code for idiopathic infertility (F-INCL). Two lead SNPs were shared between these definitions (i.e. rs61768001 mapped to *WNT4* and rs17378154 mapped to *PKHD1L1/EBAG9*, which are discussed in the Main text. Another two lead SNPs associated with F-INCL reach nominal significance (P<2.27E-03, Bonferroni adjustment for 22 unique lead SNPs tested across all categories) for F-EXCL, i,e, rs74156208 mapped to *TMEM26* and rs192462512 mapped to *BCOR*. All lead SNPs for F-EXCL and F-INCL are directionally consistent across both categories (Supp. Figure 19).

All but four of the reported lead variants for infertility were present in at least two cohorts (Supp. Table 3). Among these the shared SNPs were three variants for F-ANOV, two variants for F-INCL, and one variant for M-ALL that were only present in FinnGen and EstBB; as the Finnish population underwent a recent bottleneck event and is genetically connected to Estonians, common variants in these two populations may not be represented in other European-ancestry cohorts[7]. Eighteen Twenty of 21 lead variants present in at least two cohorts show no evidence for heterogeneity in effect estimates across cohorts (all P_{het} > 0.0025, Bonferroni adjustment for 25 unique variant-trait pairs tested) (Supp. Table 3). , and the remaining have consistent effect directions in all of the studies in which they are present rs9696009 (average MAF=7.77%, nearest gene *DENND1A,* F-ANOV OR (95% CI)=1.21 (1.14-1.29), P=6.87E-10) is the only variant to show significant heterogeneity (P_{het}=0.00127) in effect estimates across studies, but nonetheless has consistent effect direction across all four cohorts where the variant was present, with effect sizes ranging from OR (95% CI)=1.06 (0.897-1.26) in G&H to OR (95% CI)=1.57 (1.30-1.90) in the Danish sample.(Supp. Table 3).

We ensured that our hormone GWAS meta-analysis results were robust to the inclusion of summary statistics from publicly available datasets. The majority of lead variants associated with Oestradiol-F (2/2), Oestradiol-M (5/62/4), Testosterone-F (146/153), and Testosterone-M (230/243228/261), were recovered at genome wide significance when publicly available datasets were excluded, and the small number of remaining variants were still nominally associated at P<5E-05. There is also no heterogeneity between effect size estimates from meta-analyses with and without public summary statistics (heterogeneity P_{het}>0.1500.01 at all lead variants across all strata) (Supp. Figure 5). Finally, as with the infertility meta-analyses, all reported lead variants from the reproductive hormone meta-analyses were present in at least two of the cohorts examined and the majority (81%) show no evidence for heterogeneity in effect estimates across cohorts (P_{het}>0.05).

Relationships between age at menopause, infertility, and reproductive hormones

As age at menopause is considered a proxy of ovarian reserve, and AMH is associated with menopause timing[82], we assessed the genetic relationships between age at menopause, female infertility, and AMH. There was nominally significant (P<0.05) positive genetic correlation between F-ANOV and AMH (rG (SE)=0.748 (0.301), P=0.0131) and between F-ANOV and age at menopause (rG (SE)=0.164 (0.0789), P=0.0382). We also tested for colocalisation[134] of genetic signals among these three traits and identified statistically significant overlap (posterior probability (PP) of shared causal SNP=100%) between F-ANOV and age at menopause at the *INHBB* locus. However, this locus is not shared with AMH (PP=0.363%), as there is no evidence for an AMH association at the *INHBB* locus (minimum P=0.119). The association between increased risk of anovulatory infertility and older age at menopause at this locus is likely driven by inhibin B regulation, itself a marker of ovarian reserve.

As detailed in the Main text, clinical measurements of the gonadotropins FSH and LH may have been used to diagnose premature menopause. The lead SNPs for these hormones from our reported meta-analysis did not differ in their effect sizes between GWASs based solely on the 5,949 women in UKBB with age at menopause >45 years (i.e. women who did not experience early menopause) as compared to GWASs in all 20,749 women in UKBB with measurements of these hormones (heterogeneity P> 3.85E-03, Bonferroni adjustment for 13 SNP-trait pairs tested, Supp. Table 25). However, as we may have missed sub-genome wide significant signals for these hormones, we also assessed the heterogeneity in variant effects among SNPs that reached P<1E-06 for either the full set of women in UKBB or the subset that did not undergo early menopause (SNPs were distance-pruned, resulting in 109 independent SNP-trait pairs tested). Four SNPs show significant differences (P<4.59E-04, Bonferroni adjustment for 109 tests) in effects between these two samples, all of which only reach significance in the subset of women who did not go through early menopause, and have between 5.98-11.0x larger effects in these women than the full dataset (Supp. Table 25). rs78114522 (MAF=1.77%, intron in *ZNF148*) is associated with FSH levels in women with menopause age >45 years (beta (SE)=-0.501 (0.104), P=1.43E-06); *ZNF148* is a transcriptional repressor expressed in the thyroid gland. Three variants are only associated with LH in the subset of women who undergo menopause at age >45 years: rs140409 (MAF=47.5%, beta (SE)=0.108 (0.0224), P=1.60E-06) is near *GGTLC2*, whose deficiency is associated with altered circulating FSH, impaired folliculogenesis, absent corpus luteum and tertiary ovarian follicles, and oocyte degeneration in mice[8] and with elevated thyroid levels in humans[9]; rs113176873 (MAF=1.87%, beta (SE)=0.362 (0.079), P=4.53E-06) is near *PTK2B*, whose deletion improves folliculogenesis and fecundity, mediated by the gonadotropin-stimulating ERK cascade, in mouse models[10]; and rs61412562 (MAF=36.1%, beta (SE)=-0.0966 (0.0217), P=8.13E-06) an intronic variant in *ZNF234.* While these are promising candidate genes to understand the relationships between gonadotropins and female infertility, none of the variants noted reach genome-wide significance in our analyses and must be replicated.

Genome-wide selection scans for infertility-associated variants

We did not observe any GWS variants for infertility in 54 genomic regions under historical directional selection (50,000 years or 10,000 years) (Supp. Table 8). We also did not find significant genome-wide directional selection against infertility when SDS were aligned to the

infertility-risk increasing allele: the mean genome-wide trait-SDS is not significantly different from 0 for any category of infertility (all *P*>0.05).

We observed signatures of balancing selection, which maintains multiple alleles in the population through mechanisms such as heterozygote advantage or time-varying fitness[11,12], at the female infertility loci *GREB1* (StdB2 in the 98.6th-99.4th %ile of SNPs within 10kb of a GWAS Catalog variant) and *INHBB* (98.5th %ile), and the male infertility locus *PCDH15* (98.7th %ile); however, variants at these loci with high probability of association with infertility did not have high balancing selection scores (Supp. Figure 2 and Supp. Table 7).

Classification of genetic variants associated with hormones

We classified lead variants as entirely novel hormone associations by defining a protocol based on linkage disequilibrium (LD) and conditional independence from published SNPs associated with any of 28 reproductive hormones in the GWAS Catalog[6] (see Methods for detailed classification protocol).

We report lead SNPs independent of previously published hormone variants in the *HELQ* locus for FSH-F (rs4235062: MAF=47.5%, β=0.046 (0.0065), *P*=1.50E-12), *TMEM150B* locus for FSH-F (rs28875253: MAF=40.9%, β=-0.0599 (0.0061), *P*=9.90E-23) and LH-F (rs11668309: MAF=37.7%, β=0.0519 (0.0071), *P*=3.91E-13), and in the *SOX15-SAT2* locus for oestradiol-F (rs3933469: MAF=27.1%, β=0.0363 (0.0051), *P*=1.02E-12) (Supp. Table 10).

We replicated genetic variants associated with reproductive hormones in or near genes that encode the hormone subunits themselves, such as *FSHB,* which encodes the FSH beta subunit (rs11031005, FSH-F β (SE)=-0.104 (0.0092), *P*=1.08E-29 and FSH-M β=-0.206 (0.0263), *P*=3.75E-15), and *LHB*, which encodes the LH beta subunit (rs753307, LH-F β (SE)=0.0605 (0.0072), *P*=4.53E-17) (Supp. Table 10). We also replicated associations near genes encoding enzymes for steroid hormone metabolism, such as *CYP3A7*, whose encoded enzyme metabolises a precursor of oestrogen (rs45446698, Oestradiol-F β=-0.0782 (0.0114), *P*=8.26E-12), and *HSD17B13*, whose encoded protein catalyses the oxidation of oestradiol[13] (rs13133311, Testosterone-M β=-0.0307 (0.00270), *P*=2.84E-29) (Supp. Table 10).

Sex-specific genetic architecture of testosterone

Only 9.80% (of 153 total) lead variants for testosterone in females and 5.75% (of 261 total) lead variants for testosterone in males reach genome-wide significance in both sexes; and 45.9% of variants have opposing directions of effect in men and women (Supp. Figure 6). Indeed, we found no significant genetic correlation between testosterone in men and women (r_g (SE)=0.0361 (0.0428), *P*=0.399), which is the lowest cross-sex genetic correlation among all traits in the UK Biobank.

Over a third (39.7%) of lead variants identified in either of the sex-specific analyses for testosterone had significant sex-differential effects (sex heterogeneity *P*<0.05/399, Bonferroni-adjusted for number of unique lead variants tested) (Supp. Figure 6). Bernabeu *et al.* (2021) calculated the genetic correlation between sexes for 161 traits in the UK Biobank[14]. Testosterone (cross-sex rG=3.61%) has the lowest cross-sex genetic correlation

across all these traits; the next lowest are for self-reported ear/nose/throat disorders (rG=22.8%) and disorders of the urinary tract (rG=40.4%).

Bernabeu *et al.* (2021) also reported the number of sex-dimorphic lead SNPs (sdSNPs, defined as having sex-heterogeneity P<1E-08 for difference between male and female effect sizes) across 530 traits in the UK Biobank[14]. We followed the same protocol to define sdSNPs for testosterone. 104 traits (20%) assessed by Bernabeu *et al.*[14] had at least one autosomal sdSNP; and only waist-to-hip ratio (100 sdSNPs) had more sdSNPs than testosterone (73 sdSNPs). The trait with the most sdSNPs after testosterone was height (18 sdSNPs) (Supp. Figure 16). Testosterone also had the most X-chromosome sdSNPs (6) of any trait evaluated in UK Biobank (Supp. Figure 16).

The genome-wide SNP-based heritability of total testosterone was 1.5x higher in men (15.67%, SE=1.79%) than in women (9.87% (1.00%)) (Supp. Table 6). The phenome-wide genetic correlation landscape of testosterone, assessed across 703 heritable phenotypes in UKBB, was also substantially different between the sexes (Supp. Figure 7 and Supp. Figure 8, phenome-wide significant at P<4.90E-05). Total testosterone was correlated with obesity-related phenotypes in opposite directions in women (positive) and men (negative), including body fat percentage (r_g-female (F)=0.172 (0.0212), r_g-male (M)=-0.320 (0.0247)), body mass index (r_g-F=0.170 (0.0195), r_g-M=-0.302 (0.0207)), waist circumference (rG-F=0.163 (0.0205), r_g-M=-0.344 (0.0215)), and hip circumference (rG-F=0.148 (0.0217), rG-M=-0.263 (0.0226)). Instead, Testosterone-M was positively correlated with general well-being, as indicated by: not being on medication for cholesterol, blood pressure, diabetes, etc. (r_g=0.302 (0.0307)), recent strenuous physical activity (r_g=0.190 (0.0340)), and forced vital capacity (r_g=0.148 (0.0226)) (Supp. Figure 8).

Tissue- and cell-type enrichment of infertility and reproductive hormones

The heritability of testosterone in women is enriched in the adrenal gland (P=1.03E-03) and hepatocytes (P=9.36E-04); but only the latter is enriched for the heritability of testosterone in men (P=3.61E-04), as is the liver more broadly (P=1.16E-06) (Supp. Figure 10, stratified LD-score regression performed across 205 tissues and cell-types from the Genotype Tissue Expression (GTEx) Project database[4] and the Franke lab single-cell database[5]). Finally, although testosterone regulates several traits hypothesised to be under sexual selection and may be under selection itself[15], we do not find significant genome-wide directional selection for testosterone in men or women (mean genome-wide trait-SDS is not significantly different from 0, both P>0.05) (Supp. Text).

As GTEx datasets do not comprehensively represent female reproductive tissues of interest, particularly the ovary, we curated gene sets for ovarian cell types from publicly available single-cell gene expression databases[16,17]. At FDR<5%, we find enrichment of testosterone-F across a range of ovarian endothelial and immune cell types, as well as granulosa cell progenitors (P=0.0106), granulosa cumulus cells (P=0.0113), and theca cells (P=0.00176) (Supp. Figure 10).

We did not find significant enrichment of infertility heritability in any of the 205 tissues and cell-types from the GTEx project database[4] and the Franke lab single-cell database[5].

Directional selection at testosterone-associated loci

We found evidence of recent directional selection, as measured by extreme SDS (in the lowest 0.25^{th} %ile and highest 99.75^{th} %ile), at four loci associated with testosterone (Supp. Figure 9). We observed negative selection of the testosterone-M-increasing allele at *DNAL1*, where we found that variants with higher probability of association with testosterone also had more negative selection scores. While we observed positive selection of the testosterone-increasing alleles at three loci: female-specific associations at the *CXCR4* and *SLC17A1* loci, and male-specific associations at the *SRD5A2* locus, the relationship between strength of variant association with testosterone and selection score was not consistent.

GWASs for FSH and LH not biased by diagnostic measurements of hormones

Clinical measurements of FSH and LH may be used to diagnose premature menopause[18], so our hormone GWASs based on these measurements may have captured signals for menopause timing (12 lead variants for FSH and five for LH have previously been reported for associations with age at menopause). We found a significant (two-tailed *t*-test P<0.05), but small (0.2 years) difference between self-reported age at menopause in women with absence of clinical measurements of FSH or LH (mean (SE) age at menopause = 49.7 (0.0135) years) and those with measurements of FSH (mean=49.5 (0.0583) years, P=0.0141) and LH (49.5 (0.0695) years, P=0.00155) (Supp. Figure 18). This potential ascertainment bias did not appear to affect GWAS results – all lead SNPs for FSH and LH had consistent effects in the subset of 5,949 women in UKBB known to have undergone menopause at age >45 years (that is, they did not experience early menopause) as in the full dataset of 20,749 women without filtering on menopause status (heterogeneity P>3.85E-03, Bonferroni adjustment for 13 SNP-trait pairs tested, Supp. Table 25, Supp. Text).

Local genetic correlations between infertility, reproductive conditions, hormones, and obesity

Across the 2,495 genomic regions tested for bivariate local genetic correlations between 42 pairs of traits (3 categories of infertility versus 5 reproductive conditions, 4 reproductive hormones, and 5 obesity-related traits), we found 56 regions with significant local r_g at FDR-adjusted P<0.05, none of which reached Bonferroni-adjusted significance (Figure 4A, Supp. Figure 17, Supp. Table 22).

Female infertility was locally correlated with testosterone at seven loci (6 -ve/1 +ve), TSH at four loci (3 -ve/1 +ve), FSH at two loci (1 -ve/1 +ve), and AMH at one locus (-ve) (all FDR-adjusted P<0.05, Supp. Figure 17 and Supp. Table 22), suggesting complex genetic relationships among these traits that may not be captured by genome-wide correlations. However, as these associations do not reach significance after Bonferroni adjustment for multiple testing, further statistical and functional follow up will be necessary to confirm local bivariate relationships.

Multiple obesity-associated traits were locally correlated with female infertility (FDR P<0.05) at three regions: chr6:131457165-132890694, where the positive association of hip circumference with F-INCL remained even upon accounting for BMI and body fat percentage (P=0.0467), chr17:47516225-48722590, where the negative association of waist

circumference with F-ANOV remained upon conditioning on BMI, body fat percentage, and hip circumference (P=0.0152), and chr10:71672998-72659223, where the positive correlation between F-ANOV and body fat percentage, hip circumference, and waist circumference does not appear to be driven by any one trait alone (all P>0.05, Supp. Table 22). These results indicate value in exploring the local genetic effects of body fat distribution on infertility, but must be interpreted with caution as they do not reach significance after Bonferroni correction for multiple testing.

Finally, there are three genomic regions where F-ANOV is genetically correlated with both a reproductive disorder and obesity. At the chr5:166863414-168342743 region, the negative correlation between F-ANOV and heavy menstrual bleeding remains upon adjustment for BMI (P=0.00582) and similarly at the chr9:126092932-127184582 locus, the positive correlation between F-ANOV and PCOS remains upon adjustment for BMI (P=0.0253), suggesting that these disorders affect fertility independent of obesity. However, at the chr17:7264459-8554763 locus, F-ANOV is independently positively correlated with uterine fibroids (local r_g (SE)=0.566 (0.0928), P=2.70E-08), waist circumference (r_g=0.491 (0.151), P=1.84E-03), and testosterone (r_g=-0.490 (0.114), P=1.43E-05), indicating complex pleiotropic relationships between infertility, reproductive disease, hormones, and obesity at this locus.

Polygenic overlap between infertility, reproductive conditions, hormones, and obesity

We used MiXeR[19] to calculate the extent of polygenic overlap, irrespective of genetic correlation, between the 42 pairs of traits described above (3 categories of infertility versus 5 reproductive conditions, 4 reproductive hormones, and 5 obesity-related traits). All phenotypes considered had sufficient power to justify the MiXeR model over LDSC, as measured by positive Akaike Information Criteria (AIC) (Supp. Table 23). Genome-wide genetic correlation was equivalent between MiXeR and LDSC, with P-value for heterogeneity in estimates between the two methods being >0.05 between all pairs of traits tested (Supp. Table 24).

We only observed substantial polygenic overlap (>20%) between infertility and the female reproductive disorders discussed in the main text: endometriosis (up to 53.5% of causal variants shared), heavy menstrual bleeding (up to 35.2%), and uterine fibroids (up to 26.4%) (Supp. Table 24). There was positive correlation of effect sizes between infertility and endometriosis in the shared polygenic component (F-INCL: 563 shared causal SNPs of 1,053 total, rho (SE)=0.957 (0.0649) in the shared polygenic component; F-ALL: 721/1,448, rho (SE)=0.544 (0.298); F-ANOV: 562/1,150, rho (SE)=0.141 (0.0793)). Similarly, we found that between 23.7%-26.4% of causal SNPs involved in infertility and uterine fibroids were shared, with genetic correlation in the shared component ranging from rho=0.431 (SE=0.183) with F-ANOV to rho=0.738 (0.112) with F-ALL. PCOS did not have substantial overlap with any of the infertility traits tested (4.08% with F-ALL to 10.9% with F-ANOV), but the genetic correlation between infertility and PCOS in the shared component was strongly positive (0.544-0.712).

We found no genome-wide correlation between dizygotic twinning and all definitions of infertility except F-ANOV, and limited polygenic overlap (13.3%-19.1%). However, we observed strong positive correlation between twinning and F-ALL (rho=0.752 (0.310)) and

F-INCL (rho=0.921 (0.146)) in their shared polygenic components, and local correlation in the chr22:44409534-45170649 region (F-ALL local r_g= 0.632 (0.163), *P*=2.43E-04) (Figure 4B, Supp. Table 24). These local effects may arise from the residual influence of age at some loci – although fertility in women declines with age, the propensity for double ovulation also increases with age[20].

There was limited polygenicity for reproductive hormones FSH (polygenicity (SE)= 0.0031% (0.00347), number of causal SNPs (Nc) (SE)= 99 (111)) and AMH (polygenicity=0.00389% (0.00389), Nc=124 (124)) (Supp. Table 23). We found that nearly all causal variants influencing these hormones also appeared in the causal components of female infertility (Supp. Table 24). However, the more highly polygenic hormones testosterone (polygenicity=0.0243% (0.00468), Nc=773 (149)) and TSH (polygenicity=0.0137% (0.000846), Nc=436 (27)) had a large proportion of unique SNPs not shared with infertility (between 19.6%-45.9%), indicating that while some of the genetic mechanisms influencing hormone levels may also result in fertility issues, others only affect reproductive hormones without influencing fertility.

Obesity-associated traits were highly polygenic (between 4,050 (SE=240) causal SNPs for comparative body size at age 10 years to 11,000 (406) for body fat percentage) (Supp. Table 23). A substantial proportion of variants causal for infertility were therefore also in the polygenic components for obesity, such as body fat percentage (683/880 F-ANOV SNPs, 440/785 F-INCL SNPs), waist circumference (566/880 F-ANOV SNPs, 432/785 F-INCL SNPs), and BMI (592/880 F-ANOV SNPs, 393/785 F-INCL SNPs) (Supp. Table 24). The lack of significant local correlations between these traits indicates that further work will be necessary to unpick the shared and distinct genetic contributions to obesity and infertility.

Mendelian randomisation analyses

Although the effect of FSH on female infertility appears to be driven by a single outlier variant (rs150206026), leave-one-out sensitivity analyses indicate that the MR effect remains consistent upon excluding this variant (MR *P*<0.00158) (Supp. Figure 15).

Reproductive hormone-associated genes implicated by gene burden analyses

Aside from the novel *HSD11B1* finding for testosterone-F, we report eight six additional genes associated with testosterone-F at exome-wide significance (Cauchy *P*<5E-06) that have not previously been implicated in GWASs, including those expressed in the ovaries (*ANAPC2TNXB*) and adrenal glands (*GPC2*), and genes associated with metabolism (*PDE3B*, *TAP2*, *NOTCH4*, and *ZKSCAN1*) (Supp. Figure 11 and Supp. Table 14). The testosterone-F association with *TNXB* replicated nominally in deCODE (*P*=0.041); rare variants in *TNXB* cause Ehlers-Danlos syndrome[21], a collection of disorders affecting connective tissues that has previously been linked to sex hormone dysregulation[22]. We also identified, for the first time, the *ELFN2* association with testosterone-M (Supp. Figure 11 and Supp. Table 14). Finally, across the reproductive hormones studied here, we replicated genes that have previously been reported to carry either rare or common variants associated with: FSH-F (genes *CHEK2* and *DCLRE1A*), Oestradiol-F (gene *SHBG*), Testosterone-F (18 12 genes, including two that are novel for testosterone but have known estrone associations), and Testosterone-M (22 16 genes, including eight three that are novel for

testosterone but have known sex-hormone binding globulin associations) (Supp. Figure 11 and Supp. Table 14).

Concordance between rare and common genetic architecture for testosterone

23 of 29 genes associated with testosterone in our gene burden analyses carried rare variants that were present in our EUR-ancestry GWAS meta-analyses excluding UKBB participants. 21 of the 23 (91.3%) had the same direction of effect across analyses (Supp. Table 15). Three rare variants associated with testosterone-F (in genes *ZKSCAN1*, *ZAN*, and *SHBG*), and one rare variant associated with testosterone-M (in *SHBG*) replicated in the GWAS meta-analysis at P<2.17E-03 (Bonferroni adjustment for 23 variants tested); an additional six variants replicated at P<0.05 (Supp. Table 15).

In the majority (8374%) of genes with testosterone-associated rare variants, the effect of the rare variant remains upon conditioning for nearby common variants (within 500 kb), and the rare variant has a larger absolute effect size on the trait than do any common variants in the genethe lead common variant. For example, a rare missense variant (chr17:7631360:C:T, MAF=0.612%) in *SHBG* is associated with large effects on testosterone-M (β=-0.743, P=1.69E-291), testosterone-F (β=-0.121, P=7.83E-09), and oestradiol-F (β=-0.259, P=1.03E-09); the common variants in *SHBG*, have effect sizes smaller by a factor of 7.5 (largest absolute effect of a lead common variant in *SHBG* on testosterone-M: β=-0.11397, P=3.46E-820, testosterone-F: β=-0.03214, P=4.006.55E-22, and oestradiol-F: β=-0.0354, P=2.671.18E-11) (Supp. Table 15). Three rare variants on chr7 associated with testosterone-F (in genes *ZKSCAN1*, *GPC2*, and *STAG3*) are no longer significant (all P>1E-07) after conditioning upon nearby common variant rs17250196 (MAF=5.18%, β=-0.129, P=4.90E-84). Similarly, five rare variants on chr17 associated with testosterone-M (in genes *SLC2A4*, *NLGN2*, *SPEM1*, *SPEM2*, and *TMEM102*) do not remain significant after conditioning upon rs12946520 (MAF=36.3%, β=0.113, P=0); and the effect of a rare variant in the androgen receptor gene on chrX may similarly be explained by two nearby common variants, rs139478468 (MAF=7.89%, β=0.0588, P=3.26E-55) and rs7052964 (MAF=18.8%, β=0.0403, P=1.65E-53).

Increased risk of infertility in individuals carrying rare testosterone-associated variants

In addition to the *GPC2* variant associated with lower testosterone and higher risk of infertility in women, we find a nominally significant association between a testosterone-lowering missense variant in *ZAN* and female infertility (chr7:100766559:A:G, F-ALL OR=1.37 (0.949-1.98), P=0.0463) (Figure 6B). *ZAN* is expressed in the testis and knockout mice display impaired sperm binding to the zona pellucida[23]. In men, a testosterone-lowering damaging missense variant in *HNF4A* (chr20:44413714:C:T OR=17.6 (2.26-137.07), P=3.08E-03) was associated with increased odds of infertility (Figure 6B).

Supplementary Discussion

Although infertility is a highly prevalent condition, the complex and heterogeneous causes of this condition remain poorly understood[24]. The case proportion of male and female infertility was between 0.3% and 1.4% in UKBB, which may reflect: (1) the biases of UKBB for healthy participants[25], (2) participants who were born between 1950-70 and thus reached reproductive age when infertility treatments were not widely available[26], and (3) the separation of fertility treatment records from other medical databases in the UK[26]. On the other hand, the Estonian and Danish data more closely reflect the population prevalence of infertility (between 7.0% to 13.2%) - the Estonian biobank is currently recruiting participants and is reflective of the current age distribution in Estonia[27], with recent infertility diagnoses included in the dataset, while the Copenhagen Hospital Biobank consists of patients with blood draws in Danish hospitals[28] and is thus more likely to capture individuals who have interacted with the healthcare system, which is genetically correlated with infertility in our study.

While we did not identify any genetic loci for infertility that have previously been reported for educational attainment or behavioural traits, we observed genetic correlations between female infertility and traits correlated with educational achievement[29,30], which is itself associated with delayed age at first birth[31]. There may still be residual confounding of age in our phenotype for female infertility of all causes. Indeed, this residual confounding is also highlighted by the positive local correlations between dizygotic twinning and female infertility, which may arise from the propensity of double ovulation to increase with age as a strategy to maximise reproductive success.

FSH and LH measurements were historically used in the UK to clinically diagnose early menopause[18]. While we found no evidence for heterogeneity in variant effect sizes on FSH and LH between women reporting “regular” age at menopause (>45 years) compared to all women in UK Biobank, we identified sub-genome-wide significant signals (P<1E-06), with effects on gonadotropin pathways and folliculogenesis, which only had effects in the subset of women who went through menopause after 45 years of age. While this needs replication in larger studies, our work suggests the importance of stratifying by menopause status for genetic studies of reproductive hormones. In the future, longitudinal GWASs that can incorporate mean and variance of hormone levels over the menstrual cycle, or phenotypes that calculate ratios between various hormones over time, will likely reveal fundamental biology that is missed by the broad-stroke assessments in this study.

The testes and ovaries were not significantly enriched for the heritability of infertility or testosterone, despite being reproductive organs that are major sites for testosterone production[32,33]. However, neither organ is disaggregated into tissues or cell types in the GTEx database, so gene expression profiles may not capture cell-type specific effects. Indeed, by analysing three publicly available ovarian transcriptomics datasets, we found enrichment of testosterone heritability in the androgen-secreting theca cells and androgen-responsive granulosa cells of the ovary[34–36], and female infertility in ovarian stromal cells. Although there are several causal roles hypothesised for stromal dysfunction in infertility, such as impaired folliculogenesis[37], restricted blood flow[38], and ovarian scarring[39], more work is needed to robustly replicate these findings. In general, more functional studies

of gonadal cell types, in both men and women, are needed to enable a mechanistic understanding of the genetic variation associated with reproductive hormones and infertility.

Rare protein-coding variants captured in whole exome sequencing studies are valuable in revealing the underlying biology of phenotypes, as they directly implicate effector genes[40]. Indeed, in our UK Biobank-based WES analyses, we found that several enzymes involved in steroid hormone synthesis and metabolism, such as those in the hydroxysteroid dehydrogenase (HSD) family[41], carry rare variants that affect reproductive hormone levels. We also displayed concordance between the testosterone-altering effects of common and rare variation in a gene, mirroring the inverse relationship between allele frequency and effect size that has been observed across several other phenotypes[42].

Testosterone is among the most sexually dimorphic phenotypes in humans and has a corresponding dimorphic genetic architecture. Previous studies[43] and ours estimate the genetic correlation between female-specific and male-specific testosterone to be negligible (between 0 to 3%), highlighting the importance of disaggregating both genetic and phenotypic analyses by sex. We also found opposing associations of total testosterone with adiposity in the two sexes - testosterone in women is genetically correlated with higher overall and abdominal obesity, as measured by BMI, WC and HC, body fat percentage, fat mass, etc., but these were all negatively correlated with total testosterone in men. These sexually dimorphic genetic correlations reflect reported observational and causal associations in the literature[44,45], and may be related to the hormonal activity of adipose tissue, which metabolises steroid hormones and is differently distributed in men and women[46]. As testosterone and sex-hormone binding globulin are thought to partially mediate the deleterious cardiovascular consequences of increased visceral adipose tissue mass in men and post-menopausal women[47,48], understanding the shared genetic aetiology of testosterone and adiposity could lead to novel insights and treatments for cardiovascular disease.

Indeed, despite our best efforts to encompass heterogeneity in the causes and effects of infertility across the globe, we remained under-powered to perform sub-type specific GWASs for different categories of male infertility, where diagnosis and treatment lags behind female infertility[49]. Further, while the all-ancestry GWAS meta-analyses included one cohort of South Asian ancestry (Genes and Health) for infertility meta-analyses, and individuals of African, East Asian, and South Asian ancestries in the UKBB for hormone meta-analyses, over 90% of participants in our GWASs were of European ancestry. Even within the European-ancestry analyses, we demonstrated the importance of diverse population representation for biological insights in genetic studies. We identified six infertility-associated variants that were common in the Finnish and Estonian populations (MAF>1%), but not present in any of the other European cohorts, some of which may have immediate implications for fertility - such as the male infertility variant near *ENO4*, a gene expressed in the testis and involved in sperm motility[50,51]. Increasing the ancestral diversity in GWASs will not only be of great benefit to populations with ancestry-specific variants that affect fertility and reproductive hormone levels, but may also reveal shared novel biology.

Supplementary Methods

Study populations for GWAS meta-analysis

UK Biobank. The UK Biobank (UKBB) is a prospective UK-based cohort study with approximately 500,000 participants aged 40–69 years at recruitment for whom a range of medical, environmental, and genetic information is collected[52]. Genotyping using two custom Affymetrix arrays, initial genotype quality control (QC), and imputation to the hg19 reference genome were performed by UKBB[53]. Sample ancestry was genetically ascertained as outlined below; in total, we retained 487,202 individuals of European ancestry for case-control analyses (Supp. Table 2), and 394,378 individuals of European ancestry, 10,548 individuals of African ancestry, 1,079 individuals of East Asian ancestry, and 7,019 individuals of South Asian ancestry with at least one hormone measurement for quantitative trait analyses (Supp. Table 9). GWASs in the UKBB were additionally adjusted for one-hot encoding of data provider or assessment centre, genotyping batch, genotyping array, and the first 21 genetic principal components (PCs).

We assigned sample population labels by training a random forest (RF) classifier using the 1000 Genomes ‘super-population’ labels. We first ran principal components analysis (PCA) on unrelated individuals in the 1000 Genomes project dataset, subset to LD-pruned autosomal variants. Samples in the UKBB genotyping data are projected onto this PCA space, ensuring that we correctly account for shrinkage bias in the projection[54,55]. Next, we used the ‘super-population’ labels (AFR=Africans, AMR=Admixed Americans, EAS=East Asians, Europeans=EUR, South Asians=SAS) of the 1000 Genomes dataset to train a RF classifier, using the randomForest (4.6) library in R[56], and predicted the super-population for each of the UKBB samples. Samples with classification probability >0.99 were retained for downstream analysis.

We carried out a sensitivity analysis for GWASs of FSH and LH, whose measurements may be used to diagnose premature menopause, by identifying a subset of 5,949 women of non-Finnish European ancestry in the UK Biobank with FSH or LH measurements and with self-reported age at menopause >45 years. We tested for heterogeneity in meta-analysis lead SNP effect sizes between this subset of women and all 20,749 women with an FSH or LH measurement. We also identified 109 lead SNPs by distance-based pruning of SNPs with P<1E-06 (as we may have missed sub-genome-wide significant signals above) associated with FSH or LH in either the full dataset or the subset of women without premature menopause to assess heterogeneity between the two sets.

UK Biobank has approval from the North West Multi-centre Research Ethics Committee (MREC). This research was conducted using the UK Biobank Resource under Application Number 11867.

Avon Longitudinal Study of Parents and Children. The Avon Longitudinal Study of Parents and Children (ALSPAC) is a longitudinal population-based study that recruited 13,761 pregnant women resident in the South West of England, with expected delivery dates between 1 April 1991 and 31 December 1992, who have continued to be followed up over the last 32 years[57]. GWASs were performed on reproductive hormone values measured in follow-up assessments (not during pregnancy) from approximately 3,000 mothers with linked

genetic data. Genome-wide genotyping was performed using the Illumina HumanHap550 quad chip genotyping platform at 477,482 markers and imputed to the hg19 reference genome using the Haplotype Reference Consortium (HRC) panel[57,58]. GWASs in ALSPAC were performed using the BOLT-LMM software[59] and additionally adjusted for the first 10 genetic PCs.

Ethical approval for the study was obtained from the ALSPAC Ethics and Law Committee and the Local Research Ethics Committees.

Copenhagen Hospital Biobank & The Danish Blood Donor Study. The Copenhagen Hospital Biobank (CHB)includes samples drawn for blood type testing or antibody screening from more than 500,000 hospitalised patients and outpatients in Danish hospitals in the Capital Regions[28]. The Danish Blood Donor Study (DBDS) is a prospective cohort study of 163,000 healthy blood donors. Genotyping in both cohorts was performed by deCODE Genetics using the Illumina Global Screening Array (GSA) microchip at 660,000 markers and imputed to the hg38 reference genome using a custom panel[28,60]. The GWASs in CHB/DBDS were performed with SAIGE v1.18 were additionally adjusted for the first 10 genetic PCs.

CHB is classified as a 'biobank for future research'. It is part of the Danish National Biobank and has been approved by the Danish Data Protection Agency (general approval number 2012-58-0004, and local number: RH-2007-30−4129/I-suite 00678). DBDS was approved by the Central Denmark (1–10-72–95-13) and Zealand (SJ-740) Regional Committees on Health Research Ethics and the Data Protection Agency (P-2019–99).

deCODE. 173,025 Icelanders that have been genotyped using Illumina SNP chips were long-range phased[61] and variants identified in the WGS of 63,460 Icelanders were imputation into chip-typed individuals and their close non-chip-typed family members[62]. The sequencing was done using Illumina standard TruSeq methodology. Only samples with a genome-wide average coverage of 20X were considered. Autosomal SNPs and INDEL's were called using Graphtyper version 1.4.5. Variants that did not pass quality control were excluded from the analysis. Information about haplotype sharing was used to improve variant genotyping, taking advantage of the fact that all sequenced individuals had also been chip-typed and long-range phased.

Information on the fertility phenotypes was extracted from information from phenotype records from questionnaires and healthcare centres. Logistic regression analysis assuming a multiplicative model was used to test for association[62] between fertility cases/control phenotypes, additionally adjusted for year of birth and county of origin. For hormone biomarkers, values were additionally adjusted for measurement centre and county of origin, standardised using an inverse-normal transform and tested for association with sequence variants using a generalised linear model. Any inflation in the test statistic due to population structure and relatedness of the participants was adjusted for using LD score regression.

The deCODE study was approved by the Icelandic National Bioethics Committee (VSN-15-169).

Estonian Biobank. The Estonian Biobank (EstBB) is a population-based biobank of Estonia, reflecting the age, sex, and geographic distribution of the population, with a cohort size of 200,000 individuals (approximately 20% of the nation) with EHR linkage[27]. Individuals were genotyped using the Illumina Global Screening Array (GSA) microchip at 700,000 markers and imputed to the hg19 reference genome using a custom panel[63]. GWASs in EstBB were performed using the REGENIE software v3.0.3[64] and additionally adjusted for the first 10 genetic PCs.

The Estonian Biobank (EstBB) is under ethical approval 1.1-12/624 from the Estonian Committee on Bioethics and Human Research (Estonian Ministry of Social Affairs).

FinnGen. The FinnGen study is a large-scale genomics initiative that has analyzed over 500,000 Finnish biobank samples and correlated genetic variation with health data to understand disease mechanisms and predispositions. The project is a collaboration between research organisations and biobanks within Finland and international industry partners. FinnGen Data Release R10 was used in the present study. Individuals were genotyped with Illumina and Affymetrix chip arrays, and genotype data were imputed using the population-specific SISu v4.2 imputation reference panel of 8,554 whole genomes. GWAS was performed using the REGENIE software v2.2.4[64] and additionally adjusted for the first 10 genetic PCs and genotyping batch.

Participants in FinnGen provided informed consent for biobank research on the basis of the Finnish Biobank Act. Alternatively, separate research cohorts that were collected before the Finnish Biobank Act came into effect (in September 2013) and the start of FinnGen (August 2017) were compiled based on study-specific consent and later transferred to the Finnish biobanks after approval by Fimea, the National Supervisory Authority for Welfare and Health. Recruitment protocols followed the biobank protocols approved by Fimea. The Coordinating Ethics Committee of the Hospital District of Helsinki and Uusimaa approved the FinnGen study (protocol number HUS/990/2017). The FinnGen study is approved by the Finnish Institute for Health and Welfare (approval number THL/2031/6.02.00/2017, amendments THL/1101/5.05.00/2017, THL/341/6.02.00/2018, THL/2222/6.02.00/2018, THL/283/6.02.00/2019 and THL/1721/5.05.00/2019), the Digital and Population Data Service Agency (VRK43431/2017-3, VRK/6909/2018-3 and VRK/4415/2019-3), the Social Insurance Institution (KELA) (KELA 58/522/2017, KELA 131/522/2018, KELA 70/522/2019 and KELA 98/522/2019) and Statistics Finland (TK-53-1041-17).

Genes and Health. The Genes and Health study (G&H) is a longitudinal prospective cohort of participants of British Bangladeshi and Pakistani origin recruited from East London, Bradford, and Manchester, with EHR linkage[65]. Participants were genotyped using the Illumina GSA Multi-Disease GSAv3EAMD chip and imputed to the hg38 reference genome using the TOPMED reference panel[65,66]. GWASs in G&H were performed using the REGENIE software[64] and additionally adjusted for the first 20 genetic PCs.

A favourable ethical opinion for the main Genes & Health research study was granted by NRES Committee London - South East (reference 14/LO/1240) on 16 Sept 2014. Queen Mary University of London is the Sponsor.

Million Veteran Program (MVP) (publicly available summary statistics). We downloaded the multi-ancestry meta-analysis summary statistics released by MVP from dbGap[67] for inclusion in the all-ancestry meta-analyses for F-ALL (MVP PheCode=626.8) and M-ALL (MVP PheCode=609). Due to inflation in the P-values of variants with MAF<1%, we only retained common variants with MAF>1%. We did not include MVP EUR-only summary statistics in our EUR-only meta-analyses as we observed significant allele frequency differences between the European ancestry populations in our study and those in MVP (261,296 variants with MAF<1% in our study populations had frequencies over 2.5 times higher in MVP, likely due to admixture with individuals of African ancestries).

Publicly available summary statistics for reproductive hormones. We downloaded publicly available GWAS summary statistics from studies without UK Biobank participants for FSH and LH[68,69,70], progesterone[71], oestradiol[71], and testosterone[69,71,72] from the GWAS Catalog[6] (Supp. Table 9). Summary statistics from Suhre *et al.* (2017)[68] were missing non-effect alleles, which were merged in by matching chromosome and genome position information from Ensembl hg19 variant call files[73]. These and the summary statistics from Prins *et al.* (2017)[72] were missing allele frequencies, which were merged in from chromosome, position, and allele-matched frequencies derived from European-ancestry individuals in the 1000 Genomes dataset[74]. All summary statistics on genome build hg19 were lifted over to hg38 using the UCSC liftOver tool[75] - between 97.3% and 99.7% of variants were successfully lifted over to the hg38 genome assembly.

Classification of lead meta-analysis variants

Hormone-associated variants were classified based on conditional analysis as (1) previously reported for the hormone of interest, (2) previously reported for any of 28 reproductive hormones, or (3) novel, based on SNP associations published in the GWAS Catalog as of 27 March 2023[6] (Supp. Table 17). We adapted criteria developed by Benonisdottir *et al.* (2016)[76] to classify novel variants as those that are not in LD with (r^2<0.1), and conditionally independent of ($P_{conditional}$<0.05), all published hormone-associated variants within 1 Mb; all other variants are considered to be previously reported. Conditional analysis was performed in GCTA-COJO[77], with LD information for European-ancestry individuals derived from the 1000 Genomes dataset[74].

For lead variants on the X chromosome and those from multi-ancestry analyses, for which estimating LD is more difficult due to differences in recombination rates and selection pressures between sexes and populations[78–80], we did not use the above LD-based classification system. Instead, a lead SNP was considered novel if it was not within 1 Mb of a published hormone-associated variant or if its effect was independent of published variants within a 1 Mb window ($P_{conditional}$<0.05), and reported if not.

Genome-wide genetic correlation

We estimated genetic correlations among the three categories of female infertility with significant heritability (Z>4)[3]: F-ALL, F-ANOV, and F-INCL, as well as among heritable female reproductive hormones (FSH and testosterone in females). We additionally obtained summary statistics from GWASs of thyroid stimulating hormone (TSH)[81] (sex-combined analysis, N=247,107 participants) and anti-Mullerian hormone (N=7,049 pre-menopausal participants)[82] from the largest publicly available European-ancestry studies to date. We also

tested for genetic correlations between infertility and reproductive hormones. Significant r_g after multiple testing was established at 2.38E-03 (FWER controlled at 5% across 21 tests using the Bonferroni method).

We collated European-ancestry GWAS summary statistics for four female reproductive disorders: (1) endometriosis from Rahmioglu *et al.* (2023)[83], (57,248 cases and 698,764 controls), (2) heavy menstrual bleeding by meta-analysing GWAS data from Gallagher *et al.* (2019)[84] and FinnGen data freeze 9[9] (31,309 cases and 318,510 controls), (3) PCOS by meta-analysing GWAS data from Tyrmi *et al.* (2022)[85] and a UKBB-based GWAS (14,467 cases and 430,267 controls), and (4) uterine fibroids by meta-analysing GWAS data generated by the Neale lab[1] and FinnGen data freeze 9[9], (42,446 cases and 588,955 controls). We additionally obtained summary statistics from a GWAS of spontaneous dizygotic (DZ) twinning (8,265 cases (mothers of DZ twins) and 264,567 controls; plus 26,252 DZ twins and 417,433 additional controls) from Mbarek *et al.* (2024), the largest European-ancestry study of female fecundity to date[86]. Significant r_g after multiple testing was established at 2.00E-03 (FWER controlled at 5% across 25 tests using the Bonferroni method).

We downloaded LD-score formatted summary statistics for European-ancestry individuals across 703 heritable phenotypes (Z>4) from the Neale lab round 2 collection[1]. The number of effectively independent phenotypes estimated by the Neale lab (M_{eff}=340) was used to establish significant r_g after multiple testing at 2.45E-05 (FWER controlled at 5% across 2,040 tests using the Bonferroni method).

Mendelian randomisation

We constructed genetic instruments for BMI, WHR, and WHRadjBMI with female-specific lead variants from a recent European-ancestry GWAS meta-analysis with a maximum sample size of 434,785 female participants[87]. SNPs were weighted by their female-specific effect sizes. The mean F-statistic across all SNPs in each instrument indicated sufficient strength for MR (BMI=61.3, WHR=74.8, WHRadjBMI=84.7, recommended>10[88]). As the instrument GWASs included participants from UK Biobank, we conducted a sensitivity analysis to avoid bias from sample overlap between instrument and outcome GWASs by constructing obesity-trait instruments from an earlier release of summary statistics from the GIANT Consortium without UKBB participants[89] (Supp. Table 11). As the WHRadjBMI instrument may be confounded due to adjustment for a correlated variable[90] (adjustment for BMI in the WHR GWAS), we performed multivariable MR with a joint instrument for BMI and WHR to estimate the BMI-adjusted causal effect of WHR on reproductive outcomes. We found no difference in effect estimates from MR conducted using an instrument for WHRadjBMI and multivariable MR (Supp. Table 19).

Hormone instruments were constructed for reproductive hormones in this study with F-statistic>10 (FSH-F=38.7, testosterone-F=66.1), using GWAS summary statistics from European-ancestry GWASs excluding UK Biobank participants to avoid sample overlap with outcome GWASs.

We also performed reciprocal MR to test the genetically predicted causal effects of infertility on obesity and reproductive hormone levels. Genetic instruments were constructed for

subtypes of infertility with F-statistic>10 (F-ALL=51.0, F-ANOV=36.2), using GWAS summary statistics from European-ancestry GWASs excluding UK Biobank participants to avoid sample overlap with outcome GWASs. We assessed the causal direction between each pair of traits tested with Steiger filtering of instruments and the Steiger directionality test.

We report results from the inverse-variance weighted (IVW) method, the MR-Egger method which is robust to horizontal pleiotropy[91], and the weighted median method which protects against outlier variants[92] (Supp Table 11).

Colocalisation

Only common variants (MAF>1%) within windows of +/- 50 kb around each lead variant for an infertility or reproductive hormone trait were retained. For each pair of traits tested for colocalisation, we set the prior probabilities of variants in a locus being causally associated with trait 1 (p_1) and trait 2 (p_2) to 1E-04 (99% confidence in a true association), and the prior for joint association p_{12} to 1E-06 (assuming equal likelihood of shared and non-shared causal variants for each trait in a locus) as recommended by the developers of coloc[93]. We tested five hypotheses: H0=no association with either trait in region, H1=association with trait 1 in region, but not trait 2, H2=association with trait 2 in region, but not trait 1, H3=association with both traits in region, but different causal variants, and H4=association with both traits in region, and a shared causal variant. A pair of traits were considered to colocalise if posterior probability of H4>50% and the ratio of posterior probabilities of H4/H3>5[94,95].

Construction of LD scores for ovarian cell types

We identified two publicly available single-cell gene expression datasets for ovarian cell types: (1) from Fan *et al.* (2019), who performed single-cell RNA sequencing on ovarian tissue from five adult women undergoing fertility preservation procedures with 20,676 cells across 19 identified cell types[16], and (2) from Jin *et al.* (2022), who performed single-nucleus RNA sequencing on autopsy samples from four women (aged 49-54 years, with normal ovarian histology) with 42,568 cells across 8 identified cell types[17]. The datasets were aligned and filtered using the QC pipelines provided by the authors of each study, and clustered with identical parameters to replicate the results of each individual study. Gene sets for each cluster were identified as recommended by Finucane *et al.* (2018)[96] - briefly, we identified differential expression between the cells in each cluster and all other clusters by using the Wilcoxon rank sum test implemented in Seurat v3.0[97–99], and returned the top 10% of genes that are specifically expressed in each cluster (positive average log-fold-change values), ranked by differential expression *P*-value. We computed annotation-specific LD scores for these gene sets using hg38 coordinates for gene TSSs and TESs obtained from Ensembl[73], across 1 million HapMap3 variants[2] with LD information from European-ancestry individuals in the 1000 Genomes phase 3 dataset[74].

Overlaps with genetic regions under selection

Directional selection. We identified 54 genomic regions under directional selection from three previously reported genome-wide scans: (1) 39 regions from the Composite of Multiple

Signals (CMS) test, which infers historical selection on the order of the past 50,000 years[100], (2) 12 regions from an ancient DNA scan that uses inferences of allele frequency from ancient genomes to determine selection over the past 10,000 years[101], and (3) three regions from Singleton Density Scores (SDSs), which use the pattern of singleton variants to identify recent selection in the past 2,000 to 3,000 years[102]. For each genomic window under directional selection, we report the infertility-associated variants with the lowest *P*-value.

Singleton Density Scores. We downloaded publicly available SDSs for SNPs in the UK10K dataset[102] to report the highest SDS (positive selection of derived allele over ancestral allele in the past 2,000 to 3,000 years) and lowest SDS (negative selection) within the +/-10kb window around each infertility or hormone lead SNP. To calculate trait-SDS for each phenotype, we aligned each SDS to the trait-increasing allele rather than the derived allele[102]. For each lead variant window containing variants with extreme SDSs (top 97.5th %ile or bottom 2.5th %ile), we report the direction of selection with respect to the trait-increasing allele. Percentiles of SDSs were evaluated only on a subset of variants within 10kb of any variant reported in the GWAS Catalog to account for genomic context. Further, as variants that are sub-genome-wide significant for a trait may nonetheless be under selection, we calculated the genome-wide mean trait-SDS in each bin of 1000 variants, ranked by *P*-value for the trait association, following the protocol outlined by Field *et al.* (2016)[102].

Balancing selection. We accessed publicly available standardised BetaScan2 scores, which detect balancing selection using polymorphism and substitution data, for all SNPs in the 1000 Genomes dataset[103]. We tested whether the +/-10kb window around each infertility or hormone lead variant contained SNPs with scores in the 99th %ile of standardised BetaScan2 scores. Percentiles of SDSs were evaluated only on a subset of variants within 10kb of any variant reported in the GWAS Catalog to account for genomic context. For each lead variant window, we report the highest standardised BetaScan2 score and its percentile.

Exome sequencing variant and sample-level QC

We defined “high quality” variants as those MAF>0.001 and call rate ≥0.99 falling within the UK Biobank capture intervals plus 50 bp padding. These variants were used to evaluate sample-level metrics of mean call rate and depth. We retained samples satisfying all of the following:

- Genetic sex inferred as XX or XY
- Mean call rate ≥0.99.
- Mean coverage ≥20x.
- Not withdrawn.

Next, we removed variants satisfying at least one of the following criteria:

- The variant lies outside the UK Biobank capture plus 50 bp padding.
- The variant lies within a low complexity region.
- The variant lies within a segmental duplication.

Among this (sample, variant) set, we ran Hail's sample_qc()[104] to remove samples lying outside the median ± 4 median absolute deviations (MADs) within each super-population (see "Assigning population labels" in Methods section above). The QC protocol was split by UK Biobank WES tranche (50k, 200k, 450k) to guard against batch effects, as tranches were sequenced in separate runs. The following metrics were used for QC:

- Number of deletions (n_deletion).
- Number of insertions (n_insertion).
- Number of SNPs (n_snp).
- Ratio of insertions to deletions (r_insertion_deletion).
- Ratio of transitions to transversions (r_ti_tv).
- Ratio of heterozygous variants to homozygous alternate variants (r_het_hom_var).

Following MAD filtering (Supp. Figure 12, Supp. Table 18), 402,345 European samples were retained for analysis. For each sample, we excluded non-passing sites as described in Karzcewski *et al.* (2022)[105]. Briefly, an RF classifier was trained to distinguish true positives from false positive variants using a collection of allele and site annotations. Variants were assigned 'PASS' to maximise sensitivity and specificity across a series of readouts in trio data and precision-recall in two truth samples, after which samples with excess heterozygosity (defined as inbreeding coefficient<−0.3) were removed. Next, we removed low quality genotypes by filtering to the subset of genotypes with depth≥10 (5 among haploid calls), genotype quality≥20, and minor allele balance >0.2 for all alternate alleles for heterozygous genotypes. Following this filter, we remove variants that were not called as "high quality" among any sample.

Variant categories for gene-based tests

1. **High confidence pLoF**: high-confidence LoF variants, as defined by LOFTEE [106] (LOFTEE HC).
2. **Damaging missense/protein-altering**: at least one of:
    a. Variant annotated as missense/start-loss/stop-loss/in-frame indel and (REVEL≥0.773 or CADD≥28.1 (or both)),
    b. Any variant with SpliceAI delta score (DS)≥0.2 where SpliceAI DS the maximum of the set {DS_AG, DS_AL, DS_DG, DS_DL} for each annotated variant (where DS_AG, DS_AL, DS_DG and DS_DL are delta score (acceptor gain), delta score (acceptor loss), delta score (donor gain), and delta score (donor loss), respectively), or
    c. Low-confidence LoF variants, as defined by LOFTEE (LOFTEE LC)
3. **Other missense/protein-altering**: missense/start-loss/stop-loss/in-frame indel not categorised in (2) (Damaging missense/protein-altering).
4. **Synonymous**: synonymous variants with SpliceAI DS<0.2 in the gene (our 'control' set).

REVEL and CADD score cut-offs are chosen to reflect the supporting level for pathogenicity (PP3) from the American College of Medical Genetics and Genomics and the Association for Molecular Pathology (ACMG/AMP) criteria[107].

Replication of rare variant gene-level analyses in deCODE

Variant annotation. We used Variant Effect Predictor (VEP) to attribute predicted consequences to sequence variants. We classified as high-impact variants those predicted as start-lost, stop-gain, stop-lost, splice donor, splice acceptor or frameshift, collectively called loss-of-function (LOF) variants. We additionally explored missense variants with high conservation score (CADD > 25).

Gene burden models. We defined different models to group together various types of variants: (i) LOF variants, filtered with LOFTEE (LOFTEE HC); (ii) all LOF variants as defined above (LOF) (iii) LOF and missense variants when predicted deleterious by CADD score ≥ 25. For all models, we used MAF < 0.1% and MAF < 2% to select variants for analyses. Only high-quality sequence variants were considered for selection. We used the following quality metrics from GraphTyper and considered variants where ABHet > 0.175, ABHom > 0.85, QD > 6, QUAL > 10, PASS_ratio < 0.05 and AAscore > 0.8. To further estimate the quality of the sequence variants we regressed the alternative allele counts (AD) on the depth (DP) conditioned on the genotypes (GT). For a well-behaving sequence variant, the mean alternative allele count for a homozygous reference genotype should be 0, for a heterozygous genotype it should be DP/2 and for a homozygous alternative genotype it should be DP. Under the assumption of no sequencing or genotyping error, the expected value of AD should be DP conditioned on the genotype, in other words an identity line (slope 1 and intercept 0). Deviations from the identity line indicate that the sequence variant is spurious or somatic. We filter variants with slope less than 0.5.

Association analysis. For case-control analyses, we used logistic regression under an additive model to test for association between loss-of-function gene burdens and phenotypes, in which disease status was the dependent variable and genotype counts as the independent variable, using likelihood ratio test to compute two-sided P-values. Individuals were coded 1 if they carry any of the annotated variants in the autosomal gene being tested and 0 otherwise. Sequenced and imputed individuals were coded with genotype count 1 if they carry any LOF variants in the autosomal gene being tested and their expected allele count was > 0.9, and 0 otherwise. For imputed individuals LOF genotypes were only used if the LOF variant was imputed with imputation information > 0.7.

We further included variables indicating sequencing batches to remove batch effects. We used LD score regression intercepts to adjust the $\chi2$ statistics and avoid inflation due cryptic relatedness and stratification, using a set of 1.1 million variants. P-values were calculated from the adjusted $\chi2$ results. Quantitative traits were analysed using a linear mixed model implemented in BOLT-LMM[108].

Variables included as covariates were current age or age at death, sex, county of birth, blood sample availability, sequencing status and an indicator function for the overlap of the lifetime of the individual with the phenotype collection time span.

Comparison of gene-based burden effect sizes between UK Biobank and deCODE. As association analyses in UK Biobank were performed using SAIGE, which applies a MAF-based beta-weight, we calculated an unweighted burden beta to allow for comparison of effect sizes between UKBB discovery and deCODE replication results. We estimated the

unweighted effect size per gene as the ratio of the sum of the score statistics (oriented to the minor allele) to the sum of the variance in score statistics across all variants in the gene. This was repeated for all sets of variants used for the group tests (based on annotation and max-MAF).

Supplementary References

1. Neale lab round 2 GWAS blogpost. http://www.nealelab.is/blog/2019/10/24/updating-snp-heritability-results-from-4236-phenotypes-in-uk-biobank.
2. International HapMap 3 Consortium *et al.* Integrating common and rare genetic variation in diverse human populations. *Nature* **467**, 52–58 (2010).
3. Bulik-Sullivan, B. K. *et al.* LD Score regression distinguishes confounding from polygenicity in genome-wide association studies. *Nat. Genet.* **47**, 291–295 (2015).
4. GTEx Consortium. The Genotype-Tissue Expression (GTEx) project. *Nat. Genet.* **45**, 580–585 (2013).
5. Pers, T. H. *et al.* Biological interpretation of genome-wide association studies using predicted gene functions. *Nat. Commun.* **6**, 5890 (2015).
6. Welter, D. *et al.* The NHGRI GWAS Catalog, a curated resource of SNP-trait associations. *Nucleic Acids Res.* **42**, D1001–6 (2014).
7. Kivisild, T. *et al.* Patterns of genetic connectedness between modern and medieval Estonian genomes reveal the origins of a major ancestry component of the Finnish population. *Am. J. Hum. Genet.* **108**, 1792–1806 (2021).
8. Kumar, T. R. *et al.* Reproductive defects in gamma-glutamyl transpeptidase-deficient mice. *Endocrinology* **141**, 4270–4277 (2000).
9. Kurki, M. I. *et al.* FinnGen provides genetic insights from a well-phenotyped isolated population. *Nature* **613**, 508–518 (2023).
10. Cong, Y. *et al.* Ptk2b deletion improves mice folliculogenesis and fecundity via inhibiting follicle loss mediated by Erk pathway. *J. Cell. Physiol.* **236**, 1043–1053 (2021).
11. Dobzhansky, T. A review of some fundamental concepts and problems of population genetics. *Cold Spring Harb. Symp. Quant. Biol.* **20**, 1–15 (1955).
12. Lewontin, R. C. The Units of Selection. *Annu. Rev. Ecol. Syst.* **1**, 1–18 (1970).
13. Su, W. *et al.* Role of HSD17B13 in the liver physiology and pathophysiology. *Mol. Cell. Endocrinol.* **489**, 119–125 (2019).
14. Bernabeu, E. *et al.* Sex differences in genetic architecture in the UK Biobank. *Nat. Genet.* **53**, 1283–1289 (2021).
15. Martinez, G. & Garcia, C. Sexual selection and sperm diversity in primates. *Mol. Cell. Endocrinol.* **518**, 110974 (2020).
16. Fan, X. *et al.* Single-cell reconstruction of follicular remodeling in the human adult ovary. *Nat. Commun.* **10**, 3164 (2019).
17. Jin, C. *et al.* The regulatory landscapes of human ovarian ageing. *bioRxiv* (2022) doi:10.1101/2022.05.18.492547.
18. *NICE: Menopause, Diagnosis and Management – from Guideline to Practice*. https://thebms.org.uk/wp-content/uploads/2022/12/09-BMS-TfC-NICE-Menopause-Diagnosis-and-Management-from-Guideline-to-Practice-Guideline-Summary-NOV2022-A.pdf (2022).
19. Frei, O. *et al.* Bivariate causal mixture model quantifies polygenic overlap between complex traits beyond genetic correlation. *Nat. Commun.* **10**, 2417 (2019).

20. Hazel, W. N., Black, R., Smock, R. C., Sear, R. & Tomkins, J. L. An age-dependent ovulatory strategy explains the evolution of dizygotic twinning in humans. *Nat Ecol Evol* **4**, 987–992 (2020).
21. Kaufman, C. S. & Butler, M. G. Mutation in TNXB gene causes moderate to severe Ehlers-Danlos syndrome. *World J Med Genet* **6**, 17–21 (2016).
22. Hugon-Rodin, J., Lebègue, G., Becourt, S., Hamonet, C. & Gompel, A. Gynecologic symptoms and the influence on reproductive life in 386 women with hypermobility type ehlers-danlos syndrome: a cohort study. *Orphanet J. Rare Dis.* **11**, 124 (2016).
23. Tardif, S. *et al.* Zonadhesin is essential for species specificity of sperm adhesion to the egg zona pellucida. *J. Biol. Chem.* **285**, 24863–24870 (2010).
24. Infertility prevalence estimates, 1990–2021. https://www.who.int/publications/i/item/978920068315 (2023).
25. Fry, A. *et al.* Comparison of Sociodemographic and Health-Related Characteristics of UK Biobank Participants With Those of the General Population. *Am. J. Epidemiol.* **186**, 1026–1034 (2017).
26. Fertility treatment 2021: preliminary trends and figures. https://www.hfea.gov.uk/about-us/publications/research-and-data/fertility-treatment-2021-preliminary-trends-and-figures/.
27. Leitsalu, L. *et al.* Cohort Profile: Estonian Biobank of the Estonian Genome Center, University of Tartu. *Int. J. Epidemiol.* **44**, 1137–1147 (2015).
28. Sørensen, E. *et al.* Data Resource Profile: The Copenhagen Hospital Biobank (CHB). *Int. J. Epidemiol.* **50**, 719–720e (2021).
29. Nisbett, R. E. *et al.* Intelligence: new findings and theoretical developments. *Am. Psychol.* **67**, 130–159 (2012).
30. Rask-Andersen, M., Karlsson, T., Ek, W. E. & Johansson, Å. Modification of Heritability for Educational Attainment and Fluid Intelligence by Socioeconomic Deprivation in the UK Biobank. *Am. J. Psychiatry* **178**, 625–634 (2021).
31. Balbo, N., Billari, F. C. & Mills, M. Fertility in Advanced Societies: A Review of Research: La fécondité dans les sociétés avancées: un examen des recherches. *Eur. J. Popul.* **29**, 1–38 (2013).
32. Zirkin, B. R. & Papadopoulos, V. Leydig cells: formation, function, and regulation. *Biol. Reprod.* **99**, 101–111 (2018).
33. Burger, H. G. Androgen production in women. *Fertil. Steril.* **77 Suppl 4**, S3–5 (2002).
34. Fortune, J. E. & Armstrong, D. T. Androgen production by theca and granulosa isolated from proestrous rat follicles. *Endocrinology* **100**, 1341–1347 (1977).
35. Liu, Y. X. & Hsueh, A. J. Synergism between granulosa and theca-interstitial cells in estrogen biosynthesis by gonadotropin-treated rat ovaries: studies on the two-cell, two-gonadotropin hypothesis using steroid antisera. *Biol. Reprod.* **35**, 27–36 (1986).
36. Wen, X., Li, D., Tozer, A. J., Docherty, S. M. & Iles, R. K. Estradiol, progesterone, testosterone profiles in human follicular fluid and cultured granulosa cells from luteinized pre-ovulatory follicles. *Reprod. Biol. Endocrinol.* **8**, 117 (2010).
37. Kinnear, H. M. *et al.* The ovarian stroma as a new frontier. *Reproduction* **160**, R25–R39 (2020).
38. Samimi, M. *et al.* The Effects of Synbiotic Supplementation on Metabolic Status in Women With Polycystic Ovary Syndrome: a Randomized Double-Blind Clinical Trial. *Probiotics Antimicrob. Proteins* **11**, 1355–1361 (2019).
39. Zhou, Y., Ding, X. & Wei, H. Reproductive immune microenvironment. *J. Reprod. Immunol.* **152**, 103654 (2022).

40. Petersen, B.-S., Fredrich, B., Hoeppner, M. P., Ellinghaus, D. & Franke, A. Opportunities and challenges of whole-genome and -exome sequencing. *BMC Genet.* **18**, 14 (2017).
41. Saloniemi, T., Jokela, H., Strauss, L., Pakarinen, P. & Poutanen, M. The diversity of sex steroid action: novel functions of hydroxysteroid (17β) dehydrogenases as revealed by genetically modified mouse models. *J. Endocrinol.* **212**, 27–40 (2012).
42. Park, J.-H. *et al.* Distribution of allele frequencies and effect sizes and their interrelationships for common genetic susceptibility variants. *Proc. Natl. Acad. Sci. U. S. A.* **108**, 18026–18031 (2011).
43. Ruth, K. S. *et al.* Using human genetics to understand the disease impacts of testosterone in men and women. *Nat. Med.* **26**, 252–258 (2020).
44. Tchernof, A. *et al.* Androgens and the Regulation of Adiposity and Body Fat Distribution in Humans. *Compr. Physiol.* **8**, 1253–1290 (2018).
45. Loh, N. Y. *et al.* Sex hormones, adiposity, and metabolic traits in men and women: a Mendelian randomisation study. *Eur. J. Endocrinol.* **186**, 407–416 (2022).
46. Palmer, B. F. & Clegg, D. J. The sexual dimorphism of obesity. *Mol. Cell. Endocrinol.* **402**, 113–119 (2015).
47. Schulze, M. B. & Stefan, N. Genetic Predisposition to Abdominal Adiposity and Cardiometabolic Risk. *JAMA* **317**, 2334 (2017).
48. Goossens, G. H., Jocken, J. W. E. & Blaak, E. E. Sexual dimorphism in cardiometabolic health: the role of adipose tissue, muscle and liver. *Nat. Rev. Endocrinol.* **17**, 47–66 (2021).
49. Agarwal, A., Mulgund, A., Hamada, A. & Chyatte, M. R. A unique view on male infertility around the globe. *Reprod. Biol. Endocrinol.* **13**, 37 (2015).
50. Nakamura, N. *et al.* Disruption of a spermatogenic cell-specific mouse enolase 4 (eno4) gene causes sperm structural defects and male infertility. *Biol. Reprod.* **88**, 90 (2013).
51. Nawaz, S. *et al.* A variant in sperm-specific glycolytic enzyme enolase 4 (ENO4) causes human male infertility. *J. Gene Med.* e3583 (2023) doi:10.1002/jgm.3583.
52. Sudlow, C. *et al.* UK biobank: an open access resource for identifying the causes of a wide range of complex diseases of middle and old age. *PLoS Med.* **12**, e1001779 (2015).
53. Bycroft, C. *et al.* The UK Biobank resource with deep phenotyping and genomic data. *Nature* **562**, 203–209 (2018).
54. Zhang, D., Dey, R. & Lee, S. Fast and robust ancestry prediction using principal component analysis. *Bioinformatics* **36**, 3439–3446 (2020).
55. Dey, R. & Lee, S. Asymptotic properties of principal component analysis and shrinkage-bias adjustment under the generalized spiked population model. *J. Multivar. Anal.* **173**, 145–164 (2019).
56. randomForest: Breiman and Cutler's Random Forests for Classification and Regression. *Comprehensive R Archive Network (CRAN)* https://cran.r-project.org/web/packages/randomForest.
57. Fraser, A. *et al.* Cohort Profile: the Avon Longitudinal Study of Parents and Children: ALSPAC mothers cohort. *Int. J. Epidemiol.* **42**, 97–110 (2013).
58. McCarthy, S. *et al.* A reference panel of 64,976 haplotypes for genotype imputation. *Nat. Genet.* **48**, 1279–1283 (2016).
59. Loh, P.-R. *et al.* Efficient Bayesian mixed-model analysis increases association power in large cohorts. *Nat. Genet.* **47**, 284–290 (2015).
60. Hansen, T. F. *et al.* DBDS Genomic Cohort, a prospective and comprehensive resource for integrative and temporal analysis of genetic, environmental and lifestyle factors affecting health of blood donors. *BMJ Open* **9**, e028401 (2019).

61. Kong, A. *et al.* Detection of sharing by descent, long-range phasing and haplotype imputation. *Nat. Genet.* **40**, 1068–1075 (2008).
62. Gudbjartsson, D. F. *et al.* Large-scale whole-genome sequencing of the Icelandic population. *Nat. Genet.* **47**, 435–444 (2015).
63. Reisberg, S. *et al.* Translating genotype data of 44,000 biobank participants into clinical pharmacogenetic recommendations: challenges and solutions. *Genet. Med.* **21**, 1345–1354 (2019).
64. Mbatchou, J. *et al.* Computationally efficient whole-genome regression for quantitative and binary traits. *Nat. Genet.* **53**, 1097–1103 (2021).
65. Finer, S. *et al.* Cohort Profile: East London Genes & Health (ELGH), a community-based population genomics and health study in British Bangladeshi and British Pakistani people. *Int. J. Epidemiol.* **49**, 20–21i (2020).
66. Taliun, D. *et al.* Sequencing of 53,831 diverse genomes from the NHLBI TOPMed Program. *Nature* **590**, 290–299 (2021).
67. Verma, A. *et al.* Diversity and scale: Genetic architecture of 2068 traits in the VA Million Veteran Program. *Science* **385**, eadj1182 (2024).
68. Suhre, K. *et al.* Connecting genetic risk to disease end points through the human blood plasma proteome. *Nat. Commun.* **8**, 14357 (2017).
69. Dennis, J. K. *et al.* Clinical laboratory test-wide association scan of polygenic scores identifies biomarkers of complex disease. *Genome Med.* **13**, 6 (2021).
70. Thareja, G. *et al.* Differences and commonalities in the genetic architecture of protein quantitative trait loci in European and Arab populations. *Hum. Mol. Genet.* **32**, 907–916 (2023).
71. Pott, J. *et al.* Genetic Association Study of Eight Steroid Hormones and Implications for Sexual Dimorphism of Coronary Artery Disease. *J. Clin. Endocrinol. Metab.* **104**, 5008–5023 (2019).
72. Prins, B. P. *et al.* Genome-wide analysis of health-related biomarkers in the UK Household Longitudinal Study reveals novel associations. *Sci. Rep.* **7**, 11008 (2017).
73. Martin, F. J. *et al.* Ensembl 2023. *Nucleic Acids Res.* **51**, D933–D941 (2023).
74. 1000 Genomes Project Consortium *et al.* A global reference for human genetic variation. *Nature* **526**, 68–74 (2015).
75. Kuhn, R. M., Haussler, D. & Kent, W. J. The UCSC genome browser and associated tools. *Brief. Bioinform.* **14**, 144–161 (2013).
76. Benonisdottir, S. *et al.* Epigenetic and genetic components of height regulation. *Nat. Commun.* **7**, 13490 (2016).
77. Yang, J., Lee, S. H., Goddard, M. E. & Visscher, P. M. GCTA: a tool for genome-wide complex trait analysis. *Am. J. Hum. Genet.* **88**, 76–82 (2011).
78. Schaffner, S. F. The X chromosome in population genetics. *Nat. Rev. Genet.* **5**, 43–51 (2004).
79. Veeramah, K. R. & Novembre, J. Demographic events and evolutionary forces shaping European genetic diversity. *Cold Spring Harb. Perspect. Biol.* **6**, a008516 (2014).
80. Hinch, A. G., Altemose, N., Noor, N., Donnelly, P. & Myers, S. R. Recombination in the human Pseudoautosomal region PAR1. *PLoS Genet.* **10**, e1004503 (2014).
81. Williams, A. T. *et al.* Genome-wide association study of thyroid-stimulating hormone highlights new genes, pathways and associations with thyroid disease. *Nat. Commun.* **14**, 6713 (2023).

82. Verdiesen, R. M. G. *et al.* Genome-wide association study meta-analysis identifies three novel loci for circulating anti-Müllerian hormone levels in women. *Hum. Reprod.* **37**, 1069–1082 (2022).
83. Rahmioglu, N. *et al.* The genetic basis of endometriosis and comorbidity with other pain and inflammatory conditions. *Nat. Genet.* **55**, 423–436 (2023).
84. Gallagher, C. S. *et al.* Genome-wide association and epidemiological analyses reveal common genetic origins between uterine leiomyomata and endometriosis. *Nat. Commun.* **10**, 4857 (2019).
85. Tyrmi, J. S. *et al.* Leveraging Northern European population history: novel low-frequency variants for polycystic ovary syndrome. *Hum. Reprod.* **37**, 352–365 (2022).
86. Mbarek, H. *et al.* Genome-wide association study meta-analysis of dizygotic twinning illuminates genetic regulation of female fecundity. *Hum. Reprod.* **39**, 240–257 (2024).
87. Pulit, S. L. *et al.* Meta-analysis of genome-wide association studies for body fat distribution in 694 649 individuals of European ancestry. *Hum. Mol. Genet.* **28**, 166–174 (2019).
88. Burgess, S., Thompson, S. G. & CRP CHD Genetics Collaboration. Avoiding bias from weak instruments in Mendelian randomization studies. *Int. J. Epidemiol.* **40**, 755–764 (2011).
89. Locke, A. E. *et al.* Genetic studies of body mass index yield new insights for obesity biology. *Nature* **518**, 197–206 (2015).
90. Hartwig, F. P., Tilling, K., Davey Smith, G., Lawlor, D. A. & Borges, M. C. Bias in two-sample Mendelian randomization when using heritable covariable-adjusted summary associations. *Int. J. Epidemiol.* **50**, 1639–1650 (2021).
91. Bowden, J., Davey Smith, G. & Burgess, S. Mendelian randomization with invalid instruments: effect estimation and bias detection through Egger regression. *Int. J. Epidemiol.* **44**, 512–525 (2015).
92. Bowden, J., Davey Smith, G., Haycock, P. C. & Burgess, S. Consistent Estimation in Mendelian Randomization with Some Invalid Instruments Using a Weighted Median Estimator. *Genet. Epidemiol.* **40**, 304–314 (2016).
93. Wallace, C. Eliciting priors and relaxing the single causal variant assumption in colocalisation analyses. *PLoS Genet.* **16**, e1008720 (2020).
94. Schmiedel, B. J. *et al.* COVID-19 genetic risk variants are associated with expression of multiple genes in diverse immune cell types. *Nat. Commun.* **12**, 6760 (2021).
95. Giambartolomei, C. *et al.* Bayesian test for colocalisation between pairs of genetic association studies using summary statistics. *PLoS Genet.* **10**, e1004383 (2014).
96. Finucane, H. K. *et al.* Heritability enrichment of specifically expressed genes identifies disease-relevant tissues and cell types. *Nat. Genet.* **50**, 621–629 (2018).
97. Stuart, T. *et al.* Comprehensive Integration of Single-Cell Data. *Cell* **177**, 1888–1902.e21 (2019).
98. Butler, A., Hoffman, P., Smibert, P., Papalexi, E. & Satija, R. Integrating single-cell transcriptomic data across different conditions, technologies, and species. *Nat. Biotechnol.* **36**, 411–420 (2018).
99. Satija, R., Farrell, J. A., Gennert, D., Schier, A. F. & Regev, A. Spatial reconstruction of single-cell gene expression data. *Nat. Biotechnol.* **33**, 495–502 (2015).
100. Grossman, S. R. *et al.* Identifying recent adaptations in large-scale genomic data. *Cell* **152**, 703–713 (2013).
101. Mathieson, I. *et al.* Genome-wide patterns of selection in 230 ancient Eurasians. *Nature* **528**, 499–503 (2015).

102. Field, Y. *et al.* Detection of human adaptation during the past 2000 years. *Science* **354**, 760–764 (2016).
103. Siewert, K. M. & Voight, B. F. BetaScan2: Standardized Statistics to Detect Balancing Selection Utilizing Substitution Data. *Genome Biol. Evol.* **12**, 3873–3877 (2020).
104. hail 0.2 documentation. https://hail.is/docs/0.2/.
105. Karczewski, K. J. *et al.* Systematic single-variant and gene-based association testing of thousands of phenotypes in 394,841 UK Biobank exomes. *Cell Genom* **2**, 100168 (2022).
106. Karczewski, K. J. *et al.* The mutational constraint spectrum quantified from variation in 141,456 humans. *Nature* **581**, 434–443 (2020).
107. Richards, S. *et al.* Standards and guidelines for the interpretation of sequence variants: a joint consensus recommendation of the American College of Medical Genetics and Genomics and the Association for Molecular Pathology. *Genet. Med.* **17**, 405–424 (2015).
108. Loh, P.-R., Kichaev, G., Gazal, S., Schoech, A. P. & Price, A. L. Mixed-model association for biobank-scale datasets. *Nat. Genet.* **50**, 906–908 (2018).
