# Supplementary material for: Genome-wide analyses identify 25 infertility loci and relationships with reproductive traits across the allele frequency spectrum
Source: Nat Genet. 2025 Apr 14;57(5):1107–18. doi: 10.1038/s41588-025-02156-8 (PMC12081293; doi:10.1038/s41588-025-02156-8)
Supplement: Supplementary file 2 — Reporting Summary [file 41588_2025_2156_MOESM2_ESM.pdf]

nature portfolio

Corresponding author(s): Samvida S. Venkatesh & Cecilia M. Lindgren

Last updated by author(s): 12/20/2024

Reporting Summary

Nature Portfolio wishes to improve the reproducibility of the work that we publish. This form provides structure for consistency and transparency in reporting. For further information on Nature Portfolio policies, see our Editorial Policies and the Editorial Policy Checklist.

Statistics

For all statistical analyses, confirm that the following items are present in the figure legend, table legend, main text, or Methods section.

n/a	Confirmed	
☐	☒	The exact sample size (*n*) for each experimental group/condition, given as a discrete number and unit of measurement
☐	☒	A statement on whether measurements were taken from distinct samples or whether the same sample was measured repeatedly
☐	☒	The statistical test(s) used AND whether they are one- or two-sided<br>*Only common tests should be described solely by name; describe more complex techniques in the Methods section.*
☐	☒	A description of all covariates tested
☐	☒	A description of any assumptions or corrections, such as tests of normality and adjustment for multiple comparisons
☐	☒	A full description of the statistical parameters including central tendency (e.g. means) or other basic estimates (e.g. regression coefficient) AND variation (e.g. standard deviation) or associated estimates of uncertainty (e.g. confidence intervals)
☐	☒	For null hypothesis testing, the test statistic (e.g. *F*, *t*, *r*) with confidence intervals, effect sizes, degrees of freedom and *P* value noted<br>*Give P values as exact values whenever suitable.*
☐	☒	For Bayesian analysis, information on the choice of priors and Markov chain Monte Carlo settings
☒	☐	For hierarchical and complex designs, identification of the appropriate level for tests and full reporting of outcomes
☒	☐	Estimates of effect sizes (e.g. Cohen's *d*, Pearson's *r*), indicating how they were calculated

Our web collection on statistics for biologists contains articles on many of the points above.

Software and code

Policy information about availability of computer code

Data collection	No software was used specifically for data collection in this study; each biobank project that provided data for this study reported their data collection strategies in flagship papers that are cited in the Supplementary Methods section of this manuscript (Supplementary Information p30-33).
Data analysis	Each analysis described uses publicly available software as detailed in the relevant sub-sections of the Methods in the main manuscript and Supplemental Text.<br><br>Genome-wide association testing was performed independently be each cohort using REGENIE, SAIGE, or custom pipelines (versions differ by cohort and are reported in Supplementary Methods). GWASs in CHB/DBDS were performed with SAIGE v1.18. GWASs in EstBB were performed using the REGENIE software v3.0.3. GWASs in FinnGen was performed using the REGENIE software v2.2.4. GWASs in G&H were performed using the REGENIE software. GWASs in ALSPAC were performed using BOLT-LMM. GWASs in UK Biobank were performed using the REGENIE software v3.0.3.<br><br>GWAS meta-analysis was performed in METAL (version released 2011-03-25). Conditional analysis to identify novel SNPs was performed in GCTA-COJO v1.91.5. LD-score regression for heritability and genetic correlation analyses was performed using LDSC v1.0.0. Local genetic correlations were tested using LAVA v1.8.0. Polygenic overlap was assessed with MiXeR v1.3. Mendelian randomisation was performed using the TwoSampleMR v0.5.7 package in R. Colocalisation analyses were performed using the coloc v5.1.0 package in R. Publicly available single-cell RNA sequencing data was processed using the Seurat v3.0 package in R. Exome sequencing variant annotation was performed using VEP v105 with plugins for LOFTEE v1.04_GRCh38 and dbNSFP (annotating variants with CADD v1.6 and REVEL using dbNSFP4.3). SpliceAI v1.3 gene annotations were used to ensure alignment between VEP and SpliceAI. Rare variant genetic association testing using the exome sequenicng data was conducted in SAIGE version wzhou88/saige:1.1.9. The genotype data was LD pruned using PLINK v2.0.

All other code to generate figures and tables was written in R v4.1.2.

Code Availability - All code used in this study is available through GitHub - https://github.com/lindgrengroup/infertility_hormones and Zenodo - https://doi.org/10.5281/zenodo.14528258.

For manuscripts utilizing custom algorithms or software that are central to the research but not yet described in published literature, software must be made available to editors and reviewers. We strongly encourage code deposition in a community repository (e.g. GitHub). See the Nature Portfolio guidelines for submitting code & software for further information.

Data

Policy information about availability of data

All manuscripts must include a data availability statement. This statement should provide the following information, where applicable:

- Accession codes, unique identifiers, or web links for publicly available datasets
- A description of any restrictions on data availability
- For clinical datasets or third party data, please ensure that the statement adheres to our policy

Cohorts may be contacted individually for access to raw data. Meta-analysis summary statistics for all phenotypes are available through the GWAS Catalog study accession numbers GCST90483463 to GCST90483502.

Research involving human participants, their data, or biological material

Policy information about studies with human participants or human data. See also policy information about sex, gender (identity/presentation), and sexual orientation and race, ethnicity and racism.

Reporting on sex and gender

All analyses reported in this study are disaggregated by sex (i.e. male-specific and female-specific analyses only). Each cohort only retained individuals whose self-reported sex matched genetically-inferred sex from the genotyping or exome sequencing data (XX chromosomes for females and XY chromosomes for males). Sample sizes for each sex within each cohort are reported in Supp. Tables 2 (for infertility analyses) and 9 (for hormone analyses). Throughout the paper, we use the terms woman/female and man/male inter-changeably to refer to individuals' genetically inferred sex. We recognise the limitations of this binary analysis that excludes participants whose self-reported sex or gender did not match their genetically-inferred sex.

Reporting on race, ethnicity, or other socially relevant groupings

As genetic association testing must account for linkage disequilibrium (LD) patterns that differ based on ancestral population histories, we conducted analyses separately in different population groups. Each biobank project that provided data for this study assigned genetically-inferred population labels (referred to in this paper as "ancestry") to samples based on different strategies reported in their flagship papers that are cited in the Methods section of this manuscript (Supp. Text p5-6). Sample sizes for each ancestry group are specified in Supp. Tables 2 (for infertility analyses) and 9 (for hormone analyses). For the UK Biobank, we assigned sample population labels by training a random forest (RF) classifier using the 1000 Genomes 'super-population' labels. We first ran principal components analysis (PCA) on unrelated individuals in the 1000 Genomes project dataset, subset to LD-pruned autosomal variants. Samples in the UKBB genotyping data are projected onto this PCA space, ensuring that we correctly account for shrinkage bias in the projection. Next, we used the 'super-population' labels (AFR=Africans, AMR=Admixed Americans, EAS=East Asians, Europeans=EUR, South Asians=SAS) of the 1000 Genomes dataset to train a RF classifier, using the randomForest (4.6) library in R, and predicted the super-population for each of the UKBB samples. Samples with classification probability >0.99 were retained for downstream analysis. Genome-wide association studies were performed separately in each genetically-inferred ancestral group. Meta-analyses were performed in two sets: (1) EUR-only subset, (2) all-ancestries. Any analyses that are dependent on LD are performed in the EUR-only subset and are flagged as such (Figure 1).

Population characteristics

Population characteristics (sample size, genotyping information, and types of records used for case/control ascertainment) for each biobank project included in this study are described in Supplementary Information pages 30-33. Sex- and ancestry-specific sample sizes are reported in Supp. Tables 2 and 9.

Recruitment

Each biobanking project used in this study followed different recruitment protocols, which are detailed in their flagship papers. A brief summary is provided in the Supplementary Information pages 30-33 along with citations to the flagship papers of each cohort.

Ethics oversight

This study was conducted using data from seven research studies, each with separate ethics approval. 1. UK Biobank has approval from the North West Multi-centre Research Ethics Committee (MREC) as a Research Tissue Bank (RTB) approval. 2. Copenhagen Hospital Biobank is classified as a biobank for future research. It is part of the Danish National Biobank and has been approved by the Danish Data Protection Agency (general approval number 2012-58-0004, and local number: RH-2007-30-4129/I-suite 00678). The Danish Blood Donor Study was approved by the Central Denmark (1-10-72-95-13) and Zealand (SJ-740) Regional Committees on Health Research Ethics and the Data Protection Agency (P-2019-99). The DBDS GWA study was approved by the Danish National Committee on Health Research Ethics (1700407). SCANDAT was approved by the Data Protection Agency (2008-54-0472). The DBDS was conducted in accordance with the ethical principles outlined in the Declaration of Helsinki. 3. Genes & Health operates under ethical approval, 14/LO/1240, from London South East NRES Committee of the Health Research Authority, dated 16 September 2014. 4. The activities of the Estonian Biobank are regulated by the Human Genes Research Act, which was adopted in 2000 specifically for the operations of the EstBB. Individual level data analysis in the EstBB was carried out under ethical approval 1.1-12/624 from the Estonian Committee on Bioethics and Human Research (Estonian Ministry of Social Affairs), using data according to release application 3-10/GI/10790 from the Estonian Biobank. 5. Patients and control subjects in FinnGen provided informed consent for biobank research, based on the Finnish Biobank Act. Alternatively, separate research cohorts, collected prior the Finnish Biobank Act

came into effect (in September 2013) and start of FinnGen (August 2017), were collected based on study-specific consents and later transferred to the Finnish biobanks after approval by Fimea (Finnish Medicines Agency), the National Supervisory Authority for Welfare and Health. Recruitment protocols followed the biobank protocols approved by Fimea. The Coordinating Ethics Committee of the Hospital District of Helsinki and Uusimaa (HUS) statement number for the FinnGen study is Nr HUS/990/2017. The FinnGen study is approved by Finnish Institute for Health and Welfare (permit numbers: THL/2031/6.02.00/2017, THL/1101/5.05.00/2017, THL/341/6.02.00/2018, THL/2222/6.02.00/2018, THL/283/6.02.00/2019, THL/1721/5.05.00/2019 and THL/1524/5.05.00/2020), Digital and population data service agency (permit numbers: VRK43431/2017-3, VRK/6909/2018-3, VRK/4415/2019-3), the Social Insurance Institution (permit numbers: KELA 58/522/2017, KELA 131/522/2018, KELA 70/522/2019, KELA 98/522/2019, KELA 134/522/2019, KELA 138/522/2019, KELA 2/522/2020, KELA 16/522/2020), Findata permit numbers THL/2364/14.02/2020, THL/4055/14.06.00/2020, THL/3433/14.06.00/2020, THL/4432/14.06/2020, THL/5189/14.06/2020, THL/5894/14.06.00/2020, THL/6619/14.06.00/2020, THL/209/14.06.00/2021, THL/688/14.06.00/2021, THL/1284/14.06.00/2021, THL/1965/14.06.00/2021, THL/5546/14.02.00/2020, THL/2658/14.06.00/2021, THL/4235/14.06.00/2021, Statistics Finland (permit numbers: TK-53-1041-17 and TK/143/07.03.00/2020 (earlier TK-53-90-20) TK/1735/07.03.00/2021, TK/3112/07.03.00/2021) and Finnish Registry for Kidney Diseases permission/extract from the meeting minutes on 4th July 2019. 6. Ethical approval for the study was obtained from the Avon Longitudinal Study of Parents and Children (ALSPAC) Ethics and Law Committee and the Local Research Ethics Committees. 7. The deCode Genetics study was approved by the Icelandic National Bioethics Committee (approval no. VSN-19-023).

Note that full information on the approval of the study protocol must also be provided in the manuscript.

Field-specific reporting

Please select the one below that is the best fit for your research. If you are not sure, read the appropriate sections before making your selection.

☒ Life sciences ☐ Behavioural & social sciences ☐ Ecological, evolutionary & environmental sciences

For a reference copy of the document with all sections, see nature.com/documents/nr-reporting-summary-flat.pdf

Life sciences study design

All studies must disclose on these points even when the disclosure is negative.

Sample size

Sample sizes for infertility GWAS meta-analyses: up to 42,629 cases / 740,619 controls in women, and 10,886 cases / 995,982 controls in men; sample sizes for hormone GWAS meta-analyses: up to 246,862 for testosterone in women, and 243,951 for testosterone in men. Detailed sample sizes for all subtypes of infertility and hormones are outlined in Supp. Tables 2 & 9. Sample sizes were not pre-determined as there was no minimum effect size we were trying to capture. We were looking to identify the smallest significant effect size possible with the available samples. Each of the seven study cohorts provided the maximum number of samples possible for each phenotype.

Data exclusions

Each cohort performed variant- and sample-specific quality control before genome-wide association testing; these are described in Supp. Text p8-10. Prior to meta-analysis, we performed additional variant-specific QC to retain variants that met the following criteria: (1) on the autosomes or X chromosome, (2) with imputation information score >0.8, (3) bi-allelic variants with A,C,G,T alleles, (4) with standard errors <10 and P-values in [0,1], and (5) without duplicate entries. Variants and samples for exome sequencing analyses underwent separate quality control, as outlined on p32 of the main text and p11 of Supp. Text. Briefly, we first considered an initial set of "high quality" variants to evaluate the mean call rate and depth of coverage for each sample. We then ran a sample and variant level pre-filtering step and calculated sample-level QC metrics. Using these metrics, we removed sample outliers based on median absolute deviation (MAD) thresholds, and excluded sites which did not pass variant QC according to Karzcewski et al. (2022). We then applied a genotype-level filter using genotype quality (GQ), depth (DP), and heterozygote allele balance (AB). The resultant high-quality European call set consisted of 402,375 samples and 25,229,669 variants.

Replication

As this study reports a meta-analysis of GWASs from several biobanking projects, we did not seek additional replication. For all lead variants reported in the manuscript, we report the heterogeneity in effect sizes among participating cohorts; we also flag if a lead variant was only reported in one cohort. We sought replication of the exome-sequencing results (discovery in UK Biobank) at the variant level and gene level. At the variant level, we looked up the effects of rare variants in EUR-ancestry GWAS meta-analysis excluding UKB participants (hence, replicated once). At the gene level, we report the results from gene-burden testing in the deCODE and Genes & Health datasets (hence, replicated twice). Supp. Tables 15, 20, and 21 include replication results.

In discovery, gene-burden analyses implicated the PLEKHG4 gene for F-EXCL (burden test OR (95% CI)=1.04 (1.02-1.06) when aggregated across all variant annotations with MAF<1%, Cauchy P=2.47E-07). This association did not replicate in the deCODE or Genes & Health (G&H) datasets (P>0.05).

We discovered 43 gene-trait pairs associated with testosterone with Cauchy P<5E-06 in UK Biobank, thirteen (30.2%) of which replicate at nominal significance (P<0.05), and two (4.65%) at Bonferroni-adjusted significance (P<6.85E-04) in either the deCODE or G&H datasets with consistent directions of effect.

Randomization

The current work was not an experimental study so did not require randomisation into experimental groups.

Blinding

The current work was not an experimental study so did not require blinding to group allocation during data collection or analysis.

Reporting for specific materials, systems and methods

We require information from authors about some types of materials, experimental systems and methods used in many studies. Here, indicate whether each material, system or method listed is relevant to your study. If you are not sure if a list item applies to your research, read the appropriate section before selecting a response.

Materials & experimental systems

n/a	Involved in the study
☒	☐ Antibodies
☒	☐ Eukaryotic cell lines
☒	☐ Palaeontology and archaeology
☒	☐ Animals and other organisms
☒	☐ Clinical data
☒	☐ Dual use research of concern
☒	☐ Plants

Methods

n/a	Involved in the study
☒	☐ ChIP-seq
☒	☐ Flow cytometry
☒	☐ MRI-based neuroimaging

Plants

Seed stocks

Report on the source of all seed stocks or other plant material used. If applicable, state the seed stock centre and catalogue number. If plant specimens were collected from the field, describe the collection location, date and sampling procedures.

Novel plant genotypes

Describe the methods by which all novel plant genotypes were produced. This includes those generated by transgenic approaches, gene editing, chemical/radiation-based mutagenesis and hybridization. For transgenic lines, describe the transformation method, the number of independent lines analyzed and the generation upon which experiments were performed. For gene-edited lines, describe the editor used, the endogenous sequence targeted for editing, the targeting guide RNA sequence (if applicable) and how the editor was applied.

Authentication

Describe any authentication procedures for each seed stock used or novel genotype generated. Describe any experiments used to assess the effect of a mutation and, where applicable, how potential secondary effects (e.g. second site T-DNA insertions, mosiacism, off-target gene editing) were examined.
